# Rewiring melanoma cell fate: TRPM8 modulators trigger apoptosis and boost NK cell cytotoxicity

**DOI:** 10.1038/s41419-026-08469-8

**Published:** 2026-02-14

**Authors:** Carmela Sorrentino, Carmine Lauretta, Rosa D’Angiolo, Simona Musella, Pia Giovannelli, Alessia Bertamino, Carmine Ostacolo, Isabel Gomez Monterrey, Antimo Migliaccio, Gabriella Castoria, Marzia Di Donato

**Affiliations:** 1 Dept of Precision Medicine, University of Campania “L.Vanvitelli”, Naples, Italy; 2 Dept of Pharmacy, University of Salerno, Salerno, Italy; 3 Dept of Pharmacy, University of Naples “Federico II”, Naples, Italy; 4 Unit of Clinical and Molecular Pathology, University Hospital “L. Vanvitelli”, Naples, Italy

**Keywords:** Melanoma, Apoptosis

## Abstract

Metastatic melanoma is an aggressive malignancy with limited long-term treatment success due to therapeutic resistance and immune evasion. The transient receptor potential melastatin 8 (TRPM8) ion channel is overexpressed in melanoma but its role as therapeutic target remains unexplored. We investigated the anti-tumor effects of novel TRPM8 modulators in metastatic melanoma cells using viability assays, apoptosis markers, mitochondrial function analyses, reactive oxygen species (ROS) measurements and gene silencing. Their functional impact was further assessed in 3D melanoma organoids, clonogenic survival assays, and natural killer (NK) cell co-culture systems. TRPM8 is significantly overexpressed in metastatic melanoma, as compared with the normal counterparts. Its pharmacological inhibition with novel modulators selectively induces calcium-independent mitochondrial apoptosis characterized by ROS accumulation, mitochondrial membrane depolarization, cytochrome c release, and caspase-3 activation. This process involves activation of the ATM/p53 pathway and upregulation of pro-apoptotic proteins. Additionally, TRPM8 modulators increase expression of the NK cell-activating ligand ULBP1, enhancing melanoma susceptibility to NK-mediated cytotoxicity. Our study identifies TRPM8 as a promising biomarker in melanoma. Its targeting triggers mitochondrial cell death and simultaneously boosts NK cell recognition via ULBP1/NKG2D engagement. TRPM8 targeting in combination with immunotherapy might be, hence, further explored in clinical setting of advanced melanoma.

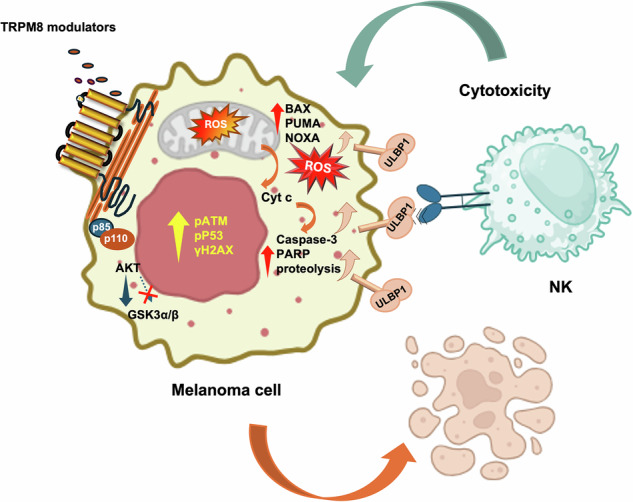

## Introduction

Cutaneous melanoma is among the most lethal forms of skin cancer and represents the second and third most frequent malignancy in males and females under the age of 50, respectively. The lifetime risk of developing melanoma is 1.5% in males and 1.2% in females, with a statistically significant increase in incidence observed annually in both sexes. Although early-stage melanomas are highly curable, advanced disease is characterized by rapid progression, metastatic dissemination, and poor prognosis due to its intrinsic or aquired resistance to cell death and immune evasion [1].

Although targeted therapies (e.g., BRAF and MEK inhibitors) and immune checkpoint blockers (e.g., anti–PD-1/PD-L1 and anti–CTLA-4 antibodies) have improved the clinical management of metastatic melanoma [2], many patients develop resistance or exhibit immune-related adverse events, leading to disease relapse or discontinuation of therapy [3]. These limitations underscore the need for complementary strategies that in addition to inducing direct cytotoxicity in tumor cells also enhance the recognition and clearance of tumor cells by the immune system.

The transient receptor potential melastatin-subfamily member 8 (TRPM8) channel, a calcium-permeable, non-selective cation channel, was initially characterized in sensory neurons as a cold thermosensor. Although largely neglected, it currently represents a promising target in many tumors, including melanoma [4–6]. TRPM8 is expressed in prostate, colon, breast, and lung cancers, where it promotes the growth, migration, and invasiveness of neoplastic cells [6–11]. Its pharmacological blockade or genetic silencing reduces proliferation and promotes apoptosis in prostate and colorectal cancer models [12–14], thus suggesting an oncogenic role for TRPM8.

TRPM8 is expressed in melanocytes and melanoma cells [15–19]. Most of the existing literature has focused on TRPM8 activation, showing that menthol or other agonists induce sustained Ca²⁺ influx and, in some contexts, cytotoxicity in melanoma cells [20]. Nevertheless, TRPM8 activation has also been associated with pro-survival effects under specific stress conditions, such as γ-irradiation, where TRPM8 silencing sensitizes melanoma cells to radiotherapy [18]. These apparently contrasting findings highlight that the biological consequences of TRPM8 modulation are highly context- and ligand-dependent. Importantly, while TRPM8 agonists have been extensively studied, the impact of TRPM8 antagonists or negative modulators on melanoma cell fate remains largely unexplored, and their downstream signaling, particularly beyond calcium flux, has not been defined.

This gap is critical because ion channel modulators often exert ligand-specific and non-canonical effects, including ROS generation, mitochondrial stress, and immunogenic remodeling. Therefore, clarifying how TRPM8 inhibition or negative modulation affects melanoma survival and immune susceptibility may reveal previously unrecognized therapeutic vulnerabilities.

In this study, we have investigated the anti-tumor potential of two novel TRPM8 antagonists, the compounds 4 and 9, in human metastatic melanoma cells. Our findings show that TRPM8 antagonists simultaneously induce p53-dependent apoptosis and enhance NK cell–mediated immunosurveillance via ULBP1–NKG2D engagement. In addition to identifying TRPM8 as a novel actionable target in melanoma, our findings strongly encourage its targeting in melanoma preclinical models. Pharmacological blockade of TRPM8, in combination with immunotherapies, might overcome the drug-resistance and enhance anti-tumor immunity in advanced melanoma.

## Results

### TRPM8 is overexpressed in melanoma and its pharmacological targeting induces cell death in metastatic melanoma cells

**Fig. 1:**
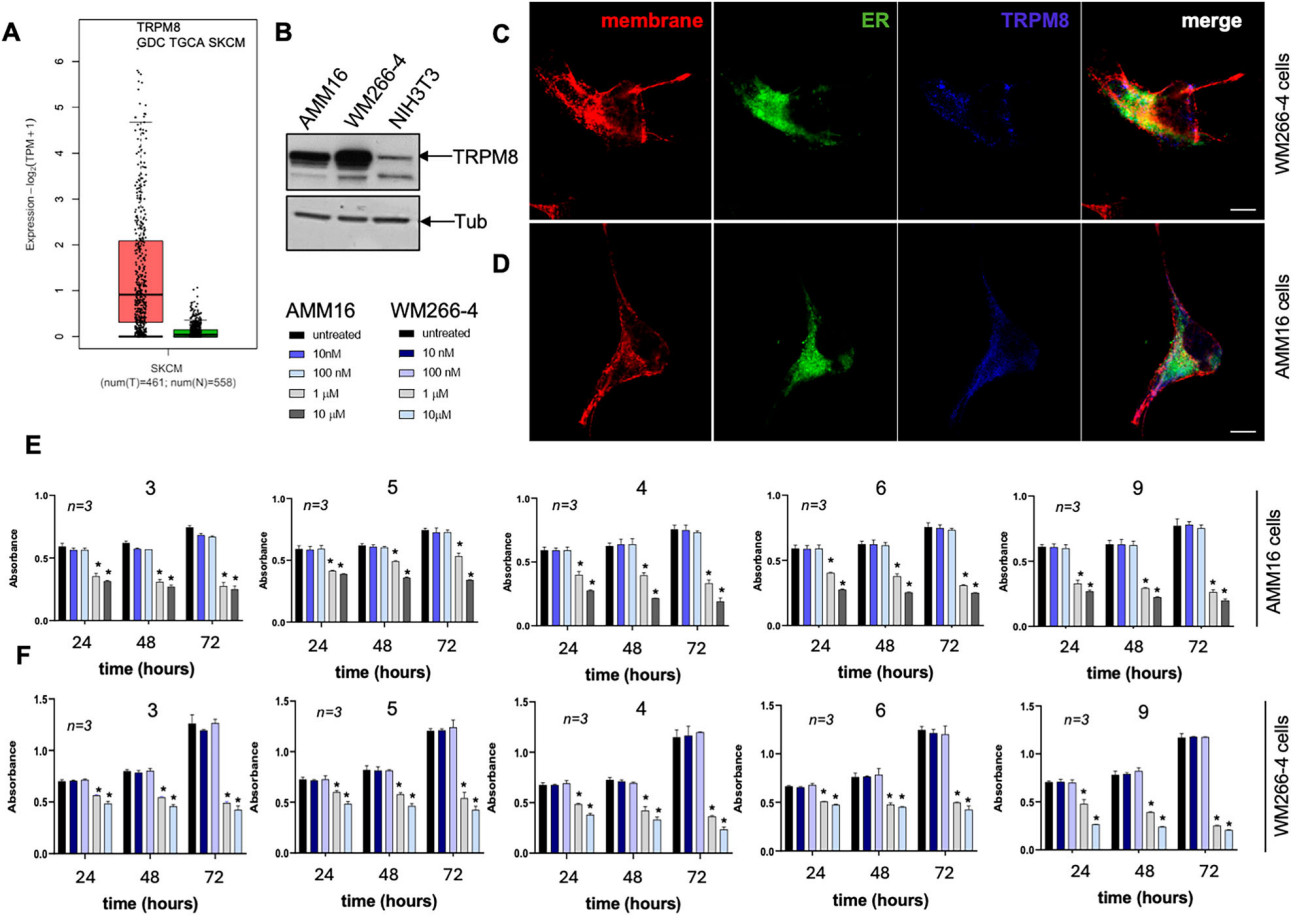
TRPM8 is overexpressed in metastatic melanoma cells and localizes to both the plasma membrane and endoplasmic reticulum. TRPM8 modulators reduce melanoma cell viability. **A** TRPM8 gene expression in melanoma tissues (red, T; *n* = *461*) vs. normal skin (green, N; *n* = *558*) from The Genomic Data Commons (GDC) Cancer Genome Atlas (TCGA) database. Data are presented as log2 TPM; *p* < 0.001 by unpaired *t*-test. **B** Representative westen blot showing TRPM8 protein expression in the indicated cell lines. Tubulin was used as a loading control. Immunofluorescence analysis of TRPM8 localization in WM266-4 (**C**) and AMM16 cells (**D**). Cells were stained with anti-TRPM8 antibody (blu), Endoplasmic reticulum (ER)-tracker (green) and Plasma Membrane-tracker (red). Merged images indicate TRPM8 distribution in both plasma membrane and ER compartments. Scale bar: 10 µm. Viability of AMM16 (**E**) and WM266-4 (**F**) cells untreated or treated with compounds 3, 5, 4, 6 and 9 at the concentrations indicated in the legends above. Absorbance values from WST-1 assays after 24, 48 and 72 h are reported. Data are expressed as mean ± standard deviations (SDs) from *n* independent experiments. ^*^p < 0.05 for the indicated time points vs. the corresponding untreated control.

**Fig. 2:**
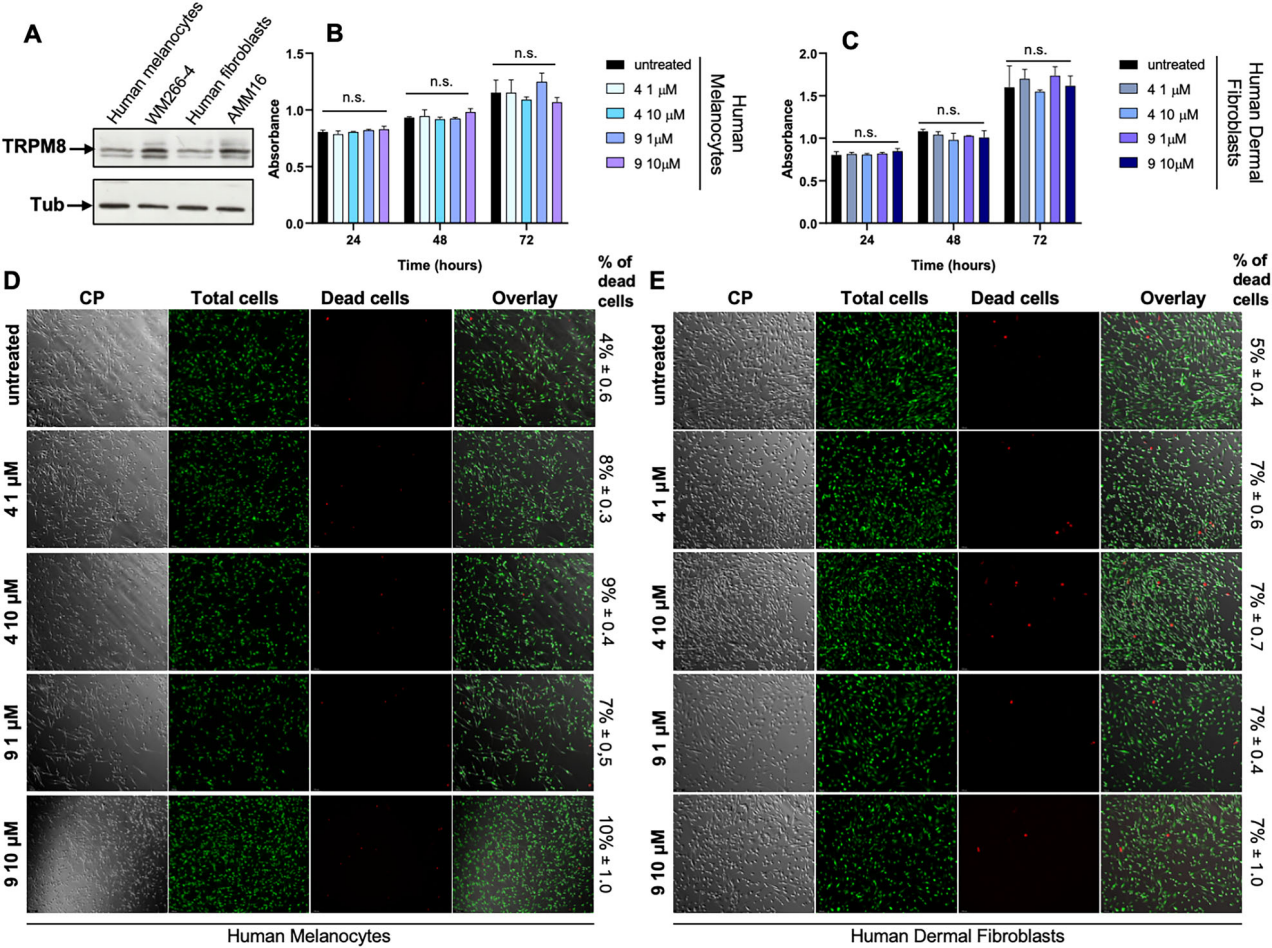
TRPM8 antagonists do not induce cytotoxicity in normal melanocytes and human dermal fibroblasts. **A** Representative Western blot showing TRPM8 protein expression in the indicated cell lines. Tubulin was used as loading control. Viability of human melanocytes (**B**) and human dermal fibroblasts (**C**) untreated or treated with compounds 4 and 9 at the concentrations indicated in the legends on the right. Absorbance values from WST-1 assays at 24, 48, and 72 h are shown. Data are presented as mean ± SD of three independent experiments. *n.s*. indicates not significant. Representative Live/Dead assay images of human melanocytes (**D**) and human dermal fibroblasts (**E**) treated for 24 h with compounds 4 and 9 (1 or 10 μM). Viable cells are shown in green (acridine orange; total cells), while dead cells are shown in red (propidium iodide; dead cells). Overlay images are shown. Scale bar, 100 μm. Quantification of cell death is displayed to the right of each overlay image. The percentage of dead cells was calculated as: (red-stained dead cells/green-stained total cells) × 100.

TRPM8 transcript levels are significantly higher in melanoma samples than in the normal skin counterparts, as assessed by analysis of The Cancer Genome Atlas (TCGA) Skin Cutaneous Melanoma dataset through the Genomic Data Commons (GDC) portal (Fig. 1A). The Western blot (WB) analysis in Figs. 1B and 2A consistently confirmed the higher levels of TRPM8 protein expression in human metastatic melanoma AMM16 and WM266-4 cell lines, as compared with non transformed NIH3T3 murine fibroblasts (Fig. 1B), human HaCaT keratinocytes (Fig. 1SA), human melanocyes and human dermal fibroblasts (Fig. 2A) used as controls. Immunofluorescence (IF) analysis showed that TRPM8 localizes at both plasma membrane and endoplasmic reticulum [21, 22] (Fig. 1C, D), and the specificity of the staining was confirmed using the secondary antibody alone, as control (Fig. 1SB, C).

**Fig. 3:**
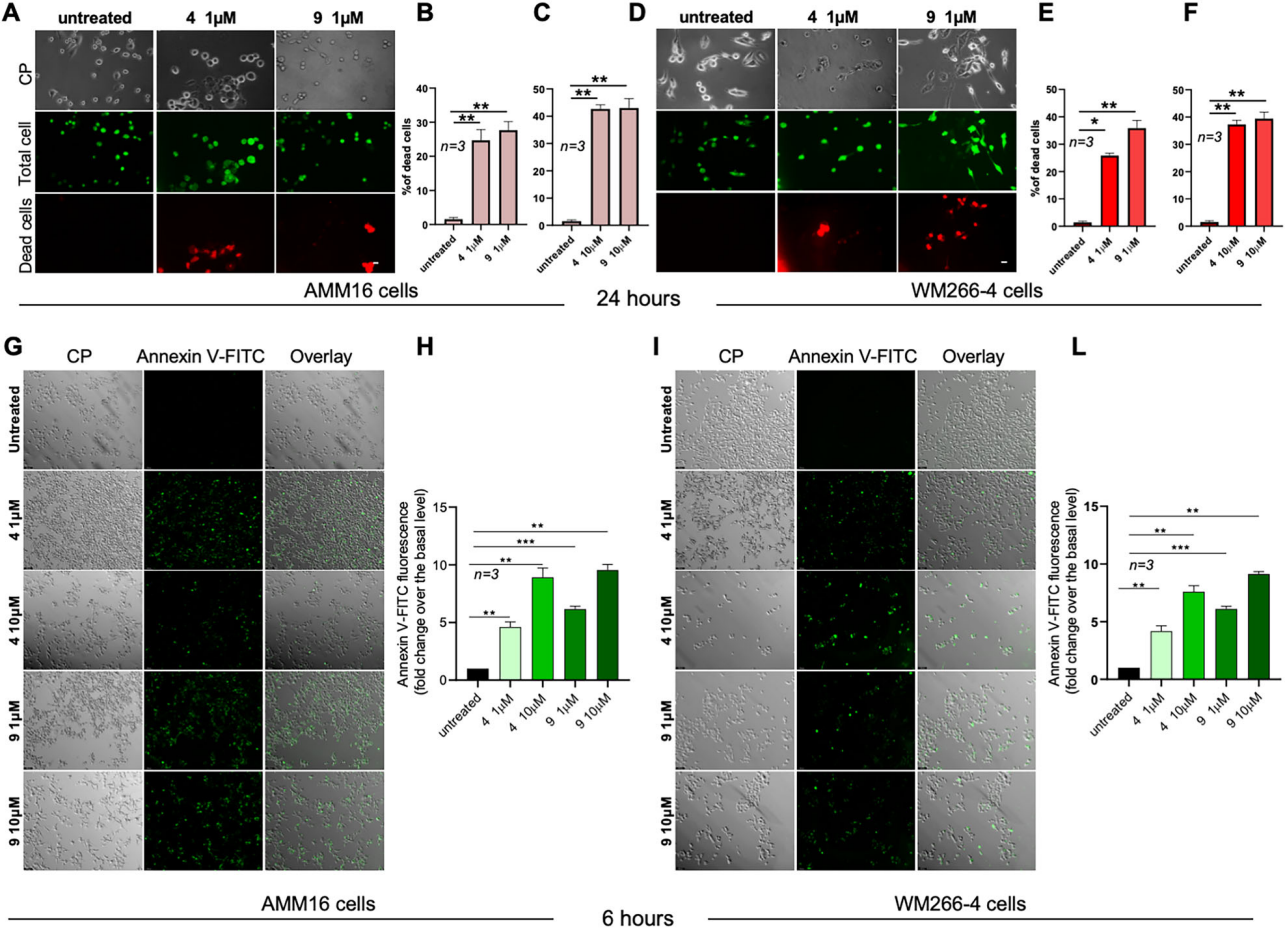
TRPM8 modulators induce melanoma apoptosis. Representative images from Live/Dead assays in AMM16 (**A**) and WM266-4 (**D**) melanoma cells treated for 24 h with TRPM8 modulators 4 and 9 (1 µM). Total cells are stained in green (acridine orange), while dead cells are labeled in red (propidium iodide). Scale bar: 100 µm. Quantification of cell death in AMM16 (**B**, **C**) and WM266-4 (**E**, **F**) cells treated with compounds 4 and 9 at 1 or 10 µM, respectively, for 24 h. The percentage of dead cells was calculated as: (dead red-stained cells/total green-stained cells) × 100. Representative images of AMM16 (**G**) and WM266-4 (**I**) melanoma cells untreated or treated for 6 h with TRPM8 modulators 4 and 9 (1 or 10 μM) and stained with Annexin V–FITC. Contrast phase (CP), Annexin V–FITC, and merged images (overlay) are shown. Scale bar, 100 μm. Quantification of Annexin V–FITC fluorescence in AMM16 (**H**) and WM266-4 (**L**) cells untreated or treated as in **G**, **I**, measured using a multiwell fluorescence reader (excitation/emission settings optimized for FITC as reported in Methods). Data represent mean ± SD from n independent experiments. Data in **B**, **C**, **E**, **F**, **H**, **L** are presented as mean ± SD from *n* = *3* independent experiments. Statistical significance was determined. ^*^p < 0.05, ^**^p < 0.01, ^***^p < 0.001.

Next, the functional relevance of TRPM8 was evaluated in melanoma AMM16 and WM266-4 cells. Treatment with structurally distinct TRPM8 modulators (compounds 3, 4, 5, 6, and 9; Fig. 2S) resulted in a dose- and time-dependent reduction in cell viability across both melanoma cell lines (Fig. 1E, F). EC₅₀ values calculated at 24, 48, and 72 h post treatment confirmed that the compounds 4 and 9 are the most potent across all the time points in both cell lines (Tables 1 and 2). While the compound 9 exhibited the highest efficacy at 72 h, the compound 4 was effective throughout the experimental time frame. The two modulators did not show cytotoxic effect in NIH3T3 fibroblasts (Fig. 3SA), HaCaT keratinocytes (Fig. 3SB), human melanocytes (Fig. 2B, D) and dermal fibroblasts (Fig. 2C, E), suggesting a selective toxicity toward melanoma cells.

**Table 1:** EC_50_ AMM16 cells.

Molecule	EC_50_ 24 h	EC_50_ 48 h	EC_50_ 72 h
3	0.4050995	0.4361841	0.6016822
5	0.6831246	1.194115	0.9967819
4	0.6096671	0.6406154	0.5696633
6	0.9148558	0.7009896	0.6091167
9	0.5002408	0.5263659	0.3899526

**Table 2:** EC_50_ WM266-4 cells.

Molecule	EC_50_ 24 h	EC_50_ 48 h	EC_50_ 72 h
3	0.8949265	0.8087331	0.6389285
5	0.9875148	0.7457261	0.6947026
4	0.8876118	0.648131	0.6849402
6	0.7477424	1.4211157	1.423566
9	0.9801319	0.831988	0.5245131

To further investigate the cellular response to TRPM8 modulation, 24 h-treatment with compound 4 or 9 at 1 (Fig. 3A, B, D, E) or 10 μM (Fig. 3C, F) significantly increased propidium iodide (PI) uptake in both AMM16 and WM266-4 cells, indicating membrane-compromised, non-viable cells (Fig. 3A–F).

To confirm that melanoma cells undergo apoptosis following TRPM8 modulation, we performed Annexin V staining in both melanoma cell lines (Fig. 3G, I). Notably, Annexin V–positive cells were already detectable after 6 h of treatment with both antagonists at 1 and 10 μM. This early apoptotic response was revealed by IF microscopy (Fig. 3G, I) and further validated by quantitative fluorescence measurements (Fig. 3H, L), demonstrating that TRPM8 inhibition rapidly initiates an apoptotic program in metastatic melanoma cells.

To further demonstrate that the cytotoxic effects of compounds 4 and 9 were specifically mediated through TRPM8, we combined loss- and gain-of-function approaches.

**Fig. 4:**
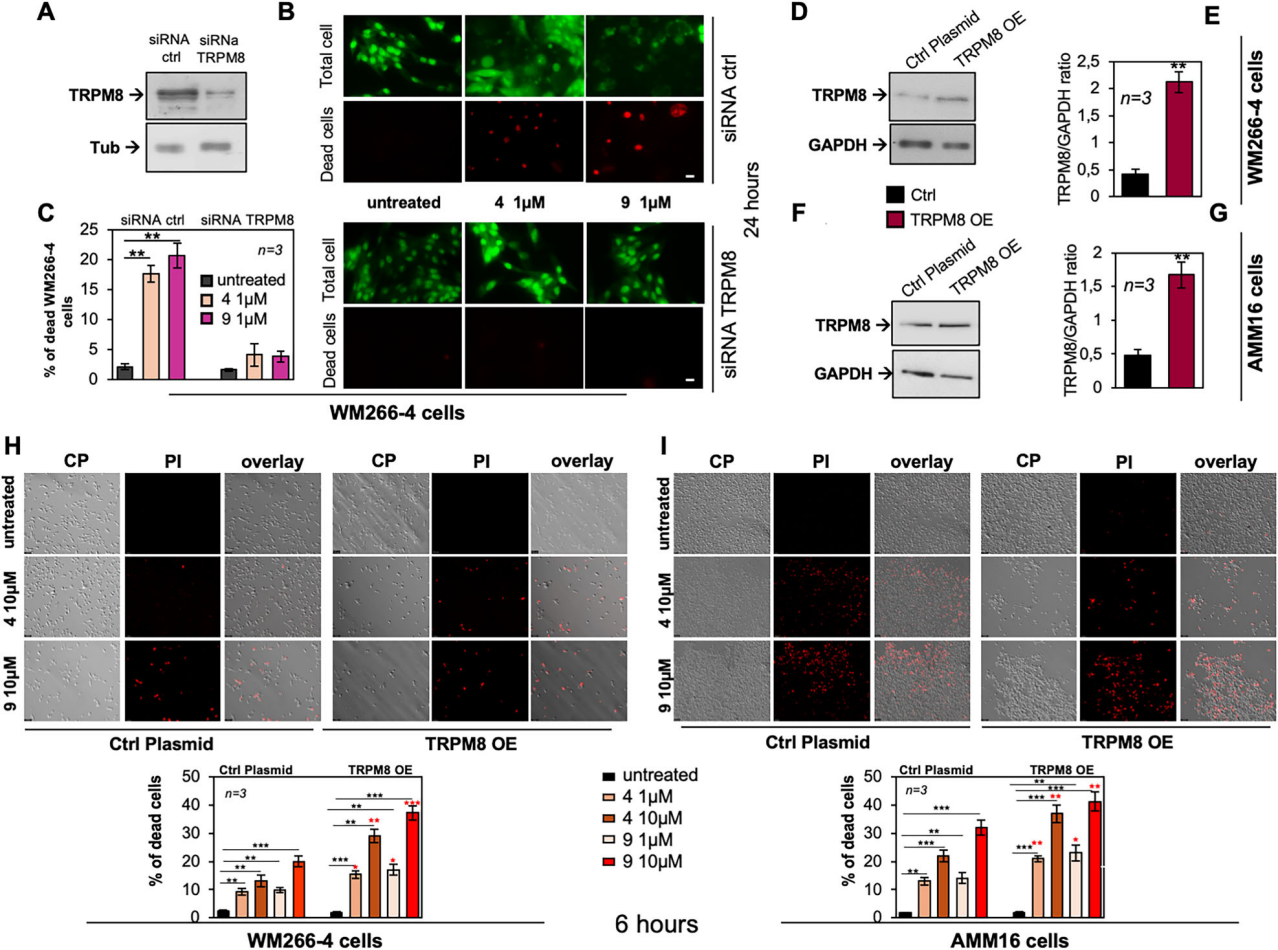
Effects of TRPM8 loss- and gain-of-function on melanoma cell death. **A**–**C** WM266-4 cells were transfected with control siRNA (siRNA ctrl) or TRPM8-targeting siRNA (siRNA TRPM8). **A** Western blot analysis was performed on total cell lysates using the indicated antibodies. **B** Cells were then left untreated or treated with compound 4 or 9 (1 μM, 24 h), and cell death was assessed by PI staining. Total cells were stained in green with acridin orange. **C** Quantification of PI-positive cells corresponding to (**B**). Representative Western blots showing transient TRPM8 overexpression (TRPM8 OE) in WM266-4 (**D**) and AMM16 (**F**) melanoma cells. **E**, **G** Densitometric analysis of TRPM8 and GAPDH protein levels, represented as TRPM8/GAPDH ratios (from three independent experiments). WM266-4 (**H**) and AMM16 (**I**) cells transfected with control plasmid (ctrl plasmid) or TRPM8 plasmid (TRPM8 OE) were left untreated or treated with compounds 4 and 9 (1 or 10 μM) for 6 h. Representative images (contrast phase, PI staining, and overlays) and corresponding quantitative graphs (below panels) are shown. Scale bar, 100 μm. In **C**, **E**, **G**, **H**, **I** Data are presented as mean ± SD from *n* independent experiments. ^*^p < 0.05, ^**^p < 0.01, ^***^p < 0.001. In **H**, **I** Red asterisks indicate statistical significance between ctrl and OE groups at the corresponding treatment conditions.

Silencing TRPM8 by siRNA robustly reduced TRPM8 protein levels (Fig. 4A) and completely abolished compound-induced cell death in WM266-4 cells (Fig. 4B, C), suggesting that TRPM8 expression is required for the cytotoxic response.

Conversely, transient TRPM8 overexpression (TRPM8 OE) in WM266-4 (Fig. 4D, E) and AMM16 cells (Fig. 4F, G) did not affect basal viability in untreated conditions (Fig. 4H, I and lower graphs). However, TRPM8 OE significantly amplified the cytotoxic effect of both antagonists, as indicated by the increased PI uptake observed in overexpressing cells (right panels in H and I) compared with control-transfected cells exposed to identical treatments (Fig. 4H, I, left panels and graphs). This sensitizing effect was evident in both cell lines and at both 1 and 10 μM concentrations, indicating that higher TRPM8 expression enhances the pro-apoptotic activity of the modulators.

Together, these complementary loss- and gain-of-function experiments demonstrate that TRPM8 expression levels critically dictate the magnitude of the cytotoxic response to compounds 4 and 9, providing strong evidence that the observed phenotype is TRPM8-dependent.

### TRPM8 modulators induce mitochondrial oxidative stress and collapse of mitochondrial membrane potential

Although TRPM8 represents a calcium-permeable ion channel [23, 24], we did not detect any significant intracellular calcium influx upon treatment with compounds 4 and 9, using fluorescent calcium imaging. Menthol, a canonical TRPM8 agonist [25], was here used as positive control (Fig. 4SA, B). Thus, our results point to a calcium-dependent cytotoxic mechanism and suggest the involvement of alternative downstream pathways.

**Fig. 5:**
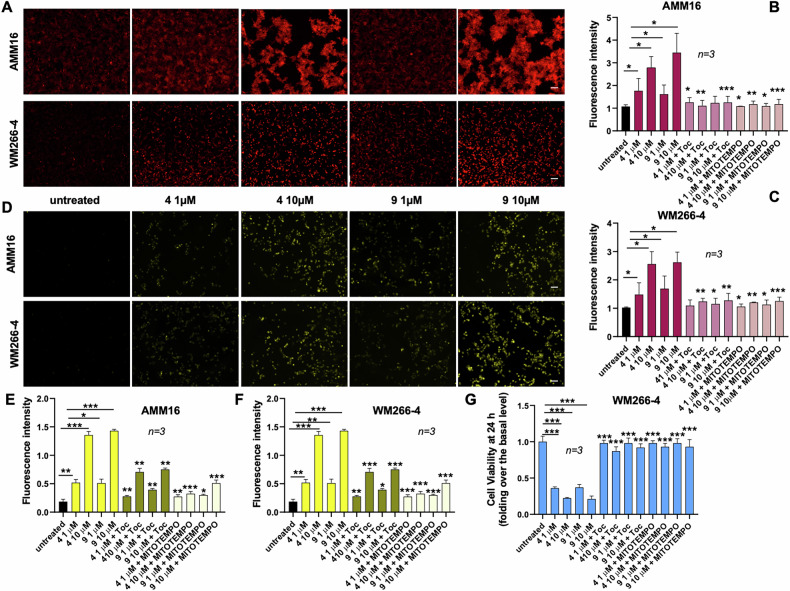
TRPM8 modulators induce ROS accumulation in melanoma cells. **A** Representative immunofluorescence images showing intracellular ROS levels in AMM16 and WM266-4 cells untreated or treated for 4 h with TRPM8 modulators 4 and 9 (1 or 10 µM). ROS were detected as reported in Methods section. Scale bar: 100 µm. **B**, **C** Quantification of intracellular ROS levels in AMM16 and WM266-4 cells treated as indicated, in the absence or presence of the antioxidants α-tocopherol (Toc) or the mitochondrial ROS scavenger MitoTEMPO. Fluorescence intensity was measured using a TECAN plate reader and expressed as fold increase over the basal levels. **D** Representative images of mitochondrial ROS levels in AMM16 and WM266-4 cells treated for 1.5 h with TRPM8 modulators 4 and 9 (1 or 10 µM). Mitochondrial ROS were detected using a mitochondria-specific ROS probe. Scale bar: 75 µm. **E**, **F** Quantification of mitochondrial ROS levels under the same treatment conditions described in **D**, with or without antioxidant co-treatment. Fluorescence intensity was measured using a TECAN plate reader and expressed as fold increase over the basal levels. **G** Viability of WM266-4 cells treated for 24 h with compounds 4 and 9 (1 or 10 µM) in the absence or presence of α-tocopherol or MitoTEMPO. Viability is expressed as fold change relative to untreated controls. Data in **B**, **C**, **E**–**G** are shown as mean ± standard deviation (SD) from *n* = 3 independent experiments. Statistical analysis was performed. ^*^p < 0.05, ^**^p < 0.01, ^***^p < 0.001 versus the corresponding untreated control.

As the ion channels regulate cellular redox balance [26], we next investigated whether TRPM8 modulators perturb oxidative homeostasis. DCFDA-based assays, which detects a broad range of reactive oxygen species (ROS), including hydrogen peroxide (H₂O₂), hydroxyl radicals (•OH), and peroxynitrite (ONOO⁻) [27], revealed a robust and dose-dependent increase in total intracellular ROS levels upon 4h-treatment with compounds 4 and 9 in both the cell lines (Fig. 5A–C). Addition of α-tocopherol, a lipid-soluble antioxidant that neutralizes lipid peroxyl radicals and prevents propagation of the oxidative chain [28], reversed this effect (Fig. 5B, C). MitoTEMPO, a mitochondria-targeted superoxide dismutase mimetic, which selectively scavenges mitochondrial superoxide (O₂•⁻), gave similar results (Fig. 5B, C). Thus, ROS generation involves both cytoplasmic and mitochondrial compartments.

Contribution of mitochondria to the observed phenotype, was further assessed by using the mitochondrial superoxide indicator, MitoSOX. Such fluorogenic dye selectively targets mitochondria and specifically reacts with superoxide anions, but not with other ROS, such as H₂O₂ or •OH. As such, it can be considered a reliable marker of mitochondrial oxidative stress. MitoSOX assay further confirmed an early (1.5 h) and significant accumulation of mitochondrial superoxide on TRPM8 modulation in both the cell lines (Fig. 5D–F). In our settings, mitochondrial superoxide is the key oxidative species driving cytotoxicity, as MitoTEMPO was more effective than α-tocopherol in quenching mitochondrial ROS (Fig. 5E, F).

Cell viability assays consistently show that α-tocopherol and MitoTEMPO both restore the reduction in viability induced by compounds 4 and 9. As the effect was evident at both 24 h (Fig. 5G) and 72 h (Fig. 4SC), we concluded that the redox imbalance is not a merely transient response, but a key driver of cytotoxicity.

**Fig. 6:**
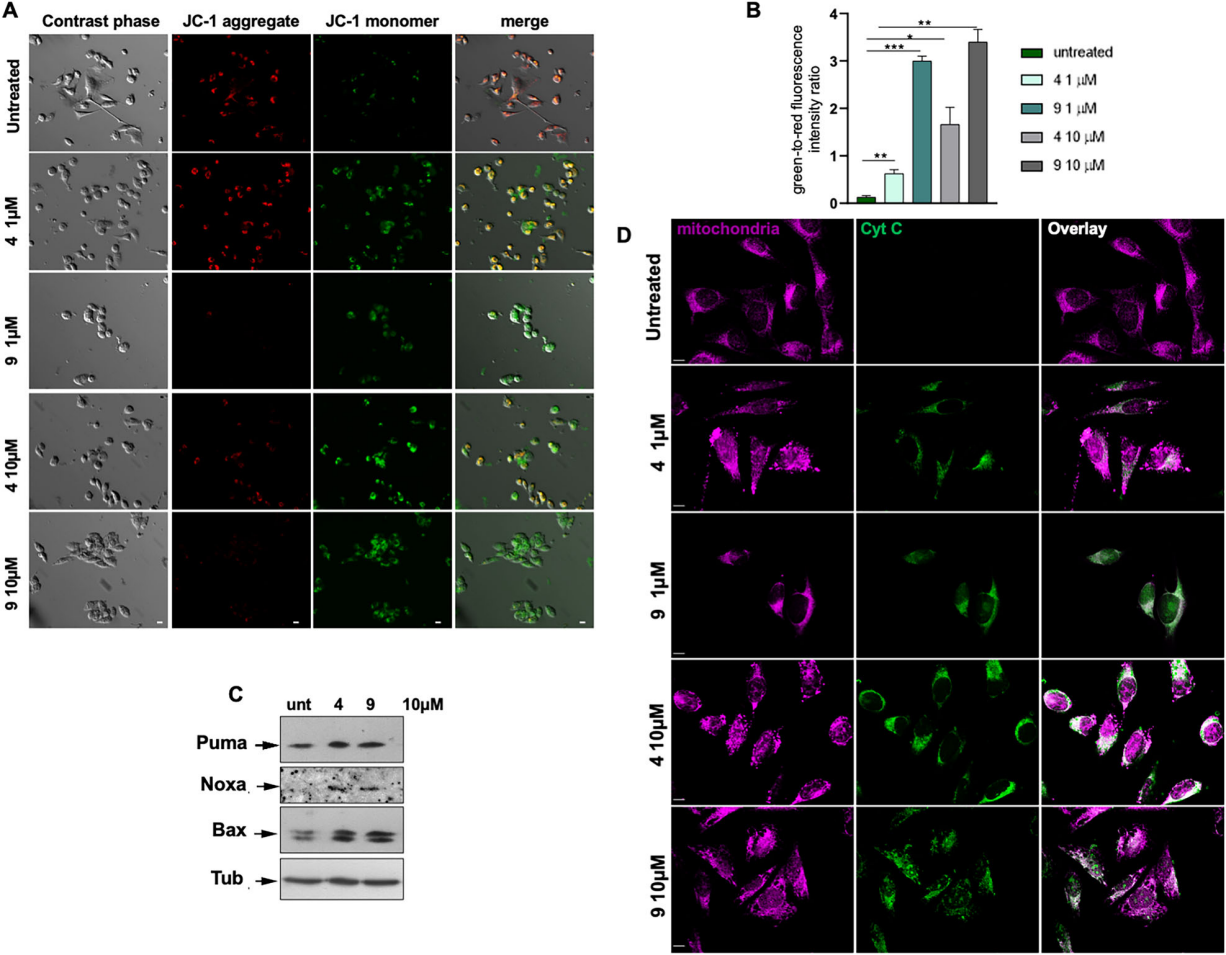
TRPM8 modulators induce mitochondrial depolarization, upregulation of pro-apoptotic proteins, and cytochrome c release in melanoma cells. **A**, **D** WM266-4 cells were treated with TRPM8 modulators, as indicated, for 6 h. **A** Representative phase contrast and fluorescence microscopy images. JC-1 staining reveals mitochondrial depolarization following TRPM8 inhibition, as indicated by a fluorescence shift from red (JC-1 aggregates in polarized mitochondria) to green (JC-1 monomers in depolarized mitochondria). Scale bar, 10 μm. **B** Quantification of red-to-green fluorescence ratio, calculated as described in the Methods section. Data are presented as mean ± SD from three independent experiments (*n* = *3*). ^*^p < 0.05, ^**^p < 0.01, ^***^p < 0.001 vs. the corresponding untreated control. **C** Western blot analysis of cell lysates obtained after 24 h of treatment, using the indicated antibodies. **D** Representative confocal microscopy images showing cytochrome c (green) localization and mitochondria (violet) in melanoma cells. Fluorescence signals were acquired using identical exposure settings. Scale bar, 10 μm.

JC-1 staining demonstrated, indeed, a dose-dependent loss of mitochondrial membrane potential (Δψm) in melanoma cells treated with compounds 4 and 9, as evidenced by the increase in green fluorescence signal. The fluorescence shift (from green to red) likely reflects the inability of JC-1 to accumulate in depolarized mitochondria, which results in its monomeric cytoplasmic localization (Fig. 6A). Quantitative analysis of these data is presented in Fig. 6B as the green-to-red fluorescence intensity ratio. The Δψm collapse is a well-established hallmark of mitochondrial outer membrane permeabilization (MOMP) and precedes apoptotic commitment [29].

### Mitochondrial oxidative stress activates intrinsic apoptosis via p53 and BH3-only proteins

Contribution of TRPM8 modulators to the intrinsic apoptotic pathway activation was next assessed by analysis of core pro-apoptotic proteins [30]. TRPM8 modulators upregulated the BH3-only proteins Noxa and Puma, as well as Bax, a key initiators of MOMP (Fig. 6C). The coordinated increase in pro-apoptotic signals would enable a mitochondrial priming state, which irreversibly commits cells to apoptosis.

Consistent with Bax-mediated MOMP, TRPM8 modulators induce a dose-dependent cytosolic translocation of cytochrome c (Fig. 6D), confirming engagement of the intrinsic apoptotic cascade. A marked alteration in mitochondrial morphology was simultaneously seen, as mitochondria displayed an intact, filamentous network in control cells, while fragmented and discontinuous mitochondrial structures, indicative of early mitochondrial injury, were detectable in treated cells (Fig. 6D). MitoTEMPO, which prevents cytochrome c release and morphological damage (Fig. 4SD), restored the normal phenotype. These findings strongly implicate mitochondrial superoxide, as the critical upstream effector of TRPM8-induced MOMP. The specificity of cytochrome c immunostaining was validated using the FITC-conjugated secondary antibody alone, as control (Fig. 4SE).

### ROS triggers DNA damage and p53-dependent apoptotic programming

**Fig. 7:**
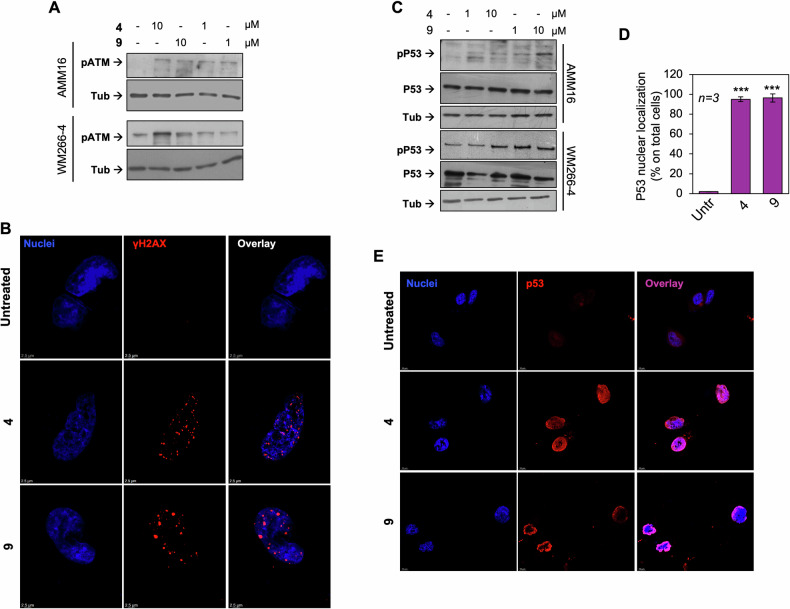
TRPM8 modulation induces DNA damage response and p53 activation in melanoma cells. **A** Western blot analysis of cell lysates collected after 7 h of treatment with TRPM8 modulators, using the indicated antibodies. **B** Immunofluorescence analysis of cells left untreated or treated with TRPM8 modulators (10 μM, 7 h), stained for γH2AX (red punctate nuclear foci) and nuclei (blue). Overlayed images are shown. Scale bar, 2.5 μm. **C** Western blot analysis of cell lysates using the indicated antibodies. **D** p53 localization was expressed as percentage of cells showing a nuclear localization. Means and SDs are shown; n represents the number of experiments. ^***^P < 0.001 for the indicated experimental points versus the corresponding untreated control cells. **E** Representative Immunofluorescence images of WM266-4 cells left untreated or treated with TRPM8 modulators (10 μM, 7 h), stained for p53 (red) and nuclei (blue) and quantified in **D**. Overlayed images are shown. Scale bar, 10 μm. In **B**, **E** fluorescence signals were acquired using identical exposure settings.

Oxidative stress often converges on DNA damage pathways [31]. In melanoma cells treated with TRPM8 modulators, we observed phosphorylation of ATM, a key sensor of DNA double-strand breaks (Fig. 7A). This effect was prevented by both α-tocopherol and MitoTEMPO (Fig. 5SA, B). These findings suggest that oxidative stress induced by TRPM8 modulators affect multiple cellular compartments, with nuclear stress responses arising from more generalized intracellular ROS. ATM activation was followed by a robust phosphorylation of histone H2AX at Ser139 (γ-H2AX), with the formation of characteristic punctate nuclear foci, indicative of localized DNA damage (Fig. 7B). The specificity of the staining was confirmed using Texas Red-conjugated secondary antibody alone, as control (Fig. 5SC). Phosphorylation of Ser15-p53 was subsequently detected (Fig. 7C), indicating the initiation of a canonical DNA damage response. Notably, this phosphorylation event was not observed in non-tumorigenic NIH3T3 fibroblasts or HaCaT keratinocytes (Fig. 5SD), further supporting the selective activation of stress response pathways in melanoma cells upon TRPM8 modulation. TRPM8 modulators also induced a pronounced nuclear accumulation of p53 (quantified in the graph in Fig. 7D), shifting from a predominantly cytoplasmic localization in untreated cells to a strong nuclear enrichment in treated cells. This redistribution was confirmed by IF analysis (Fig. 7E), with the specificity of the immunostaining shown in Fig. 5SE.

**Fig. 8:**
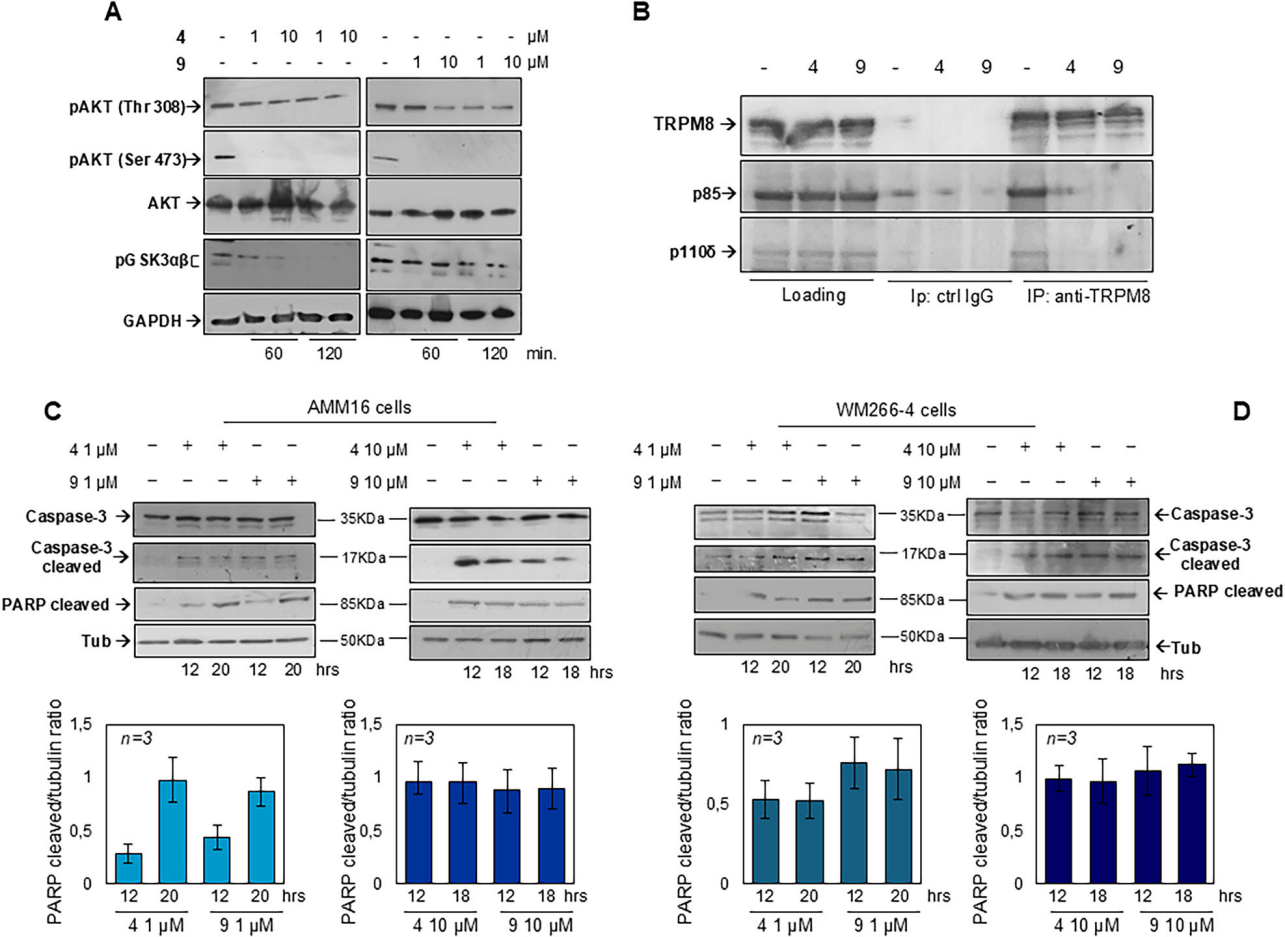
TRPM8 modulators impair PI3K/AKT axis and induce apoptotic cell death. **A** Western blot analysis of WM266-4 cell lysates collected after 60 or 120 min of treatment with TRPM8 modulators, using the indicated antibodies. **B** WM266-4 cells were untreated (-) or treated for 120 min with TRPM8 antagonists (4 and 9, used at 10 µM). Lysate proteins were immune-precipitated using the anti-TRPM8 (anti-TRPM8) or control (ctrl IgG) antibodies. WB analysis using antibodies against the indicated proteins was done to reveal co-immunoprecipitated proteins. Western blot analysis of cleaved caspase-3 and cleaved PARP in AMM16 (**C**) and WM266-4 (**D**) cells treated with TRPM8 modulators at the indicated concentrations and hours. The α-tubulin was used as loading control. Graphs in the lower part of the figure represent the densitometric analysis of the cleaved PARP/tubulin ratio obtained in three different experiments (*n* = *3*).

Interestingly, the cytotoxic effect of TRPM8 modulators results from both the activation of pro-apoptotic pathways and inhibition of key pro-survival signaling pathways. Infact, a dose- and time-dependent decrease in the phosphorylation of AKT and GSK3α/β was observed (Fig. 8A). Both Thr308 and Ser473 residues of AKT were affected, indicating a comprehensive inhibition of its activation (Fig. 8A). Notably, co-treatment with α-tocopherol or MitoTEMPO restored AKT phosphorylation (Fig. 5SF), confirming the ROS dependence of this effect. Importantly, TRPM8 modulators failed to suppress AKT phosphorylation in non-tumorigenic NIH3T3 and HaCaT cells (Fig. 5SG), highlighting the selective vulnerability of melanoma cells to TRPM8-targeted disruption of survival signaling.

To investigate the mechanism by which TRPM8 antagonists regulate the AKT axis, we examined whether TRPM8 physically associates with PI3K. Co-immunoprecipitation assay revealed a basal interaction between TRPM8 and both the p85 and p110δ subunits of PI3K, which was markedly reduced upon treatment with TRPM8 modulators within 2 h, suggesting a direct role for TRPM8 in stabilizing PI3K/AKT signaling complexes at the plasma membrane. As shown in Fig. 8B (left panel), equal amounts of TRPM8, p85, and p110δ were detected in the input fractions, confirming consistent protein loading. The right panel illustrates that treatment with 10 μM TRPM8 modulators disrupted the TRPM8–PI3K complex. No significant signal was detected in samples immunoprecipitated with control IgG (middle panel) if compared with the Co-IP, confirming the specificity of the assay. Thus, our results posit ROS as upstream mediators of multiple interconnected signaling cascades, including mitochondrial dysfunction, DNA damage response, and survival pathway suppression, that collectively contribute to melanoma cell death upon TRPM8 modulation. Importantly, ROS scavenging attenuated TRPM8-induced AKT inhibition, supporting that oxidative stress may lie upstream of AKT deactivation. Nonetheless, TRPM8 was also observed to associate with the PI3K subunits p85 and p110, implying a possible direct influence on the PI3K/AKT axis independent of ROS.

Given that MOMP and cytochrome c release are upstream of caspase activation, we next analyzed the execution phase of apoptosis. As shown in Fig. 8C, D, and relative densitometric analysis (lower graphs), TRPM8 modulators treatment resulted in robust caspase-3 activation and PARP cleavage, hallmark events of the intrinsic apoptotic pathway [32]. These findings consolidate a model in which compounds 4 and 9 initiate a ROS-dependent, p53-driven mitochondrial apoptosis cascade involving BH3-only proteins and the caspase machinery.

### TRPM8 modulation enhances NK cell-mediated immunosurveillance via ULBP1–NKG2D axis

**Fig. 9:**
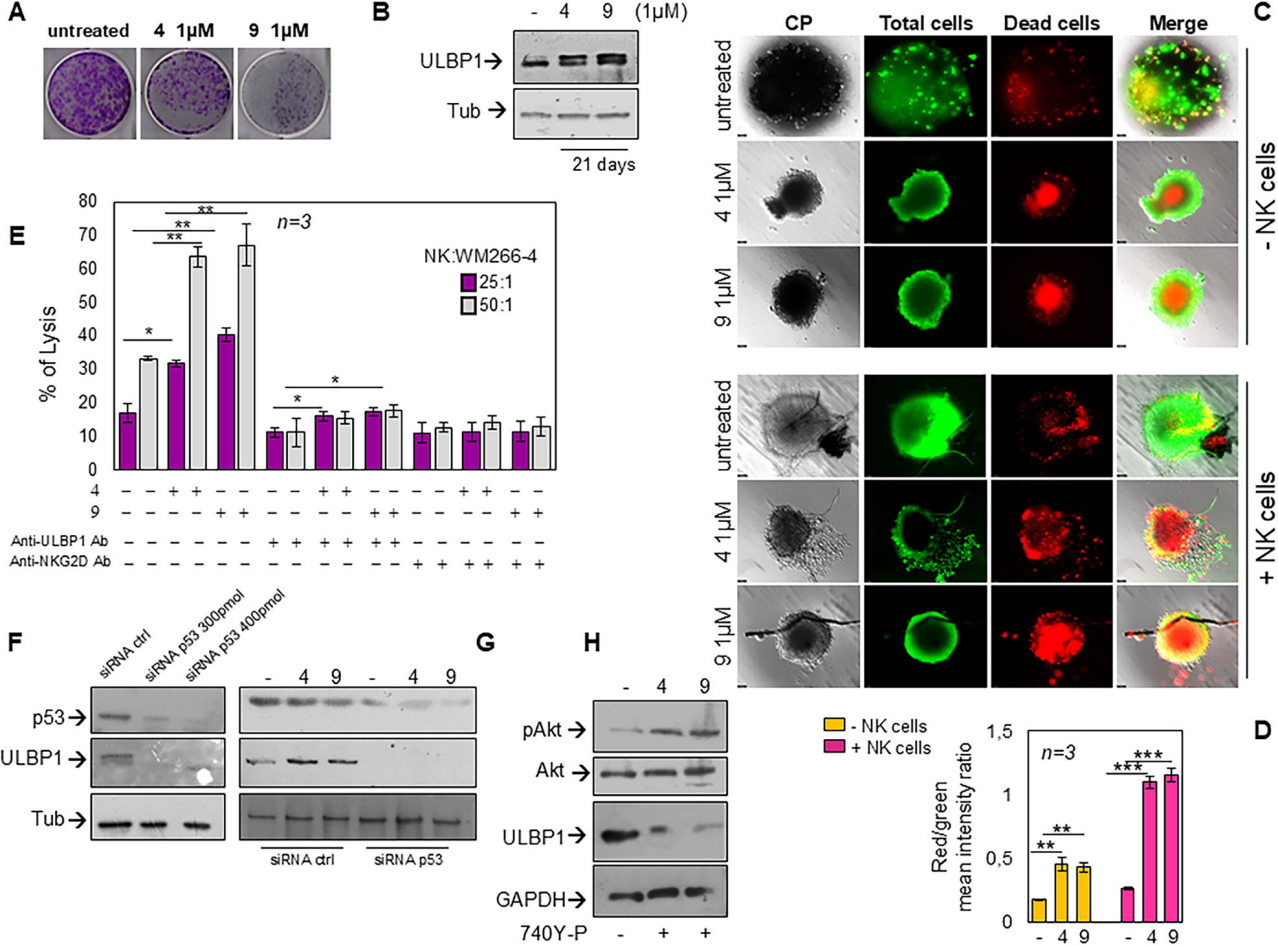
TRPM8 modulators reduce clonogenic potential, upregulate ULBP1, and sensitize melanoma cells to NK cell-mediated cytotoxicity. **A** Representative images of crystal violet-stained colonies, derived from WM266-4 cells, after 21-day treatment with TRPM8 modulators. **B** Western blot analysis showing ULBP1 expression in WM266-4 cells after treatment with TRPM8 modulators. The α-tubulin was used as a loading control. **C** WM266-4 derived spheroids treated for 21 days as indicated, in absence (upper panel; -NK cells) or presence (lower panel; + NK cells) of NK cells. Images are representative of three different experiments. Bar, 100 µm. **D** The graph represents the dead cells/total cells. Values of dead (red stained cells) and total cells (green stained cells) were analyzed using NIH Image J. They derive from red fluorescence mean/green fluorescence mean intensity and are expressed as mean ± SD of 3 different experiments (*n* = 3); **p < 0.01; *** p < 0.001. **E** NK cell cytotoxicity assay. WM266-4 cells pre-treated with TRPM8 modulators were co-cultured with primary NK cells at the indicated effector:target (E:T) ratios. Where indicated, neutralizing antibodies against ULBP1 or NKG2D were added 1 h before co-culture to melanoma cells or NK, respectively. Data are presented as percentage of lysis.^*^p < 0.05; ^**^p < 0.01. **F** WM266-4 cells were transfected with control siRNA (siRNA ctrl) or TP53-targeting siRNA (siRNA p53) at two different concentrations (300 pmol and 400 pmol, respectively). After 4 days, cells were collected, lysed, and Western blot analysis was performed on cell lysates using the indicated antibodies. α-Tubulin was used as a loading control. **G** WM2664 cells transfected with control siRNA (siRNA ctrl) or TP53-targeting siRNA (siRNA p53; 300 pmol) were unstimulated or stimulated with compounds 4 and 9 (at 1 μM) for 4 days and then collected and lysed. Western blot analysis was performed using the indicated antibodies. α-Tubulin was used as a loading control. **H** Phosphorylated AKT (Ser473) levels in melanoma cells treated with the PI3K agonist, 740 Y-P, in absence or presence of TRPM8 modulators (used at 1 μM) for 4 days, analyzed by Western blot. GAPDH was used as a loading control.

In addition to promoting intrinsic apoptosis, TRPM8 modulation enhances the immune susceptibility of melanoma cells. Long-term exposure (21 days) to 10 μM TRPM8 modulators completely abrogated colony formation (Fig. 5SH). When the concentration of modulators was reduced to 1 μM, a ~50% decrease in colony number was still observed (Figs. 9A and 5SI).

To investigate potential mechanisms of immune-mediated clearance in cells that escape apoptosis, we analyzed the expression of stress-induced ligands for the activating NK cell receptor NKG2D [33]. Interestingly, ULBP1 expression was markedly upregulated after only 3 days of treatment with low-dose (1 μM) TRPM8 modulators (Fig. 5SL), and further increased following prolonged exposure (21 days; Fig. 9B). In contrast, other NKG2D ligands such as ULBP2, ULBP4, and MICA/B were not modulated (data not shown), indicating a selective upregulation of ULBP1. This suggests that even melanoma cells that resist apoptosis may undergo stress-induced changes that enhance their immunogenicity. The selective induction of ULBP1 may sensitize these residual tumor cells to NK cell–mediated cytotoxicity, providing an alternative mechanism for tumor control. These findings might support the rationale for combining TRPM8-targeted therapies with NK-based immunotherapy to overcome resistance and achieve more durable anti-tumor responses.

Since ULBP1 is a known ligand of the activating NK cell receptor NKG2D [34], we next assessed the functional consequences of its upregulation. We first confirmed that TRPM8 modulators did not affect NK cell viability (Fig. 6SA), and TRPM8 protein was undetectable by Western blot in NK cells (Fig. 6SB). Moreover, transcriptomic data revealed a scant TRPM8 mRNA expression in NK cells (Figs. 6SC and 7SA), supporting a tumor-selective effect of TRPM8 targeting.

We then employed WM266-4 melanoma-derived spheroids that were either left untreated or treated with 1 μM TRPM8 modulators for 21 days. First, we observed that treatment with the compounds induced a marked reduction in spheroid area (Fig. 5SM), together with an increase in red fluorescence, indicative of cell death (Fig. 9C, upper panel). When NK cells were added for 8 h in co-culture with WM266-4-derived spheroids we detected an additional increase in melanoma cell death through immunofluorescence microscopy (Fig. 9C, lower panel). Although we showed that TRPM8 antagonists can induce cell death in the absence of NK cells, the presence of NK cells further enhanced melanoma cytotoxicity in both untreated and treated organoids, with the strongest effect observed following antagonists treatment. Notably, NK-mediated cytotoxicity extended to the peripheral layers of the organoids, whereas in the absence of NK cells cell death remained predominantly confined to the central core (Fig. 9C).

Quantification of mean red fluorescence intensity demonstrated a significant increase upon treatment with TRPM8 modulators, which was further elevated after NK cell co-culture. Statistical comparisons between each treatment condition, with or without NK cells, confirmed significant differences (Fig. 9D). Overall, these results indicate that TRPM8 modulation enhances the susceptibility of melanoma spheroids to NK-mediated cytotoxicity.

To functionally validate these findings, melanoma cells pre-treated with 1 μM TRPM8 modulators for 21 days were co-cultured with NK cells for 8 h in 2D, and cytotoxicity was quantified. In this setting, melanoma cells treated with TRPM8 antagonists became significantly more sensitive to NK cell-mediated killing. Co-culture in the presence of neutralizing antibodies anti-ULBP1 or -NKG2D, markedly reduced this effect (Fig. 9E). Notably, the greater inhibitory effect observed with anti-NKG2D antibodies supports the key role of the ULBP1–NKG2D axis in this process.

To investigate the molecular mechanisms underlying ULBP1 induction, we next silenced p53 (Fig. 9F). This transcription factor has been shown to be involved in the regulation of NKG2D ligands in human cells, and in particular, functional p53-responsive elements have been identified within the first introns of the human ULBP1 and ULBP2 genes [35]. Furthermore, p53 has been shown to bind directly to the ULBP1 and ULBP2 loci, suggesting that it can act as a transcriptional amplifier under stress conditions [36, 37]. Consistent with these data, we silenced p53 to directly assess its contribution to ULBP1 regulation. p53 knockdown in melanoma cells led to a marked reduction in ULBP1 expression under basal conditions (Fig. 9F) as well as following treatment with TRPM8 modulators (Fig. 9G), indicating that in our settings p53 is required for both constitutive and drug-induced ULBP1 expression.

Additionally, activation of AKT via 740Y-P, which counteracts the TRPM8 modulator-induced AKT dephosphorylation, markedly suppressed ULBP1 upregulation (Fig. 9H). These findings identify a TRPM8–AKT and/or TRPM8–p53 signaling axis as central regulators of both cell death and immunogenic potential in melanoma cells. This is in line with previous evidence indicating that AKT negatively regulates p53 function through MDM2-mediated degradation [38] and that p53 directly controls ULBP1 transcription [36, 37, 39]. Moreover, ULBP1 upregulation has been associated with enhanced susceptibility of tumor cells to NKG2D-mediated cytotoxicity, thus linking stress signaling to innate immune recognition [33, 40, 41].

Taken together, our results indicate that TRPM8 modulators can trigger ROS- and p53-dependent mitochondrial apoptosis and simultaneously enhance NK cell-mediated immune clearance via the ULBP1–NKG2D pathway in vitro. This dual mechanism offers a promising therapeutic strategy that combines direct cytotoxicity with increased tumor immunogenicity.

## Discussion

This study uncovers thea new vulnerability of metastatic melanoma to pharmacological modulation of the TRPM8 ion channel, revealing a calcium-independent, mitochondria-centric mechanism of apoptosis. We show that two selective TRPM8 antagonists, compounds 4 and 9, induce robust cytotoxicity in melanoma cells while sparing melanocytes, fibroblasts, and keratinocytes, thereby delineating a promising therapeutic window.

TRPM8 is overexpressed in melanoma [42], as confirmed through analysis of TCGA datasets and Western blotting of metastatic cell lines. Its localization at the plasma membrane and endoplasmic reticulum [43] suggests that TRPM8 may act as a stress-sensing hub, supporting tumor survival.

As expected, the canonical agonist menthol triggers rapid Ca²⁺ influx [44], whereas compounds 4 and 9 do not activate Ca²⁺ signaling, instead promoting a ROS-driven intrinsic apoptotic program (see graphical abstract).

Mechanistically, mitochondrial ROS accumulation emerges as the earliest detectable event and precedes mitochondrial depolarization, upregulation of BH3-only proteins (Puma, Noxa), Bax activation, and cytochrome c release [45, 46].

Both α-tocopherol and MitoTEMPO blunt ROS levels, preserve mitochondrial integrity, and prevent cytochrome c release, underscoring the centrality of mitochondrial oxidative stress in apoptotic execution. These findings support the view that mitochondrial priming [37, 38] is a determinant of apoptotic sensitivity in melanoma [47–49] and other malignancies [50].

TRPM8 antagonists also suppress AKT phosphorylation at both Ser473 and Thr308, followed by inhibition of the downstream target GSK3 α/β [51]. This effect is fully prevented by antioxidants, indicating that oxidative stress impairs PI3K–AKT survival signaling [52]. Co-IP experiments show that TRPM8 interacts with the p85 regulatory and p110δ catalytic subunits of PI3K under basal conditions, and that antagonists disrupt this association. Together, these findings outline a dual mechanism of AKT inactivation: redox-dependent suppression of upstream signaling and physical dissociation of PI3K from TRPM8.

Multiple sources may contribute to the observed ROS burst. Perturbation of ΔΨm may promote electron leakage from Complexes I and III [53, 54]. Second, TRPM8 modulation might enhance mitochondrial oxidative metabolism in a dysfunctional manner or activate alternative ROS sources, including NADPH oxidases [6] or lipid peroxidation-associated pathways [55–59]. Again, partial uncoupling of oxidative phosphorylation may further amplify ROS production upstream of apoptosis [60]. Future studies employing electron transport chain inhibitors, NADPH oxidase blockers, and uncoupling agents will help clarify these mechanisms.

A key finding of this study is that TRPM8 modulation exerts a dual anti-tumor effect, simultaneously inducing apoptosis and enhancing immune susceptibility. Long-term, sublethal exposure to compounds 4 and 9 selectively upregulated ULBP1, an NKG2D ligand [33], without affecting other ligands.

Functionally, TRPM8-treated melanoma cells and 3D spheroids became more vulnerable to NK-cell cytotoxicity in a strictly ULBP1–NKG2D–dependent manner, as blocking either component abolished killing.

Mechanistically, we identify two convergent pathways that regulate ULBP1. Activation of AKT using 740Y-P [61] suppresses ULBP1 induction, consistent with the negative regulation of NKG2D ligand expression by AKT [61]. In parallel, p53 knockdown abrogates both basal and TRPM8-induced ULBP1 expression, in agreement with literature showing p53-mediated activation of ULBP1 and ULBP2 [36, 37, 39]. Thus, TRPM8 inhibition enhances tumor immunogenicity through combined AKT inhibition and p53-dependent transcriptional activation. Importantly, NK cell viability remained unaffected, supporting the selectivity of TRPM8 modulators.

A critical aspect of our study is the demonstration that the pro-apoptotic activity of compounds 4 and 9 is strictly TRPM8-dependent. By combining loss- and gain-of-function approaches, we show that TRPM8 expression levels directly dictate the magnitude of the cytotoxic response. Silencing TRPM8 completely abolished compound-induced apoptosis. This result strongly argues against an off-target mechanism, because abrogation of the target should not prevent cytotoxicity if the compounds were acting independently of TRPM8.

Conversely, transient TRPM8 overexpression significantly enhanced the apoptotic response to both antagonists in two metastatic melanoma lines. TRPM8-overexpressing cells exhibited markedly higher PI uptake and loss of viability compared with control-transfected cells treated under identical conditions. Importantly, TRPM8 overexpression alone did not alter basal survival, indicating that TRPM8 abundance does not intrinsically induce toxicity but rather potentiates the response to drugs. Collectively, these complementary manipulations demonstrate a dose-dependent relationship between TRPM8 levels and susceptibility to apoptosis, providing compelling evidence that the cytotoxic activity of compounds 4 and 9 is mediated through TRPM8 itself.

This genetic validation strengthens the mechanistic framework proposed here and rules out the possibility that the mitochondrial oxidative stress observed upon treatment arises from TRPM8-independent off-target activities.

TRPM8 has historically been studied in nociception [62, 63], thermosensation [64], and prostate cancer [8, 11, 65, 66], with limited evidence in melanoma. Previous studies using menthol in G-361 and A-375 melanoma cells described a calcium-dependent cytotoxicity [20, 67], while our data introduce a paradigm shift: TRPM8 inhibition can drive lethal mitochondrial oxidative stress independently of Ca²⁺ influx, broadening the therapeutic potential of TRPM8 modulation.

Recent transcriptomic analyzes have identified TRPM8 among a quartet of hub genes (NOX4, NTN4, PROX1) associated with cellular senescence and immune-favorable prognostic signatures in melanoma [68]. Other studies implicate TRPM8 in DNA damage responses, with its inhibition dampening γH2AX and ATM/p53 signaling following irradiation [18], suggesting broader roles in genome surveillance and stress adaptation.

The selective induction of mitochondrial ROS by TRPM8 antagonists offers opportunities for exploiting redox vulnerabilities in melanoma. Given the high metabolic plasticity and robust antioxidant defenses of melanoma [69, 70], which contribute to resistance to targeted and immune-checkpoint therapies [71, 72], overwhelming the mitochondrial redox control may represent a strategy to bypass resistance. Notably, our data obtained in 3D models confirm that TRPM8 modulators retain activity even in ECM-rich, drug-tolerant microenvironments. Additionally, TRPM8 inhibition did not activate NF-κB, a central hub of prosurvival and inflammatory signaling [46, 73], as evidenced by unchanged p65 translocation and IKKα/β phosphorylation (Fig. S8), countering a major route of therapy resistance [46, 59, 60, 73].

From a translational standpoint, these results reposition TRPM8 as more than a diagnostic marker: it emerges as a mitochondrial gatekeeper and druggable redox effector at the intersection of stress signaling [74], metabolism [75], and immune escape [76].

Interestingly, both TRPM8 agonists and antagonists have been reported to exert anti-tumor functions, though likely through distinct mechanisms. Agonists induce Ca²⁺ flux followed by desensitization [77]. This phenomenon has been observed for classical TRPM8 agonists such as menthol [78], icilin [79], and WS-12 [80], which induce a rapid Ca²⁺ influx followed by attenuation of channel activity and downstream signaling. Antagonists such as BCTC or THIQ-derived compounds [14, 81, 82] bypass Ca²⁺ signaling and directly trigger mitochondrial apoptosis, as observed in our data. These distinctions underscore the need for precise pharmacological characterization of TRPM8 ligands [75, 83–88].

In conclusion, we propose that TRPM8 inhibition disrupts mitochondrial and redox homeostasis, triggers apoptosis, and enhances NK-mediated immunogenicity in melanoma. Future studies will employ proteomic and metabolomic approaches to map the mitochondrial interface of TRPM8 and evaluate the therapeutic promise of combining TRPM8 antagonists with NK-based immunotherapies in vivo.

## Materials and methods

### Cell lines and culture conditions

The human metastatic melanoma cell lines AMM16 [89] and WM266-4 (ATCC® CRL-1676™) were cultured in RPMI-1640 medium (Gibco, Thermo Fisher Scientific, Waltham, MA, USA) supplemented with 10% fetal bovine serum (FBS; Gibco), 2 mM L-glutamine, and 1% penicillin-streptomycin (Gibco).

The human epidermal Melanocytes (P10853, Innoprot, Bizkaia, Spain) were cultured in Melanocyte Medium (MeIM; Innoprot) supplemented with 5% FBS (Gibco), 1% Melanocyte Growth Supplement (MelSG; Innoprot) and 1% penicillin-streptomycin (Gibco).

The Immortalized Human Dermal Fibroblasts (P10858-IM, Innoprot) were cultured in Fibroblast Medium-2 (FM-2; Innoprot) supplemented with 5% FBS (Gibco), 1% Fibroblast Growth Supplement-2 (FGS-2; Innoprot) and 1% penicillin-streptomycin (Gibco).

The HaCaT human keratinocytes (ATCC) were cultured in DMEM supplemented with 10% FBS, 2 mM L-glutamine, 100 μ/mL streptomycin, and 0.1 mM non-essential amino acids. NIH3T3 murine fibroblasts (ATCC® CRL-1658™) were cultured as previously described [90]. Primary human NK cells (70036, Voden, Meda, Italy) were cultured in ImmunoCult NK Cell Base Medium (Stemcell Technologies, Basel, Switzerland).

Cells were maintained at 37 °C in a humidified incubator with 5% CO₂ and routinely tested for mycoplasma contamination.

### Pharmacological compounds and patch-clamp experiments

A panel of TRPM8 modulators (compounds 3, 4, 5, 6, and 9) was synthesized following the general procedure described in Fig. S2 and characterized for their specificity towards TRPM8 channel as previously described [8,91–93] and resumed in this section as follows and supplemental material.

A reductive amination reaction as previously described [91], led to the final product 3. Intermediate 4 was synthesized starting from 5-benzyloxytryptamine which underwent to reductive amination reaction with 4-phenoxybenzaldheyde. Reaction of 3 with 2,4-dinitrosulfenyl chloride in acid media led to the final compound 5.

The synthesis of derivative 6 was performed starting from L-tryptophan methyl ester that was subjected to a double nucleophilic replacement reaction with benzyl bromide as earlier described [92].

Finally, synthesis of the final compound 9 was achieved as described earlier [93]. Intermediate 7 was obtained starting from indole-5-carboxyaldehyde which was N-methylated by reaction with methyl iodide and sodium hydride. The corresponding N-methyl derivative (7) underwent to a Mannich type reaction with formaldehyde and biphenylethyl amine, in acid media, to give intermediate 8. The final compound 9 was obtained by conducting a reductive amination of 8 using 4-fluoroaniline as a reactant.

For Patch Clamp experiments, HEK-293/TRPM8exon 1 cells were seeded 72 or 96 h before experiment using a concentration of 4 and 2.5 million cells, respectively, onto a T225 flask. Few minutes before the experiments, cells were washed twice with D-PBS without Ca^2+^/Mg^2+^ (Euroclone, Milan, Italy) and detached from the flask with trypsin−EDTA (Sigma-Aldrich, Milan, Italy; diluted 1/10). Cells were then resuspended in the suspension solution composed by 25 mL of EX-CELL ACF CHO medium (Sigma-Aldrich, Milan, Italy); 0.625 mL of HEPES (Lonza, Walkersville, USA); 0.25 mL of 100× penicillin/streptomycin (Lonza, Walkersville, USA), 0.1 mL of soybean trypsin inhibitor 10 mg/mL (Sigma-Aldrich, Milan, Italy), and located on an automated patch-clamp platform (QPatch 16X, Sophion Bioscience, Ballerup, Denmark). Menthol was used as reference agonist, and a stock solution ((1 M, 100% Dimethyl sulfoxide (DMSO)) was prepared the day of the experiment from the powder; an intermediate stock of 300 mM was prepared from the 1 M stock in 100% DMSO, and the final dilution was performed in the extracellular solution to obtain a working concentration of 300 μM (1:1000, 0.1% final DMSO concentration). Stock solutions of the testing compounds (10 mM; 100% DMSO; stored at −20 °C) were prepared the day of the experiment; an intermediate stock for each compound (300 μM) was prepared from the 10 mM stock in 100% DMSO, and the working dilutions were performed just before the experiments in the extracellular solution containing 300 μM menthol. The highest concentration tested was 300 nM, with serial dilutions (1:10) in the extracellular solution. DMSO was balanced to keep it constant throughout all the solutions in the same experiment (0.2% final DMSO concentration). Standard whole-cell voltage clamp experiments are performed at room temperature using the multihole technology. For the voltage clamp experiments on human TRPM8, data are sampled at 2 kHz. After establishment of the seal and the passage in the whole cell configuration, the cells are challenged by a voltage ramp (20 ms step at −60 mV; 100 ms ramp −60/+100 mV; 20 ms step at +100 mV; return to −60 mV) every 4 s. The potential antagonistic effect on human TRPM8 current of target compounds was evaluated after application of the agonist (menthol, 300 μM) alone and in the presence of the compound under investigation at increasing concentrations. Output: outward current evoked by the voltage ramp, measured in the step at +100 mV. The intracellular solution contained (mM) 135 CsCl, 10 BAPTA, 10 HEPES, 4 Na2ATP (pH 7.2 with CsOH). The extracellular solution contained (mM) 145 NaCl, 4 KCl, 1 MgCl_2_, 2 CaCl_2_, 10 HEPES, 10 glucose (pH 7.4 with NaOH).

For all the experiments performed in the present paper, all compounds were dissolved in DMSO at 10 mM stock concentrations and diluted in culture medium to final working concentrations as indicated. Vehicle controls contained equivalent DMSO concentrations (≤0.1%). Menthol (M2772; Sigma-Aldrich, St. Louis, MO, USA) was used as a canonical TRPM8 agonist control [8].

The lipophilic antioxidant α-Tocopherol (258024; Sigma-Aldrich), was used at 10 μM with a 18-h pre incubation. The mitochondria-targeted superoxide scavenger MitoTEMPO (NeoBiotech, Nanterre, France), was used at 25 μM with a 8-h pre-incubation. The phosphatidylinositol 3-kinase (PI3K) agonist, 740Y-P (Selleckchem, Houston, TX, USA) [94], was used at 50 μg/mL with a 2-h pre-incubation.

All compounds were freshly prepared or thawed from frozen aliquots immediately prior to each experiment.

### Cell viability and EC₅₀ determination

Cell viability was evaluated using the WST-1 colorimetric assay (#5015944001; Sigma-Aldrich). Cells were seeded in 96-well plates at a density of 5 × 10³ cells per well and treated with TRPM8 modulators for 24, 48, or 72 h. WST-1 reagent was added at a 1:10 dilution during the final 2 h of incubation. Absorbance was measured at 450 nm using a Spark plate reader (Tecan, Männedorf, Switzerland). Half-maximal effective concentrations (EC₅₀) were calculated from dose–response curves by nonlinear regression analysis (log[inhibitor] vs. normalized response) using GraphPad Prism 9 software.

### siRNA-mediated gene silencing and transfection

Small interfering RNAs (siRNAs) targeting human TRPM8 (sc-95009) and TP53 (sc-29435) were purchased from Santa Cruz Biotechnology (Dallas, TX, USA) and transfected into melanoma cells using Lipofectamine 2000 (Thermo Fisher Scientific, Invitrogen), following the manufacturer’s instructions. Cells were incubated for 48–72 h post transfection before being subjected to downstream analyzes. A non-targeting siRNA (sc-44236; Santa Cruz Biotechnology) was used as a negative control.

WM266-4 and AMM16 cells were plated at 60% confluence in 6-well plates. Once attached, cells were transfected with 2.5 µg of pEGFP-TRPM8 expression plasmid (Addgene #64879, Watertown, MA, USA) using PolyFect Transfection Reagent (Qiagen, Hilden, Germany), according to the manufacturer’s instructions. Control cells were transfected with an equal amount of empty pEGFP-C1 plasmid. Six hours after transfection, the culture medium was replaced with fresh medium. Seventy-two hours post-transfection, cells were either left unstimulated or stimulated with the indicated compounds for the specified times. Cells were then collected for Western blot analysis to assess TRPM8 expression and for propidium iodide (PI) staining to evaluate cell death.

### Live/dead assay

Cell death in melanoma cells was assessed using the Cyto3D Live–Dead Assay Kit (TheWell Bioscience, North Brunswick, NJ, USA). Dead cells were stained with propidium iodide (PI), and total cells were stained with acridine orange (AO). Imaging was performed using a DMIRB Leica microscope (Leica Microsystems, Wetzlar, Germany) equipped with either C-Plan ×10 or HCX PL Fluotar ×63 objectives. Phase-contrast and immunofluorescence images were captured with a DFC 450C camera (Leica), and merged images were generated using Leica Application Suite software. The percentage of dead cells was calculated as the ratio of red (PI-positive) to total green (AO-positive) cells, as previously described [95].

### Annexin V-FITC apoptosis assay

Melanoma cells were seeded in 24-well plates (2.5 × 10⁴ cells/well) or 96-well plates (5 × 10³ cells/well). The following day, cells were left untreated or treated with the indicated compounds at 1 µM or 10 µM for 6 h, followed by staining with Annexin V-FITC Reagent (E-CK-A111, Elabscience) according to the manufacturer’s instructions. Briefly, Annexin V Binding Buffer (10×) was diluted to 1× before use. Annexin V-FITC was diluted 1:100 in the 1× Binding Buffer and added to the cells, which were incubated at room temperature for 15–20 min in the dark.

For assays performed in 24-well plates, apoptotic cells were visualized using a Leica MICA Hub microscope in phase-contrast and fluorescence modes to detect the characteristic green membrane staining. For assays in 96-well plates, Annexin V-FITC fluorescence was quantified using a Tecan multiwell plate reader (Tecan, Männedorf, Switzerland) set at Ex/Em 490/530 nm, and values were expressed as fold change relative to basal levels.

### 3D spheroid assays

Melanoma cells (4 × 10²) were embedded in 50 µL of phenol red-free, growth factor-reduced Matrigel (10 mg/mL; BD Biosciences, Erembodegem, Belgium) mixed with 100 µL of organoid plating medium per well in a 96-well Biofloat plate (Sarstedt, Numbrecht, Germany), as previously described [89]. Spheroids were allowed to form for 3 days, after which the plating medium was replaced with a similar medium lacking Y-27632 [96]. On day 4, spheroids were either left untreated or treated for 21 additional days with the indicated drugs, with medium changes every 3 days.

For spheroids-NK cells co-coltures, NK cells were added (E:T ratio 25:1) as in [89]. Untreated or treated co-coltures were followed for 8 h.

Different fields were imaged using a Leica MICA fluorescence microscope equipped with a PL FLUOTAR 10×/0.32 objective. Organoid area was quantified using Leica Application Suite X (LAS X) software and expressed as fold change relative to basal organoid area. Cell viability within organoids was assessed using the Cyto3D Live–Dead Assay Kit (TheWell Bioscience, North Brunswick, NJ, USA) according to the manufacturer’s instructions (Di Donato et al. [89], melanoma).

### Fluorescent Ca2+ imaging

WM266-4 cells in 6 multi-wells were incubated for 60 min at 37 °C with 1 µM 4-Fluo AM (Abcam, ab241082). Cells were washed and incubated with medium as reported in ref. [8]. Cells were then left untreated or treated for 60 s with the indicated compounds. Different fields were analyzed using DMIRB Leica (Leica) microscope equipped with C-Plan × 40 or HCX PL Fluotar × 63 objectives (Leica). IF microscopy images were generated using a DFC 450C camera (Leica).

### Intracellular ROS and mitochondrial superoxide detection

Cells (5 × 10³) were plated in black 96-well plates with clear bottoms for fluorimetric assays. Intracellular ROS levels were measured using the general oxidative stress indicator 2′,7′-dichlorofluorescin diacetate (DCFDA; Fluorimetric Intracellular ROS Kit, MAK145; Sigma Aldrich). A master reaction mix (100 μL/well) was added to the cells, which were incubated for 1 h at 37 °C before treatment with compounds. ROS levels were analyzed 4 h post treatment following the manufacturer’s instructions.

Mitochondrial superoxide production was assessed using MitoSOX™ Red (Neo Biotech). Cells were either untreated or treated with selected modulators for 1.5 h, then incubated with 5 μM MitoSOX™ for 20 min at 37 °C. After incubation, cells were washed and analyzed according to the manufacturer’s protocol. In both the assays, different fields were observed and acquired using a Leica MICA fluorescence microscope equipped with a PL FLUOTAR 10×/0.32 objective. Images were acquired with Leica Application Suite X (LAS X) software.

### Mitochondrial membrane potential assessment

Mitochondrial membrane potential (Δψm) was evaluated using the JC-1 dye (#30001, Biotium, Fremont, CA, USA). Cells, plated on coverslips, were untreated or treated as reported and stained with 5 μM JC-1 for 15 min at 37 °C. Fluorescence was analyzed by a Leica MICA fluorescence microscope equipped with a PL FLUOTAR 10×/0.32 objective immunofluorescence microscopy and the Leica Application Suite X (LAS X) software.

Mitochondrial depolarization was indicated by a shift from red fluorescence (J-aggregates) to green fluorescence (monomers). Fluorescence intensities of red and green signals were quantified using NIH ImageJ software, and the ratio of red to green fluorescence was calculated for each condition to assess loss of Δψm.

### Protein extraction, co-immunoprecipitation (Co-IP) and Western blot analysis

Protein extracts were prepared using a cold lysis buffer containing 50 mM Tris-HCl (pH 7.4), 1 mM EDTA, 1% Triton X-100, 150 mM NaCl, 5 mM MgCl₂, and 1 mM EGTA, supplemented with protease and phosphatase inhibitors: 1 mM PMSF (phenylmethylsulfonyl fluoride), protease inhibitor cocktail (LAP), 1 mM sodium orthovanadate (Na₃VO₄), and 100 μg/mL aprotinin. Protein concentration was quantified using the Bio-Rad assay (Bio-Rad Laboratories, Hercules, CA, USA).

Samples (20–40 μg) were separated on 10–15% SDS-PAGE gels and transferred onto nitrocellulose membranes (Amersham Protran Premium 0.45 μm, GE Healthcare) or polyvinylidene fluoride (PVDF) membranes (Millipore). Membranes were blocked with 5% bovine serum albumine (BSA) and incubated overnight at 4 °C with primary antibodies (listed below), followed by horseradish peroxidase (HRP)-conjugated secondary antibodies. Immunoreactive proteins were detected using ECL substrate (GE Healthcare, Chicago, IL, USA).

Lysate proteins were used in co-immunoprecipitation (Co-IP) experiments or electrophoretically separated by sodium dodecyl sulfate polyacrylamide gel electrophoresis (SDS-PAGE) [90]. The following antibodies were used in WB and Co-IP analyzes:

the mouse monoclonal: anti-IκBα (L35A5) (#4814, Cell Signaling); anti-p53 (DO-1) (sc-126, Santa Cruz); anti-IKKα (3G12) (#11930, Cell Signaling); anti-α-tubulin (#E-AB-20036, Elabsciences); anti-GAPDH (#E-AB-20079, Elabsciences); the rabbit polyclonal: anti-phospho-ATM (Ser1981) (D25E5) (#13050, Cell Signaling); anti-phospho-p53 (Ser15) (#AF1043, R&D Systems); anti-phospho-AKT (Ser473) (#9271, Cell Signaling); anti-AKT (#9272, Cell Signaling); anti-phospho-GSK-3α/β (Ser21/9) (#9331, Cell Signaling); anti-caspase-3 (#9662, Cell Signaling); anti-PARP cleavage site (214/215) (#AB3565, Millipore); anti-PARP (#06-557, Upstate); anti-TRPM8 (#NBP1-97311, Novus Biologicals); anti-PMAIP1 (NOXA) (#A9801, Abclonal); the recombinant rabbit monoclonal anti-PUMA (#A3752, Abclonal); anti-ULBP1 (#A21161, Abclonal); anti-IKKβ (D30C6) (#8943, Cell Signaling); anti-phospho-IKKα/β (Ser176/180) (#2697, Cell Signaling); anti-phospho-NF-κB p65 (Ser536) (93H1) (#3033, Cell Signaling); anti-PI3K p85 (#06-195; Millipore, Burlington, Massachusetts, USA); anti-PI3K p110 δ (#AB1678; Abcam, Cambridge, UK).

Full and uncropped western blots are shown in Supplemental materials.

### Immunofluorescence (IF) and confocal microscopy

Cells were grown on glass coverslips, fixed in 4% and permeabilized with 0.2% Triton X-100 paraformaldehyde unless otherwise specified. Then, cells were blocked in 1% Fetal Bovine Serum (FBS; Gibco). To determine TRPM8 localization, AMM16 and WM266-4 cells were fixed for 10 min with 2% paraformaldehyde (w/v in PBS; Merck, Saint Louis, MO, USA) and permeabilized for 5 min with 0.1% Tween-20 (w/v in PBS; Bio-Rad, Hercules, CA, USA). Blocking was performed overnight at 4 °C in PBS containing 1% FBS (v/v). Cells were firstly incubated for 1 min with green fluorescent 3,3’-Dihexyloxacarbocyanine Iodide (DiOC6(3); 5 µg/mL) (#D273, Thermo Fisher, Waltham, MA, USA) to stain Endoplasmic Reticulum. Next, coverslips were incubated for 10 min with red fluorescent Alexa Fluor® 594 wheat germ agglutinin (WGA; 5 µg/mL) (Molecular Probes, Invitrogen Ltd, Paisley, UK) to stain the plasma membrane. Subsequently, they were incubated overnight at 4 °C with rabbit polyclonal anti-TRPM8 antibody (1:50 dilution in PBS; #NBP1-97311, Novus Biologicals). Following this, coverslips were incubated with the fluorescein-conjugated DyLight™ 405 AffiniPure anti-rabbit IgG secondary antibody (1:200 dilution in PBS containing 0.01% BSA; #111-475-045, Jackson ImmunoResearch Laboratories, West Grove, PA, USA). Specificity of the staining was confirmed by negative control slides incubated only with the secondary antibody after staining of the endoplasmic reticulum and plasma membrane.

Mitochondria and cytochrome c were stained in WM266-4 cells plated on coverslips. Cells were fixed, permeablized and blocked overnight with PBS containing 1% FBS (v/v). The following day coverslips were incubated for 30 min at 37 °C with the mitochondrial dye MitoView 633 (200 nM; #70055, Biotium, Glowing Products for Science) and overnight at 4 °C with mouse monoclonal anti-cytochrome c (CYCS) antibody (1:50 dilution in PBS; #E-AB-22110, Elabsciences, Houston, TX, USA). Secondary detection was performed using FITC-conjugated anti-mouse antibody (1:200 dilution in PBS with 0.01% BSA; #315-096-003, Jackson ImmunoResearch Laboratories). The specificity of the staining protocol was verified by omitting the primary antibody and incubating the coverslips only with the secondary antibody, following mitochondrial staining with MitoView 633.

For γH2AX and p53 staining, cells were fixed, permeabilized, and blocked with 1% FBS for 1 h, and then incubated overnight at 4 °C with primary antibodies (rabbit anti-γH2AX, 1:50; mouse anti-p53, 1:50, respectively). After washing, goat anti-rabbit Texas red-conjugated antibody (Jackson ImmunoResearch; 1:250) and goat anti-mouse Texas red-conjugated antibody (Jackson ImmunoResearch; 1:250) were used, respectively. Nuclei were counterstained with DAPI.

In all staining procedures, coverslips were mounted in Mowiol (Sigma-Aldrich). Confocal images were acquired using the Leica MICA Microsystems (Wetzlar, Germany) confocal microhub station, equipped with an HC PL APO CS2 63×/1.20 NA water immersion objective (WD 220 µm). Image acquisition and analysis were performed using the Leica Application Suite X (LAS X) software.

### NK cell-mediated cytotoxicity assay

Human NK cells were obtained from Voden Medical Instruments (Meda, Italy). They were co-cultured with melanoma cells pre-treated with TRPM8 modulators for 21 days, at effector-to-target (E:T) ratios of 25:1 and 50:1, for 8 h. Where indicated, blocking antibodies against NKG2D (sc-53501) or ULBP1 (#A21161; Abclonal) were added 60 min prior to co-culture. Cytotoxicity was assessed as previously described [89].

### Colony formation assay

Cells were seeded in 6-well plates (500 cells/well) in RPMI-1640 medium supplemented with 0.5% FBS, 100 μ/mL penicillin, and 2 mM glutamine. Cells were treated with TRPM8 modulators at 1 or 10 μM for 21 days. At the end of the treatment, colonies were fixed with 4% paraformaldehyde, stained with 0.5% crystal violet, and manually counted. Images were acquired using a flatbed scanner.

### Data mining

TRPM8 gene expression analysis by RNA-Seq was conducted using data from 461 melanoma samples and 558 normal skin tissues. The dataset was obtained from The Genomic Data Commons–The Cancer Genome Atlas (GDC–TCGA) and accessed via the Gene Expression Profiling Interactive Analysis, version 2 (GEPIA2; http://gepia2.cancer-pku.cn/#index). Normal tissue data were derived from the combined TCGA and GTEx (Genotype-Tissue Expression) reference dataset, as implemented in GEPIA2. Differential expression analysis was performed using the built-in module, applying a |log₂ fold change| ≥ 1 and a *q*-value < 0.01 as statistical thresholds.

To investigate TRPM8 expression within the immune and stromal compartments of the melanoma tumor microenvironment, we analyzed publicly available single-cell RNA sequencing (scRNA-seq) data from the Tumor Immune Single-cell Hub (TISCH; http://tisch.comp-genomics.org/). Specifically, we used the “Melanoma [97]” dataset (GEO accession number: GSE115978), which includes thousands of cells isolated from metastatic melanoma samples. This dataset provides detailed annotations of malignant, immune, and stromal cell populations. For our analysis, only non-malignant cell types were retained, including T cells, B cells, NK cells, macrophages, cancer-associated fibroblasts, and endothelial cells. Raw gene expression count matrices were pre-processed and normalized using the standardized Seurat v3-based pipeline provided by TISCH.

Dimensionality reduction was performed using t-distributed stochastic neighbour embedding (t-SNE) to explore cellular heterogeneity and TRPM8 expression patterns. TRPM8 expression was visualized using log₂-transformed TPM values (log₂(TPM + 1)). Two parallel t-SNE plots were generated: 1. a clustering map to identify distinct immune and stromal cell populations; 2. an expression map displaying TRPM8 intensity using a continuous color gradient.

### Statistical analysis

All experiments were performed at least in triplicate unless otherwise specified. Data are presented as mean ± standard deviation (SD). Statistical analyzes were conducted using one-way or two-way Analysis of variance (ANOVA) followed by appropriate post hoc tests (Bonferroni), or unpaired two-tailed Student’s *t*-tests, as appropriate. Analyzes were performed using GraphPad Prism version 9 (GraphPad Software, San Diego, CA, USA). *P*-values < 0.05 were considered statistically significant.

## Supplementary information

Supplemental Material

Full and uncropped Western Blots

## Data Availability

All data generated and analyzed in the present study are included in this article and supplemental materials. Supplemental figures and full and uncropped western blots are shown in Supplemental materials.
