# Supplementary material for: Rewiring melanoma cell fate: TRPM8 modulators trigger apoptosis and boost NK cell cytotoxicity
Source: Cell Death Dis. 2026 Feb 14;17(1):223. doi: 10.1038/s41419-026-08469-8 (PMC12921236; doi:10.1038/s41419-026-08469-8)
Supplement: Supplementary file 1 — Supplemental Material [file 41419_2026_8469_MOESM1_ESM.docx]

##

**Supplemental Material**

**Rewiring Melanoma Cell Fate: TRPM8 Modulators Trigger Apoptosis and Boost NK Cell Cytotoxicity**

**
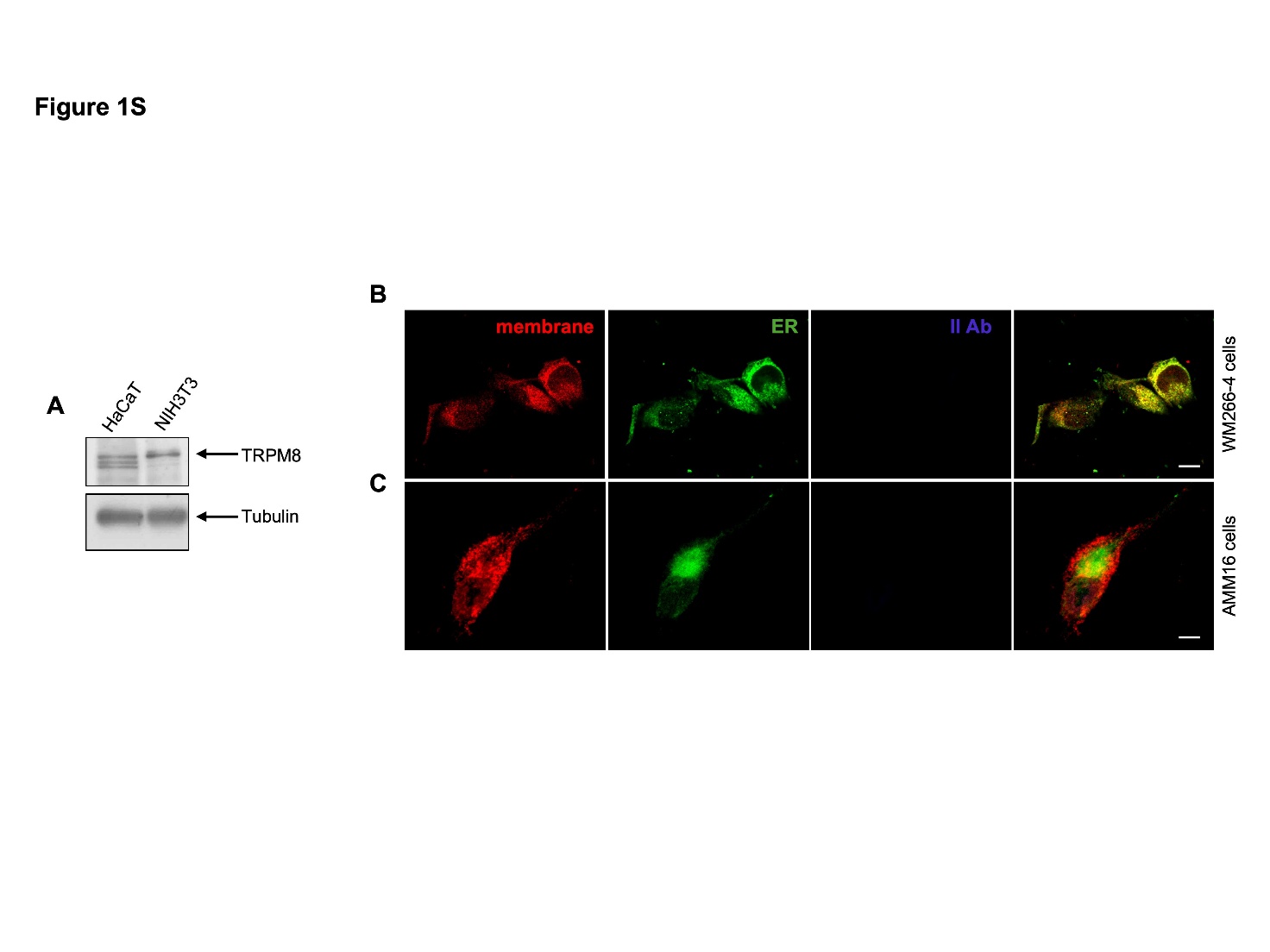
Figure 1S:**

1. Representative western blot showing TRPM8 protein expression in the indicated cells. α-Tubulin was used as a loading control. **(B, C)** WM266-4 and AMM16 cells were firstly stained for endoplasmic reticulum (ER) and plasma membrane, then incubated with the fluorescent secondary antibody in the absence of primary antibody to assess non-specific binding (II Ab). No fluorescence signal was detected in either AMM16 or WM266-4 cells. Scale bar: 10 μm.


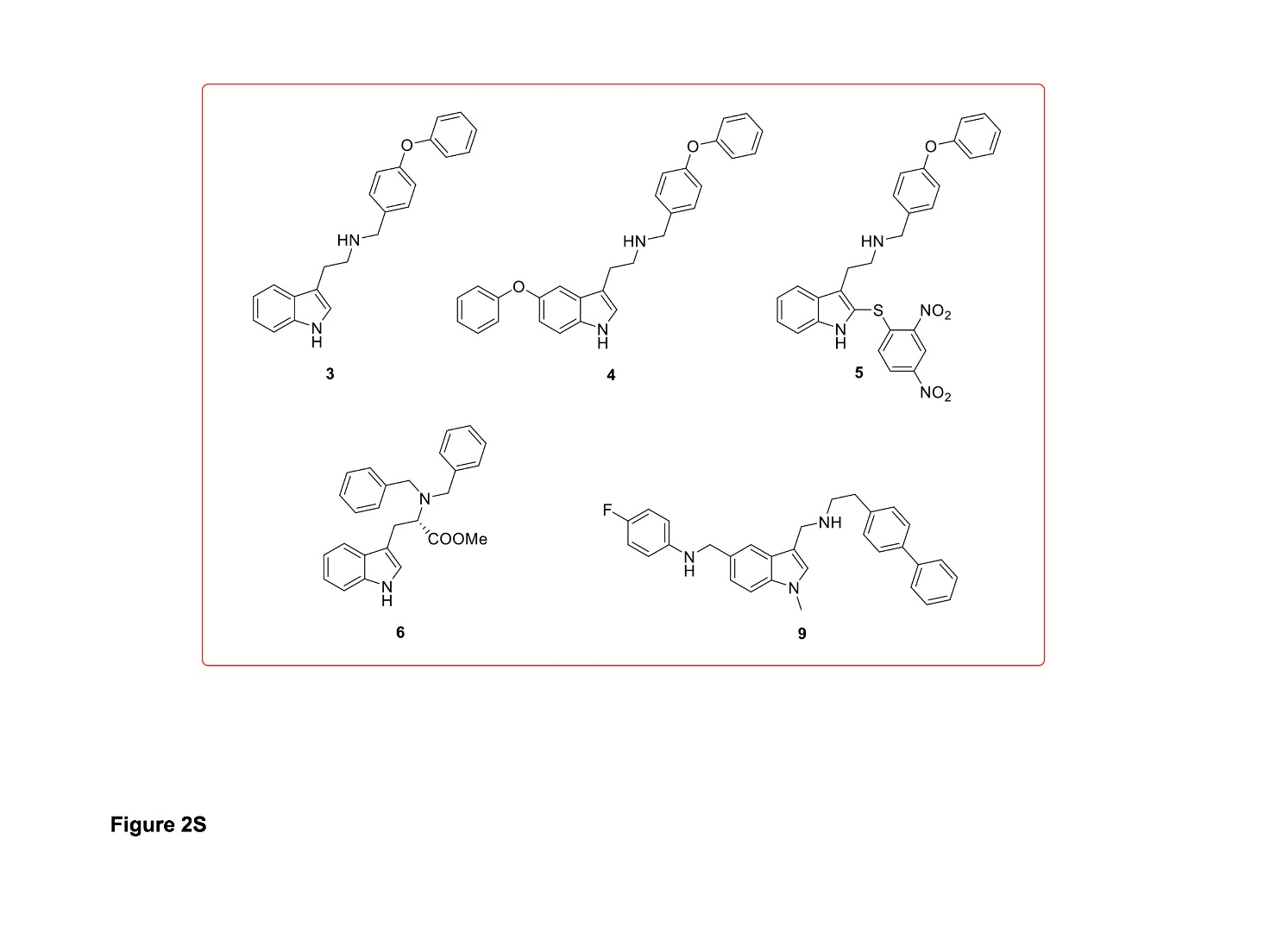

**Figure 2S:** Molecular structures of the tested compounds (upper panel). Synthesis of compounds (Lower panel).

***2-(1H-indol-3-yl)-N-(4-phenoxybenzyl)ethanamine*** (**3**)

Compound **3** was synthesized starting from tryptamine and 4-phenoxybenzaldehyde in 85% yield as previously described^90^. 1H and 13C NMR spectra were in accordance with literature.

***2-(5-(benzyloxy)-1H-indol-3-yl)-N-(4-phenoxybenzyl)ethanamine (4)***

Intermediate 5-benzyloxytryptamine was reacted with 4-phenoxybenzaldehyde using the same conditions previously described^90^. After flash-chromatographic purification compound **4** was isolated in 89% yield. 1H NMR (400 MHz, CD3OD) δ 3.15 (t, 2H, C*H*2, *J* = 8.0

Hz ); 3.31 (t, 2H, C*H*2, *J* = 8.0 Hz); 4.18 (s, 2H, C*H*2); 5.11 (s, 2H, C*H*2); 6.92 (d, 1H, aryl, *J* = 8.0 Hz); 7.03 (t, 3H, aryl, *J* = 8.0 Hz); 7.12-7.20 (m, 3H, aryl); 7.31 (d, 2H, aryl, *J* = 8.0 Hz); 7.33 (s, 1H, aryl); 7.35 (t, 2H, aryl, *J* = 8.0 Hz); 7.38-7.49 (m, 7H, aryl); 13C NMR (100 MHz, CD3OD) δ 22.0; 48.2; 50.3; 70.8; 101.6; 111.9; 112.3; 118.3; 119.1; 123.6; 123.7; 123.8; 127.0; 127.3; 127.4; 128.0; 129.7; 131.3; 131.4; 132.4; 137.9; 152.9. ESI-MS m/z calcd for C30H28N2O2, 448.56; found 448.60.

***2-(2-((2,4-dinitrophenyl)thio)-1H-indol-3-yl)-N-(4-phenoxybenzyl)ethanamine (5)***

146 mg of compound **3** (0.41 mmol) were dissolved in 10 mL of CH3COOH/DMF (8:2 v:v) and maintained under magnetic stirring. To this solution 192 mg (0.82 mmol) of 2,4- dinitrophenylsulphenyl chloride were added. The mixture was allowed to react at room temperature under nitrogen stream for 2 hours. Then, a saturated solution of NaHCO3 was added and the pH adjusted to 8. The resulting aqueous phase was extracted two times with dichloromethane. Organic phases were collected, dried over Na2SO4, filtered and evaporated in vacuo. Crude product was purified by flash chromatography giving **5** as a yellowish solid in 76% yield.

1H NMR (400 MHz, CDCl3) δ 2.89 (t, 2H, C*H*2, *J* = 8.0 Hz ); 3.03 (t, 2H, C*H*2, *J* = 8.0 Hz); 3.71 (s, 2H, C*H*2); 4.62 (s, 1H, NH); 6.76 (t, 3H, aryl, *J* = 8.0 Hz); 6.87 (d, 2H, aryl, *J* = 8.0 Hz); 7.01 (t, 1H, aryl, *J* = 8.0 Hz); 7.08-7.18 (m, 3H, aryl); 7.23 (t, 3H, aryl, *J* = 8.0 Hz); 7.29 (d, 1H, aryl, *J* = 8.0 Hz); 7.59 (d, 1H, aryl, *J* = 8.0 Hz); 7.91 (d, 1H, aryl, *J* = 8.0 Hz ); 8.69 (s, 1H, NH); 8.93 (s, 1H, aryl); 13C NMR (100 MHz, CDCl3) δ 24.5; 48.3; 52.0; 111.7; 118.4; 118.8; 118.9; 119.9; 120.7; 121.4; 123.4; 123.7; 125.0; 127.2; 127.5; 128.8; 129.7; 129.9; 132.1; 137.9; 143.9; 144.8; 146.5; 156.7; 157.0; 176.4. ESI-MS m/z calcd for C29H24N4O5S, 540.15; found 540.18.

***(S)-methyl 2-(dibenzylamino)-3-(1H-indol-3-yl)propanoate (6)***

Compound **6** was obtained by nucleophilic substitution of *L*-tryptophan methyl ester using an excess of (bromomethyl)benzene in the same conditions described previously^91^. 1H and 13C NMR spectra were in accordance with literature.

***1-methyl-1H-indole-5-carbaldehyde*** *(****7****)*

Intermediate **7** was synthesized in 85% of yield starting from indole-5-carboxyaldehyde and methyl

iodide, using NaH as base as previously described (reference 34 of the reference list). 1H and 13C NMR spectra were in accordance with literature.

***3-(((2-([1,1'-biphenyl]-4-yl)ethyl)amino)methyl)-1-methyl-1H-indole-5-carbaldehyde (8)***

The reaction of **7** with formaldehyde and 4-biphenylethyl amine using trifluoroacetic acid as catalyst as described before (reference 34 of the reference list) gave compound **8**. It was isolated in 74% yield after flash chromatography. 1H and 13C NMR spectra were in accordance with literature.

***N-((3-(((2-([1,1'-biphenyl]-4-yl)ethyl)amino)methyl)-1-methyl-1H-indol-5-yl)methyl)-4-***

***fluoroaniline (9)***

Compound **9** was synthesized by reaction of **8** with 4-fluoroaniline under the conditions described elsewhere^92^. The crude product was purified by column chromatography using mixtures of DCM/MeOH as eluent leading to the corresponding 3,5- bisaminomethyl indole derivative **4** in 61% of yield. 1H and 13C NMR spectra were in accordance with literature.

**
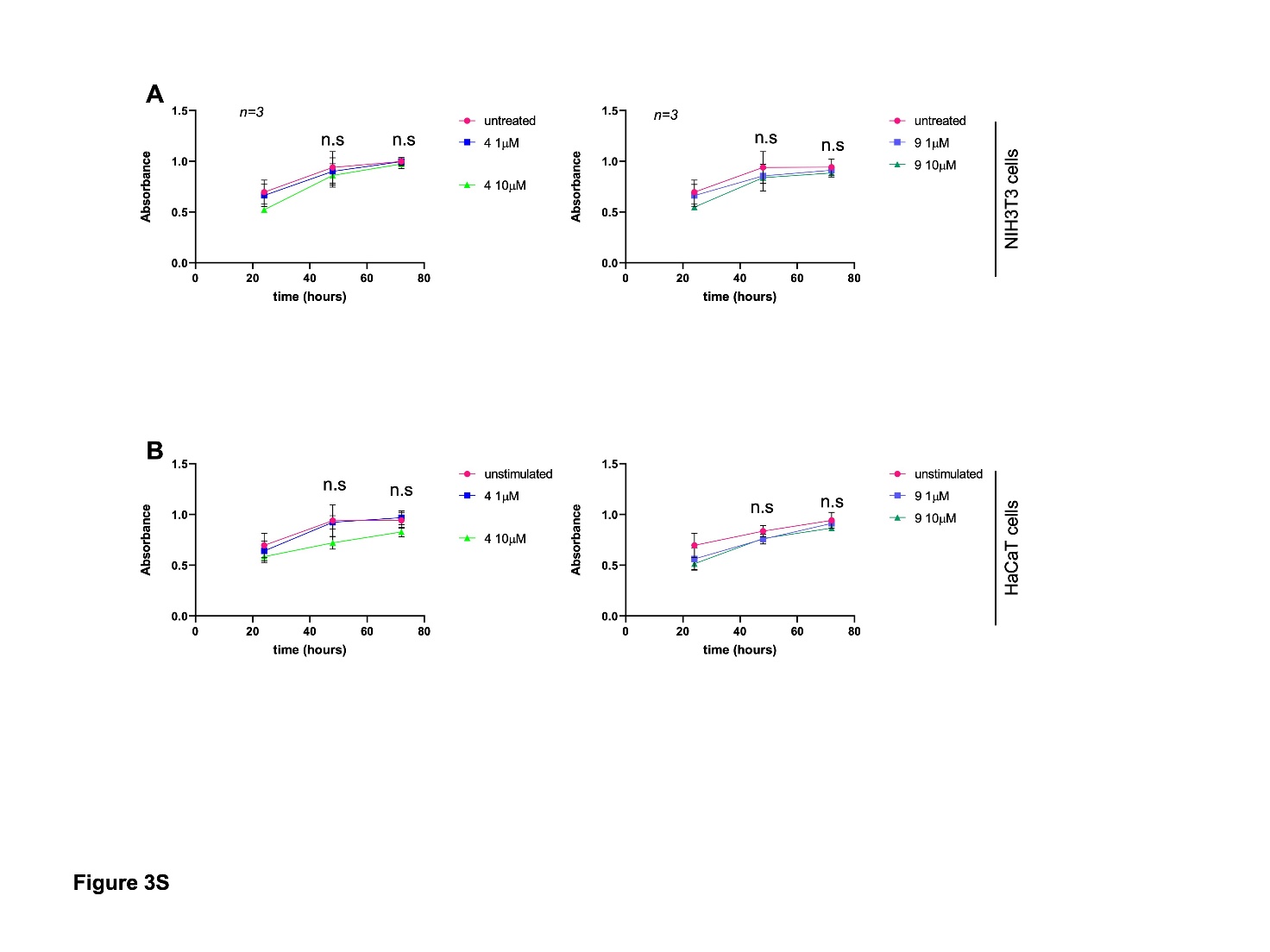
**

**Figure 3S:** Viability of NIH3T3 fibroblasts **(A)** and HaCaT keratinocytes **(B)** untreated or treated with compounds 4 and 9 at the concentrations indicated in the legends on the right. Absorbance values from WST-1 assays after 24, 48 and 72 hours are reported. Data are expressed as mean ± standard deviations (SDs) from 3 independent experiments. n.s stands for not significant

**
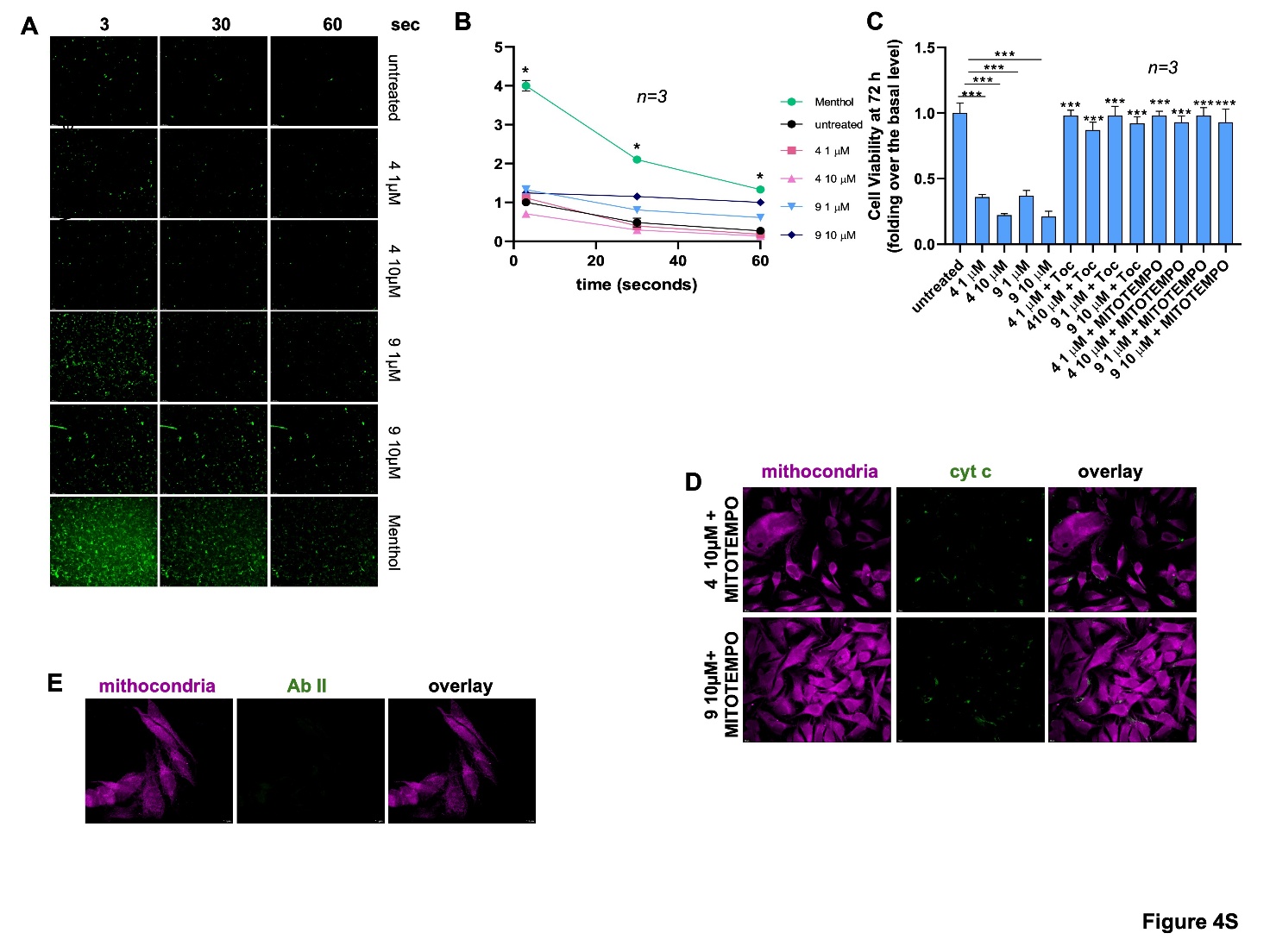
Figure 4S. (A)** TRPM8 modulators do not alter intracellular Ca²⁺ levels in WM266-4 cells. Cells were loaded with 1 μM Fluo-4 AM, as described in Methods, and either left untreated or stimulated for 60 seconds with the indicated TRPM8 modulators (at 1 or 10 μM) or menthol (10 μM, positive control). Representative fluorescence images were acquired using a Leica DFC450C camera. Scale bar: 10 μm.

**(B)** Quantification of Fluo-4 AM fluorescence is shown as fold increase over basal levels after 60 seconds of treatment. Data represent the mean ± SEM of *n = 3* independent experiments.

**(C)** WM266-4 cells were seeded in 96-well plates and left untreated or treated for 72 hours with compounds 4 and 9 (1 μM and 10 μM), in the presence or absence of α-tocopherol or MitoTEMPO used as described in Methods. Cell viability was measured using the WST-1 assay. Data are expressed as fold change over the basal point. *p < 0.05, ** p< 0.01; *** p< 0.001 one-way ANOVA with post hoc test.

**(D)** WM266-4 cells were pre-treated MitoTEMPO**,** and then treated with the indicated compounds. Mitochondria (violet) and cytochrome c (green) were stained and analyzed by confocal microscopy. In panel **(E),** cythocrome c specificity of the staining was confirmed by using MitoTracker and a secondary antibody-only control (II Ab).

**
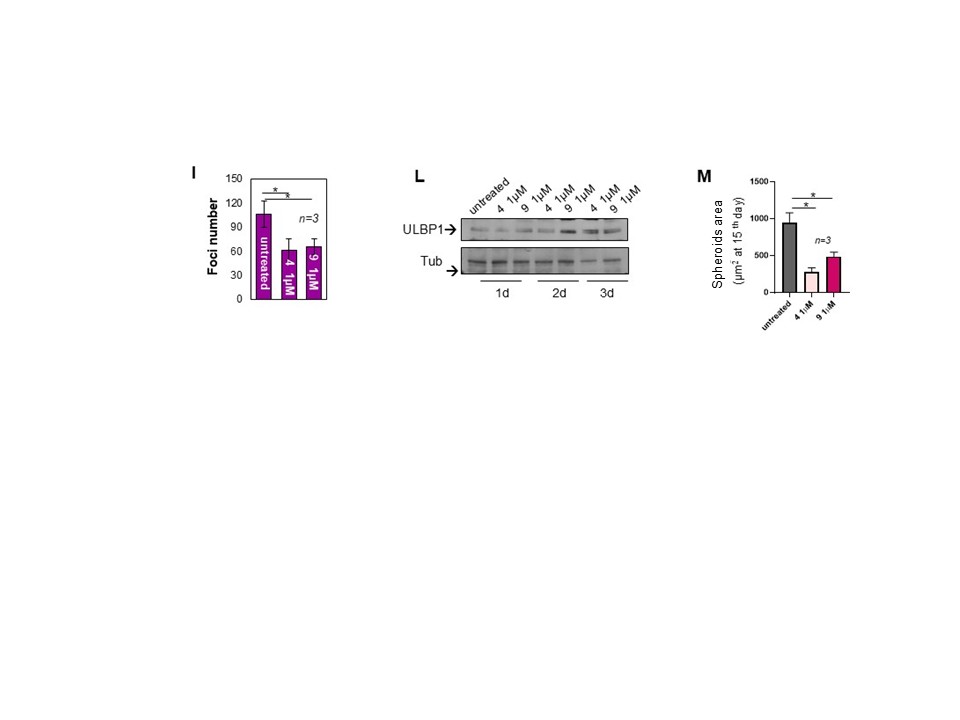

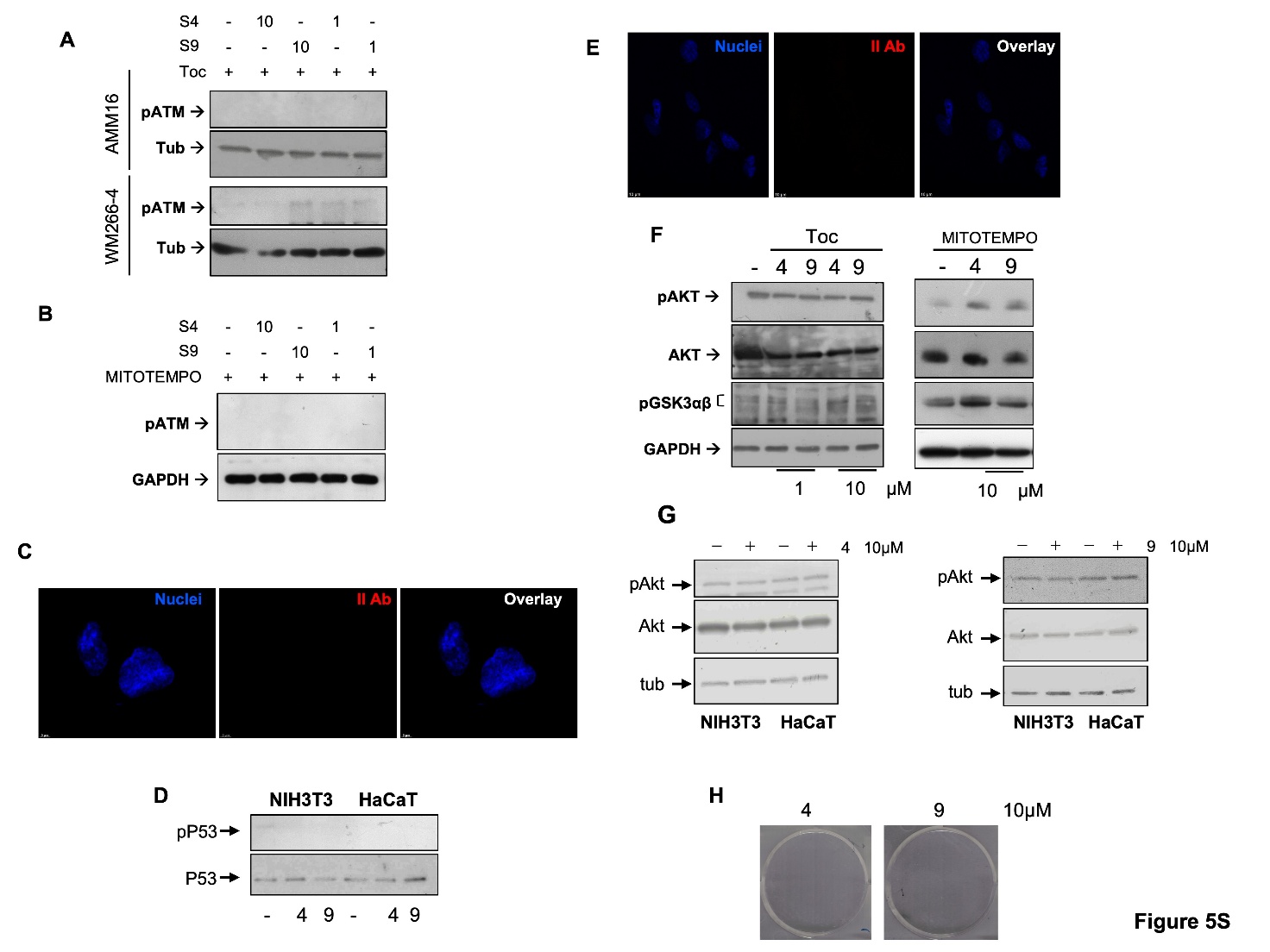
**

**Figure 5S:** AMM16 **(A)** or WM266-4 **(A, B)** melanoma cells were pre-treated with α-tocopherol (**A**) and MitoTEMPO (**B**) and then challenged with compounds 4 and 9. **(A, B)** Western blot analysis of cell lysates collected after 7 hours of treatment, using the indicated antibodies. **(C)** Representative immunofluorescence images of WM266-4 cells left treated with TRPM8 modulators (10 μM, 7 h). Staining specificity was confirmed using the Texas Red-conjugated secondary antibody alone as a control. Nuclei are stained in blue. Merged images are shown. Scale bar: 2.5 μm. **(D)** NIH3T3 and HaCaT cells were untreated or treated as indicated for 7 hours. Western blot analysis of cell lysates using the indicated antibodies is shown. **(E)** Representative immunofluorescence images of WM266-4 cells treated with TRPM8 modulators (10 μM, 7 h). Staining specificity was confirmed using Texas Red-conjugated secondary antibody alone as a control. Nuclei are counterstained in blue. Merged images are shown. Scale bar: 10 μm.
**(F)** WM266-4 cells were pre-treated with α-tocopherol (left panel) or MitoTEMPO (right panel) and then challenged with compounds 4 and 9 at the indicated concentrations. Western blot analysis of cell lysates collected after 2 or 4 hours of treatment, using the indicated antibodies is shown. **(G)** NIH3T3 and HaCaT cells were untreated or treated as indicated for 4 hours. Western blot analysis of cell lysates using the indicated antibodies is shown. **(H)** Representative images of crystal violet-stained colonies after 21-day treatment with TRPM8 modulators used at 10μM. **(I)** Quantification of colony number (foci number derived from WM266-4 cells untreated or treated with TRPM8 modulators at 1μM for 21 days) from three independent experiments (mean ± SD). **(L)** WM2664 cells were unstimulated or stimulated as indicated in the figure for different days and then collected and lysed. Western blot analysis was performed using the indicated antibodies. α-Tubulin was used as a loading control**. (M)** WM266-4-derived spheroids were left untreated or treated with compounds 4 or 9 (1 μM) and stained for Live/Dead analysis as indicated in figure 9. Quantification of spheroid area was done as reported in Methods. Data in (**I** and **M**) are presented as mean ± SD from *n = 3* independent experiments. Statistical significance was determined. *p < 0.05, **p < 0.01.


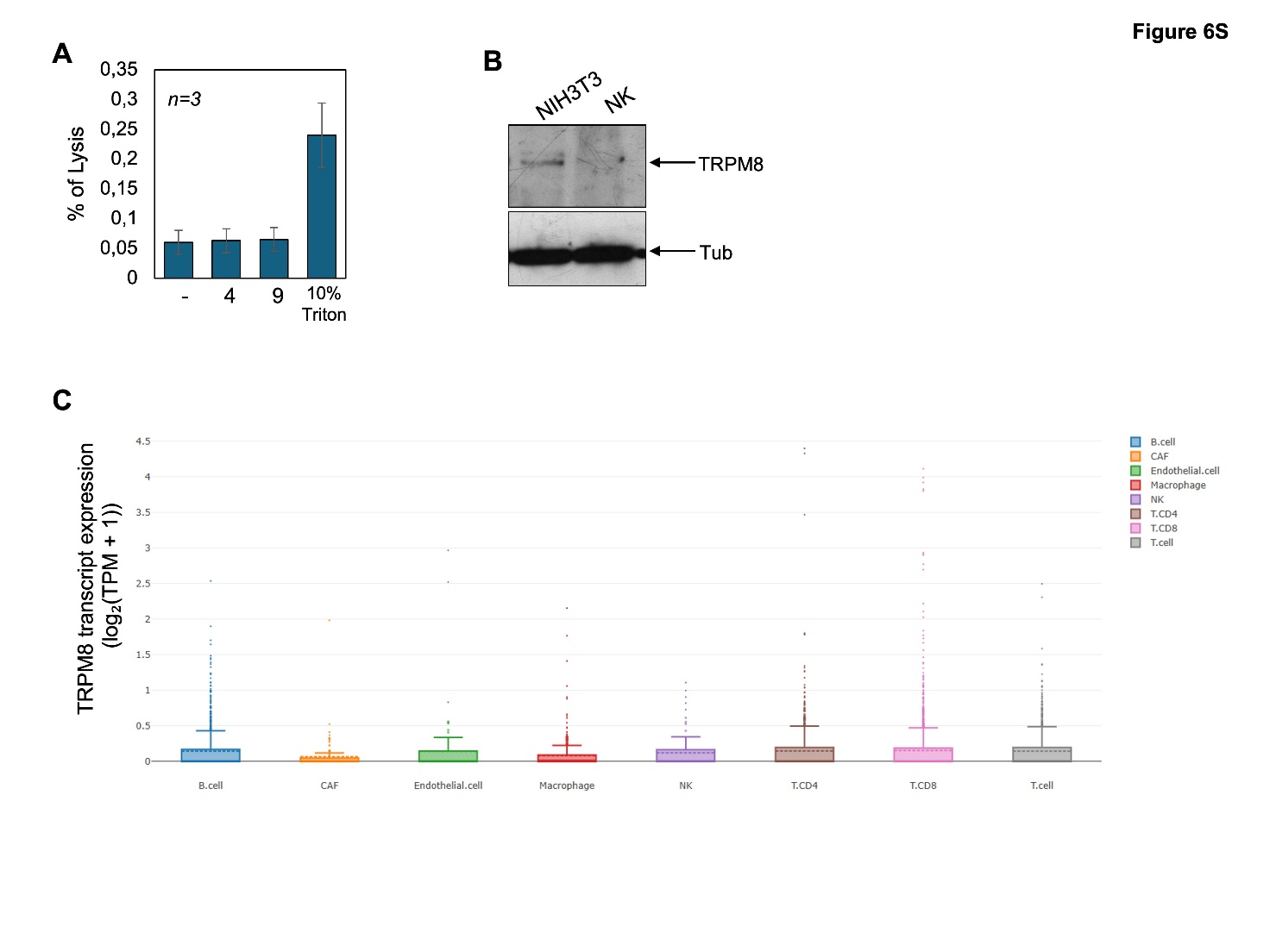


**Figure 6S. TRPM8 modulators do not induce cytotoxicity in NK cells, which lack significant detection of TRPM8 protein expression**

**(A)** NK cells were treated for 9 hours with TRPM8 compounds 4 and 9 (used at 1 μM). No significant variation in the percentage of lysis was observed between untreated and treated NK cells. 10% Triton X-100 was used as a positive control for lysis and showed a significant increase in cytotoxicity compared to untreated cells (p < 0.01). Data represent mean ± SD from three independent experiments (*n = 3*).

**(B)**Representative WB showing TRPM8 protein expression in the indicated cells. α-Tubulin was used as a loading control. TRPM8 protein was almost undetectable in NK cells.

**(C)**Boxplot of TRPM8 transcript expression (log₂(TPM + 1)) across annotated non-malignant cell types derived from the Melanoma ^73^ dataset (GEO accession: GSE115978), accessed via the Tumor Immune Single-cell Hub (TISCH; <http://tisch.comp-genomics.org/>). Expression levels were calculated from normalized single-cell RNA-seq data. Notably, NK cells exhibit low detectable TRPM8 transcript levels.

**
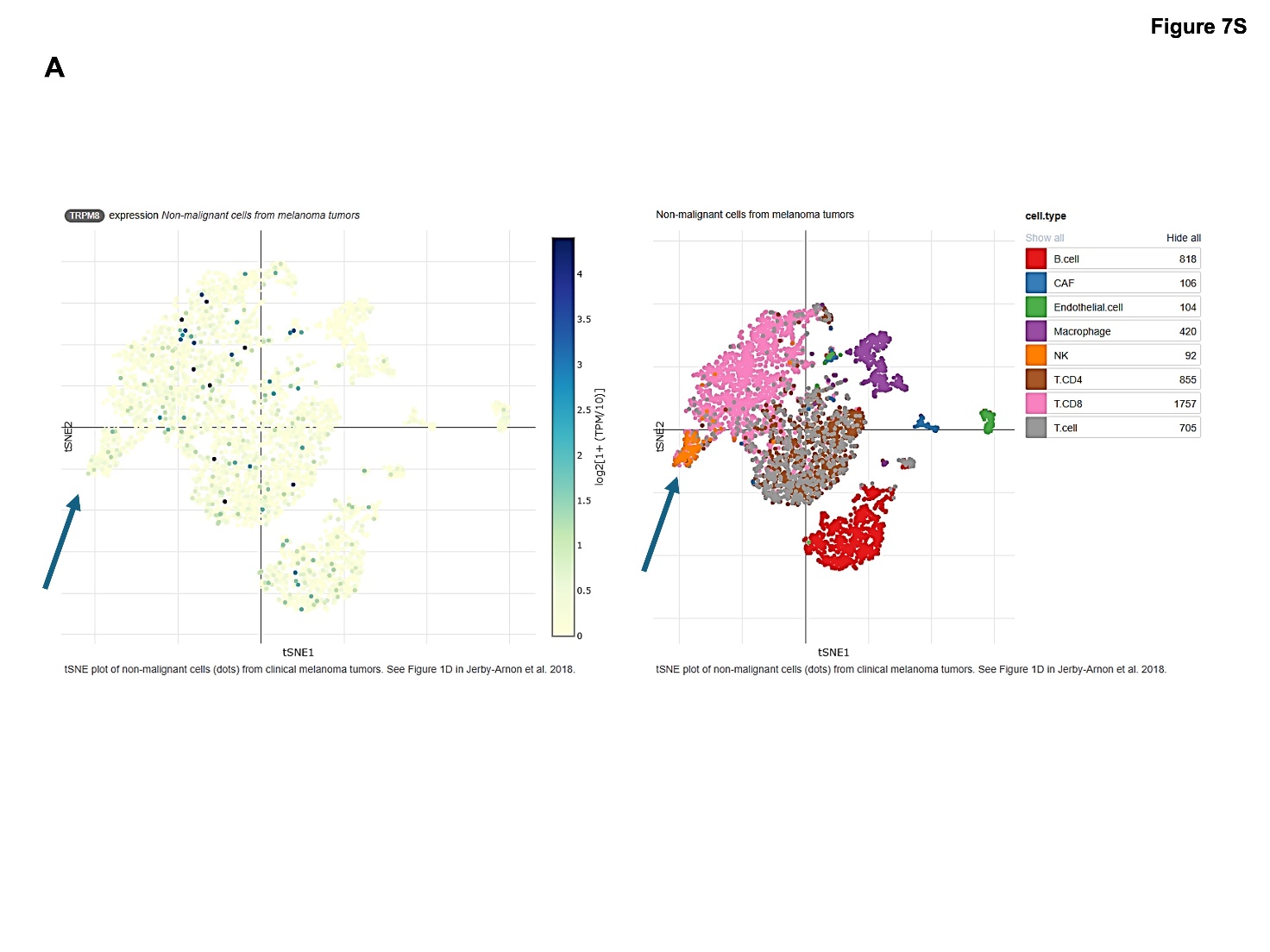
**

**Figure 7S. Expression of TRPM8 across non-malignant cell types in melanoma tumors.**

1. Left: t-SNE plot showing the expression of TRPM8 (log2 TPM+1) in non-malignant cells isolated from metastatic melanoma tumors (^73^ GSE115978). Each dot represents a single cell; color intensity reflects TRPM8 expression levels.
   Right: t-SNE plot of the same cells colored by annotated cell type, including NK cells, T cells (CD4⁺, CD8⁺), B cells, macrophages, CAFs, and endothelial cells.

**
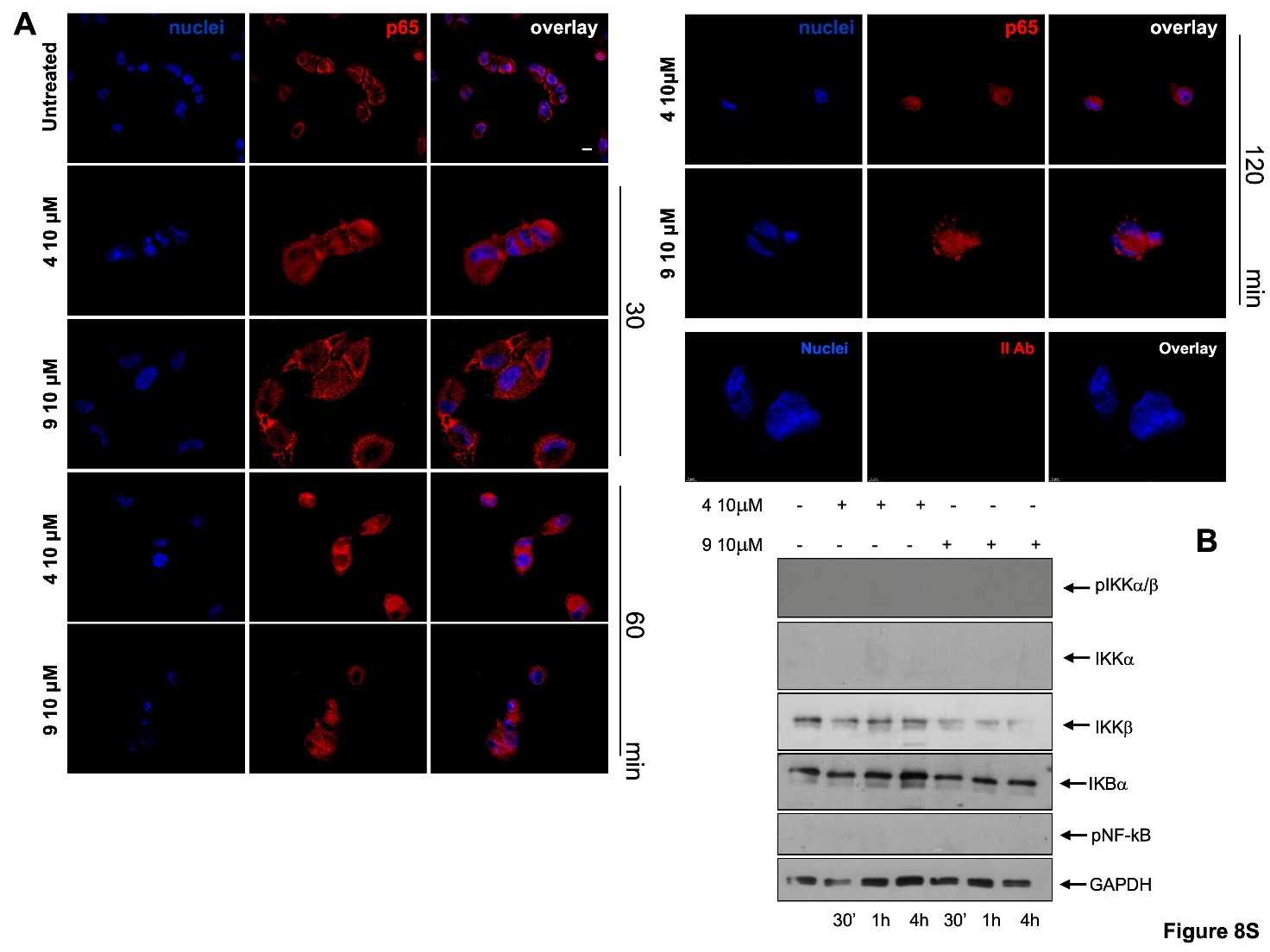
**

**Figure 8S. TRPM8 modulators do not activate canonical or non-canonical NF-κB signaling pathways in melanoma cells**

1. Subcellular localization of NF-κB p65 was analyzed by immunofluorescence in untreated and treated melanoma cells. In untreated cells, p65 (red signal) is predominantly cytoplasmic, with nuclear localization observed in only ~2–3% of cells, consistent with basal NF-κB activity. Upon 30-minute treatment with 4 and 9 TRPM8 modulators, nuclei exhibit an increase in size, and the p65 signal becomes more diffusely distributed in the cytoplasm. The cytoplasmic pattern includes branched structures and heterogeneous staining intensities, yet without detectable nuclear translocation. At later time points (60 to 120 min, up to 5 h; not shown), p65 remains excluded from the nucleus. Negative controls are shown in the right panels and include the staining of nuclei and the use of secondary antibody (Texas Red) alone.
2. Western blot analysis of canonical and non-canonical NF-κB pathway components in untreated and treated cells following 30 min, 1 h, and 4 h exposure to TRPM8 modulators (compounds 4 and 9). No phosphorylation of IKKα (non-canonical) was observed at any time point; total IKKα protein was undetectable. IKKβ (canonical) was expressed but not phosphorylated. Similarly, no phosphorylation of NF-κB p65 was detected, and IκBα levels remained unchanged over time. GAPDH was used as a loading control.
