# Supplementary material for: Rewiring melanoma cell fate: TRPM8 modulators trigger apoptosis and boost NK cell cytotoxicity
Source: Cell Death Dis. 2026 Feb 14;17(1):223. doi: 10.1038/s41419-026-08469-8 (PMC12921236; doi:10.1038/s41419-026-08469-8)

full and uncropped
original
Western blots

Figure 1B

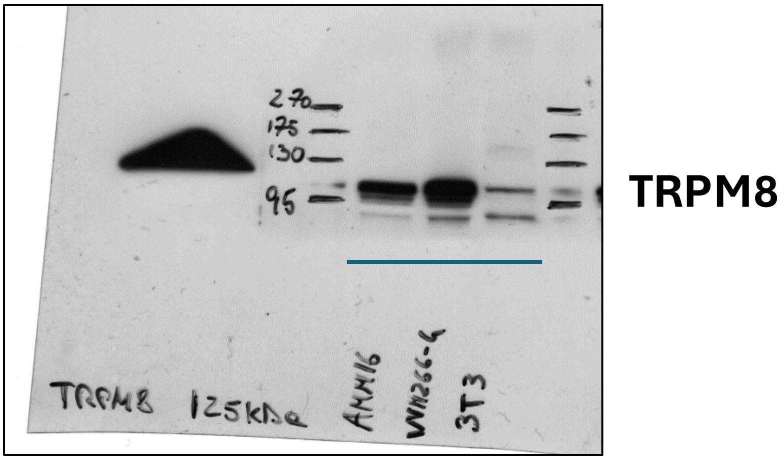
270
175
130
95
TRPM8 125kDa
AMM16
WM266-4
3T3
TRPM8

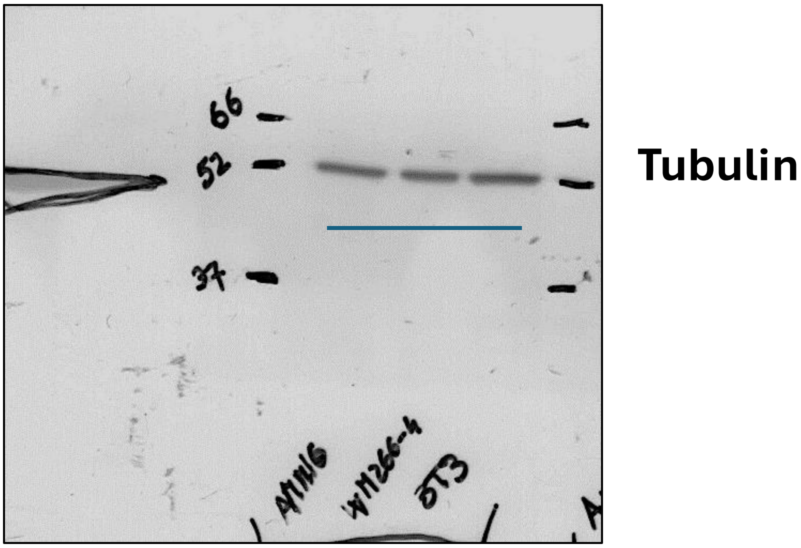
66
52
37
AMM16
WM266-4
3T3
Tubulin

Figure 2A

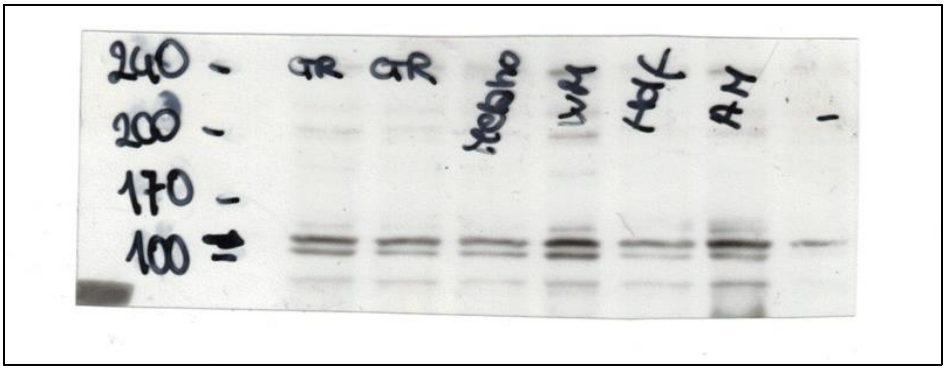

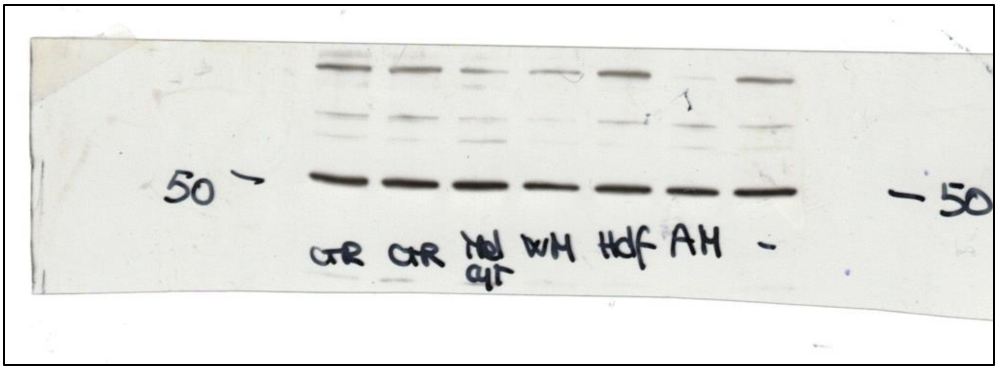

Figure 4 A

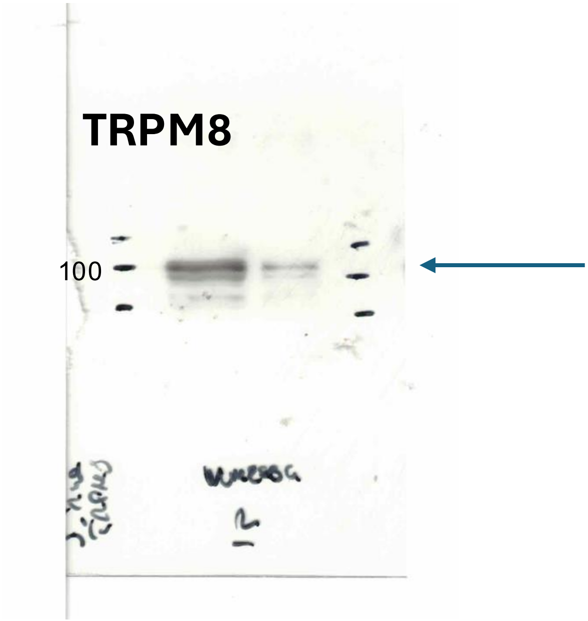

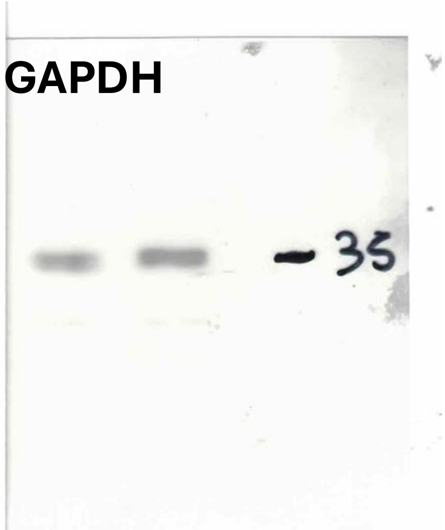

Figure 4D

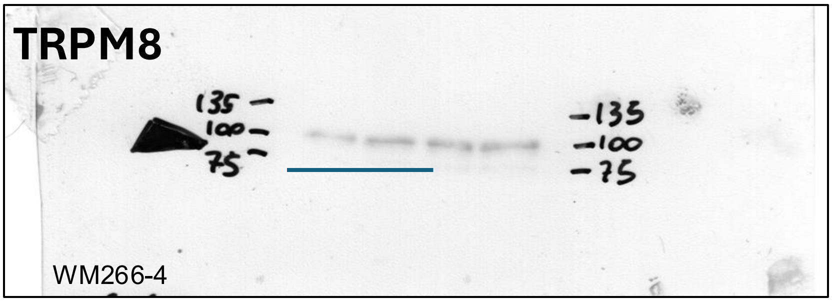

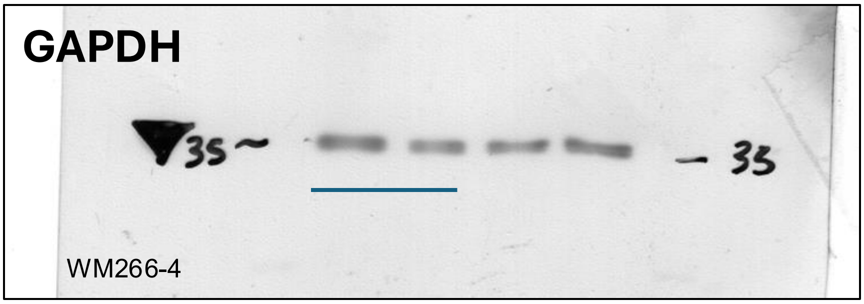

Figure 4F

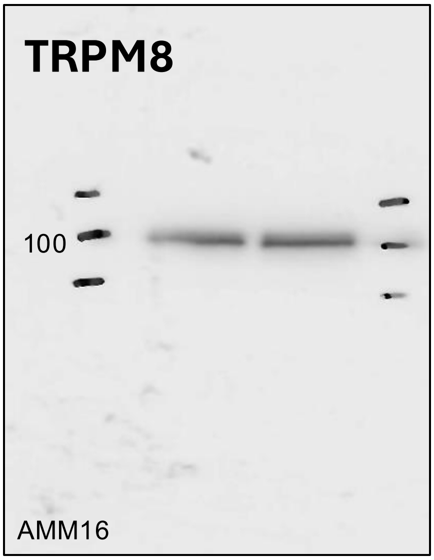

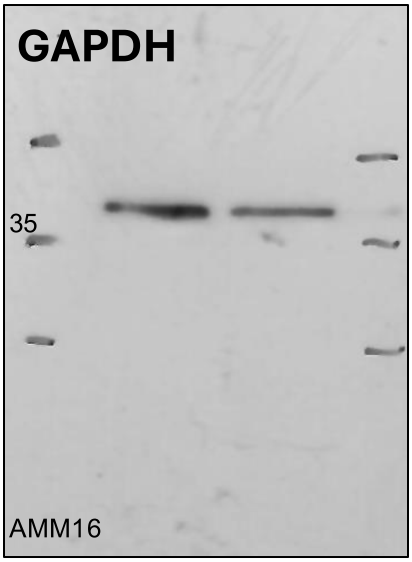

Figure 6C

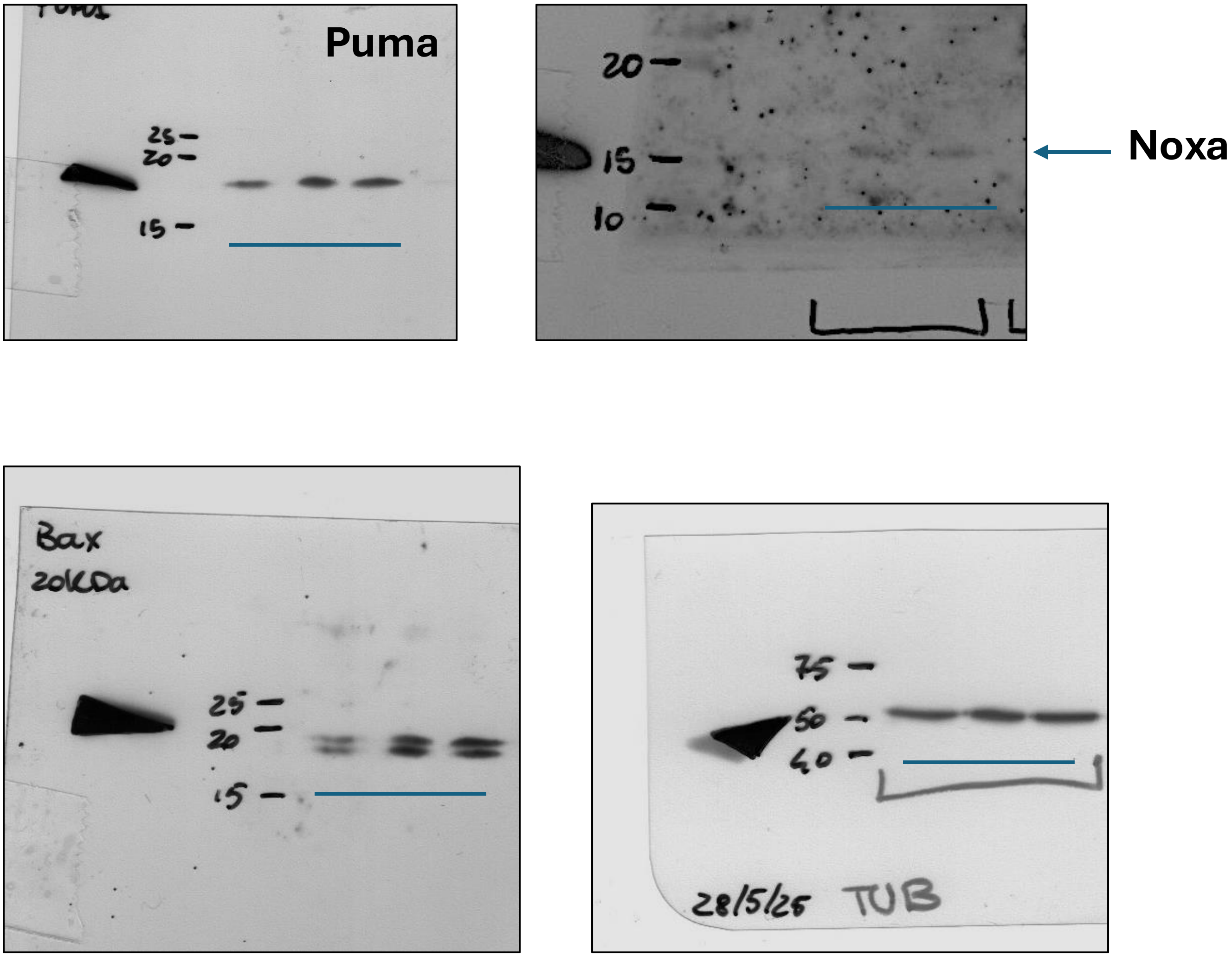

Figure 7 A

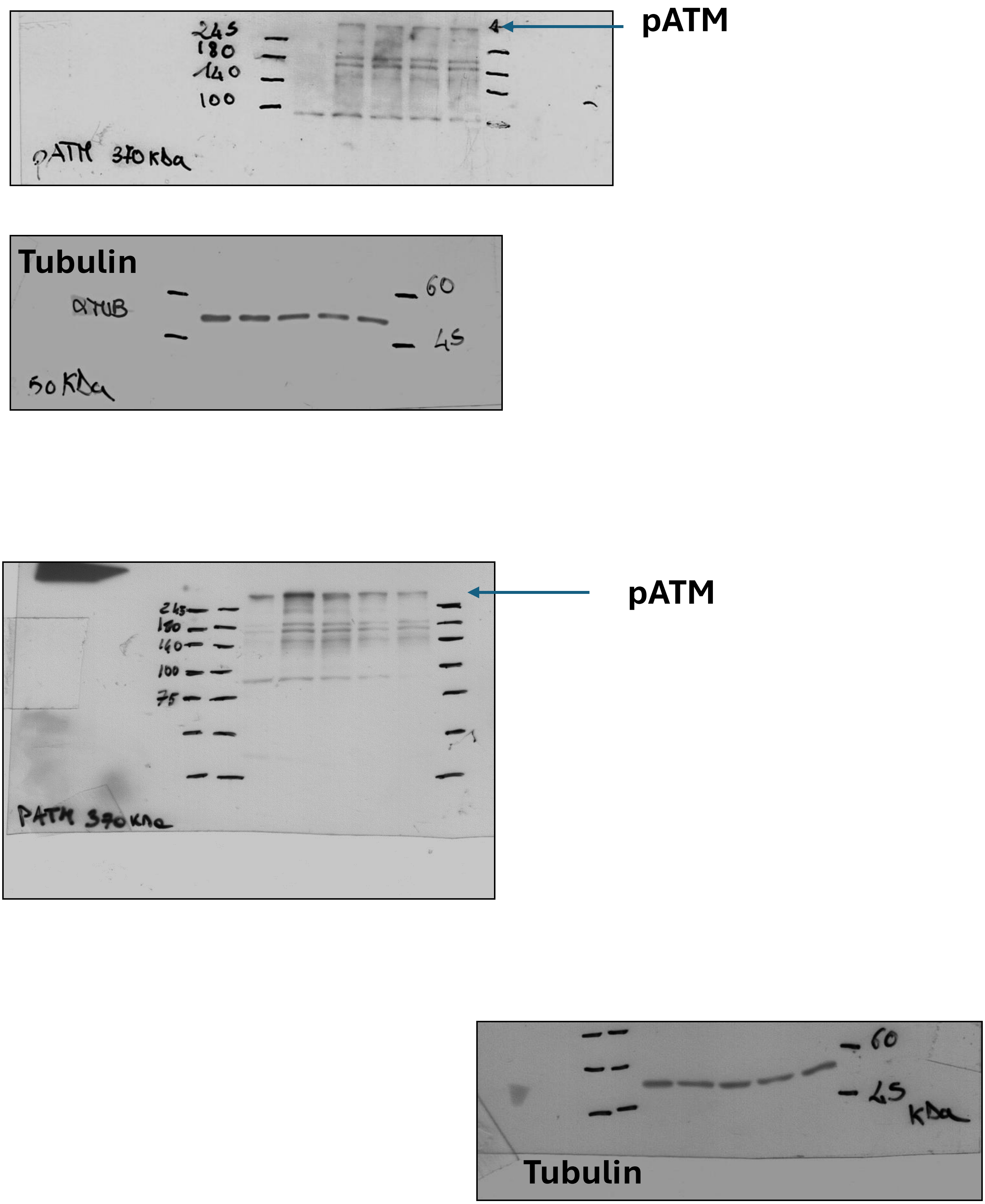

Figure 7C

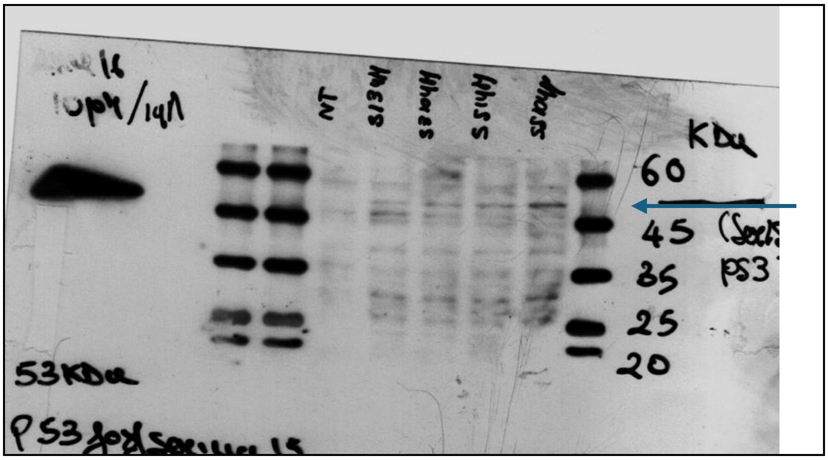

pP53

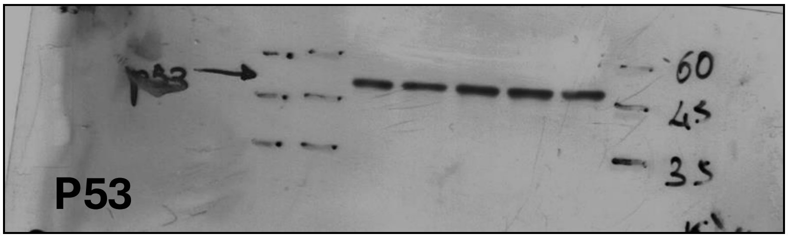

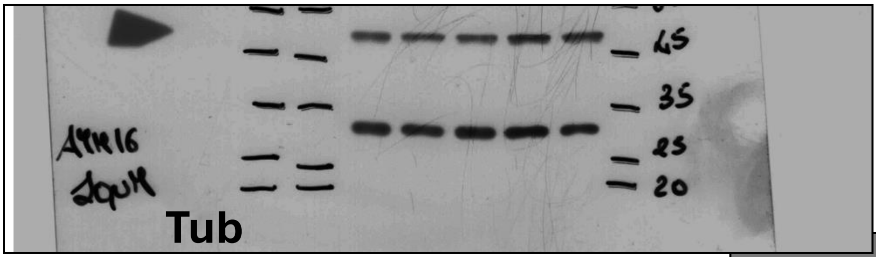

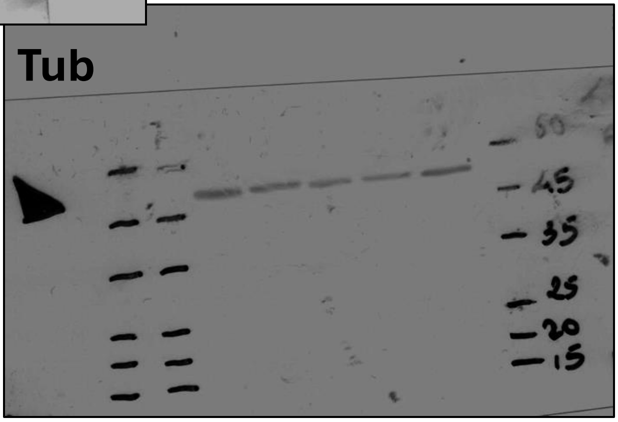

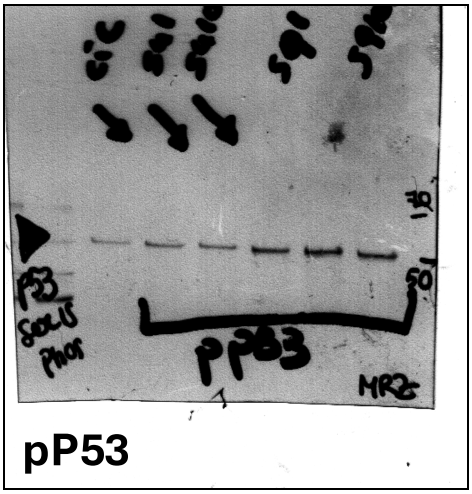

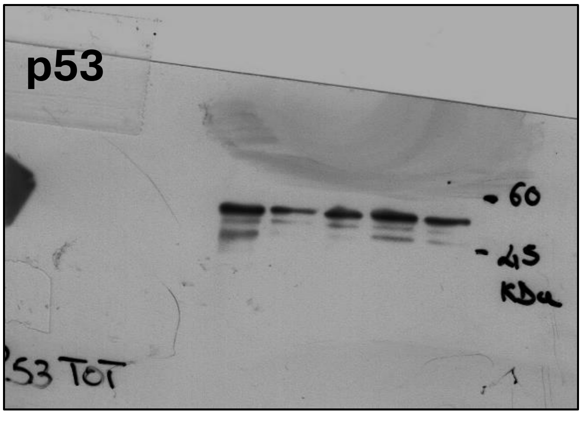

Figure 8 A

Left panel

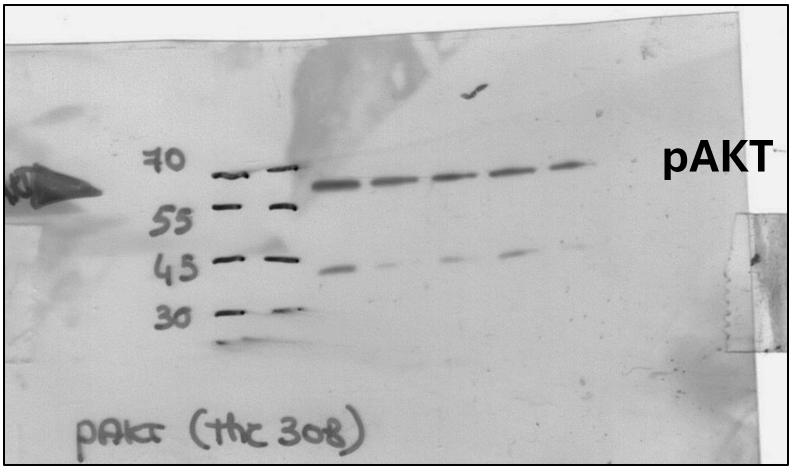

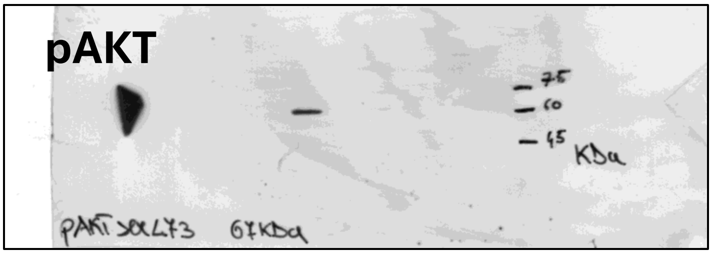

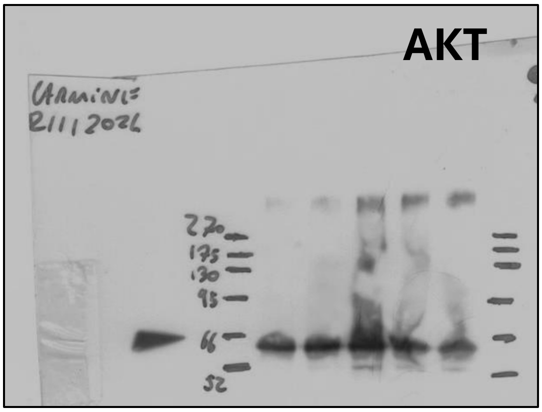

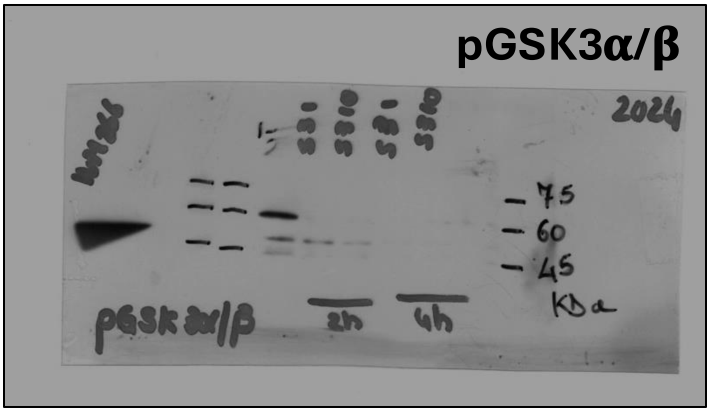

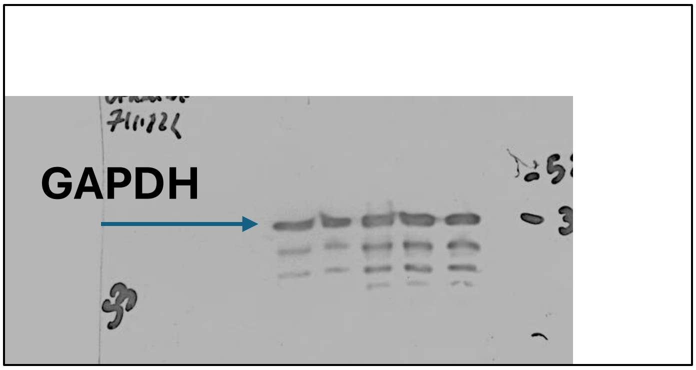

Figure 8 A

Right panel

pAKT

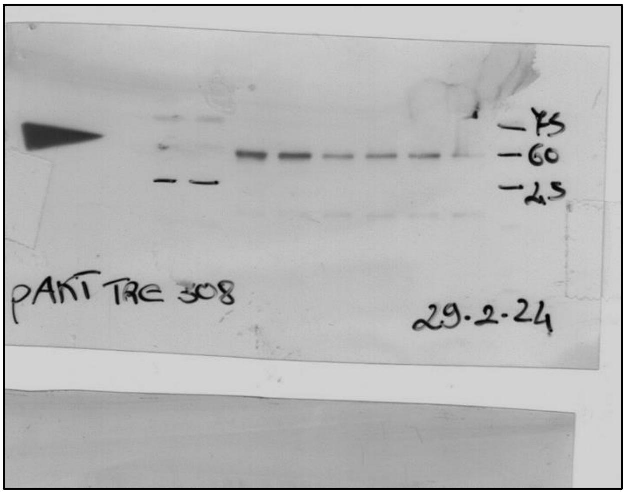

AKT

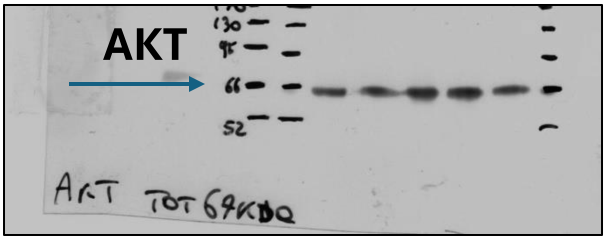

pAKT

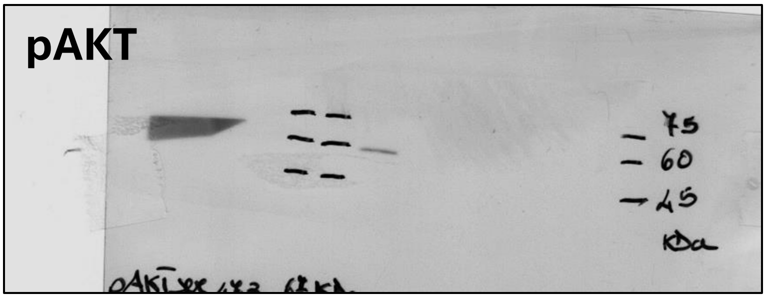

pGSK3α/β

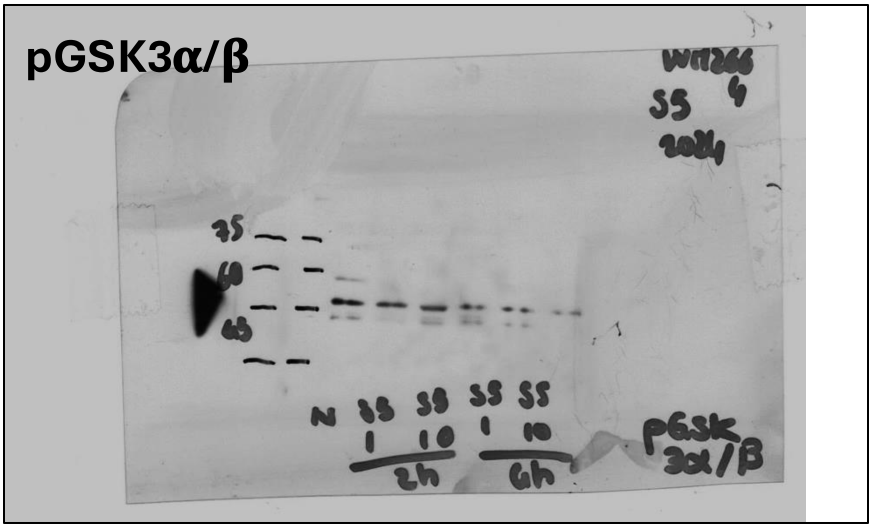

GAPDH

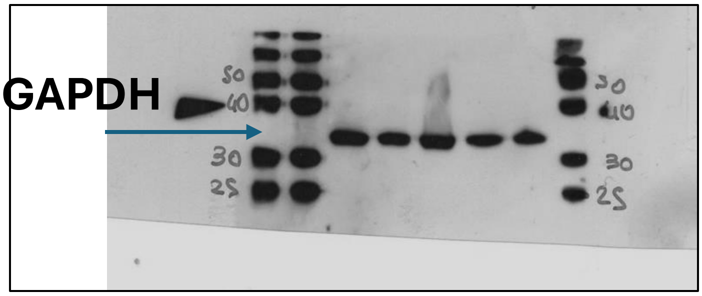

Figure 8B

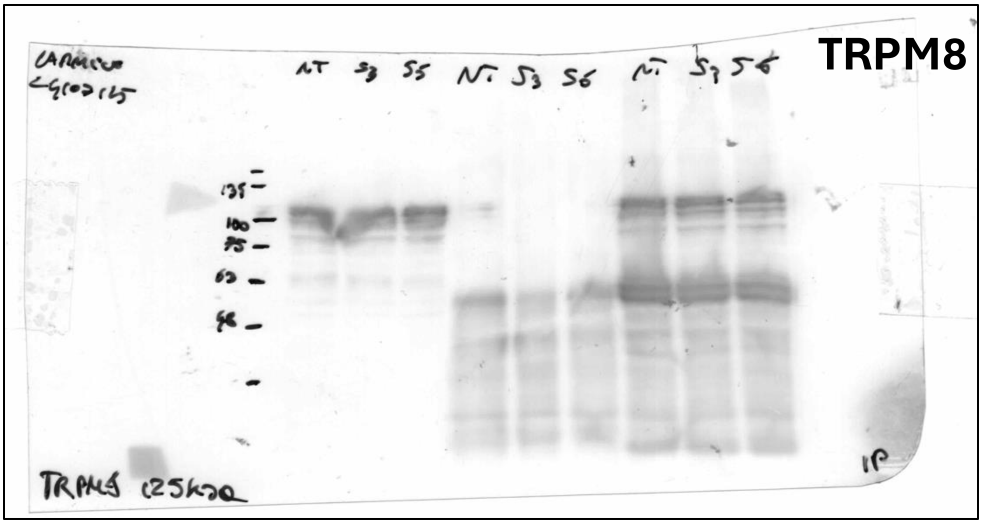

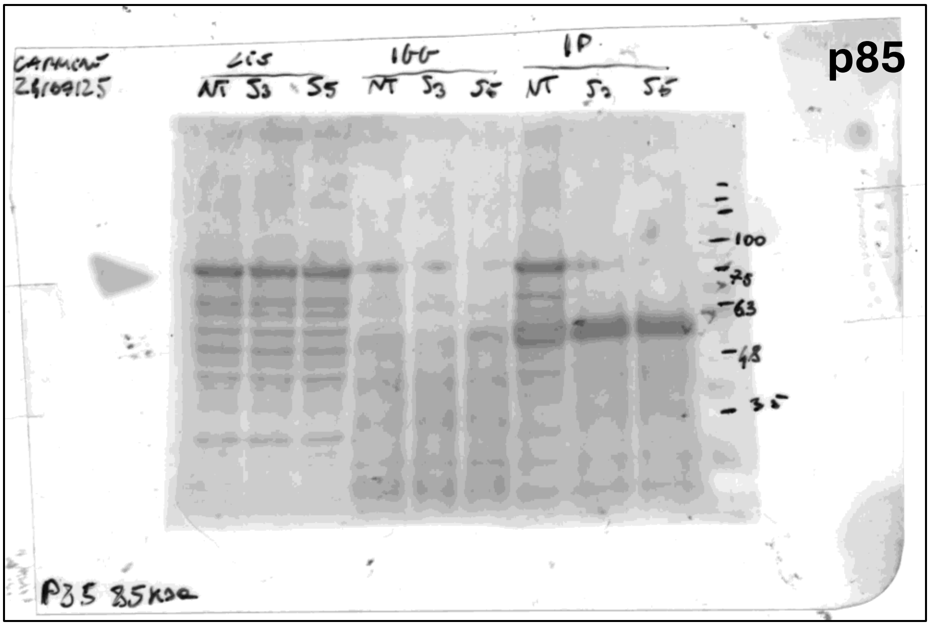

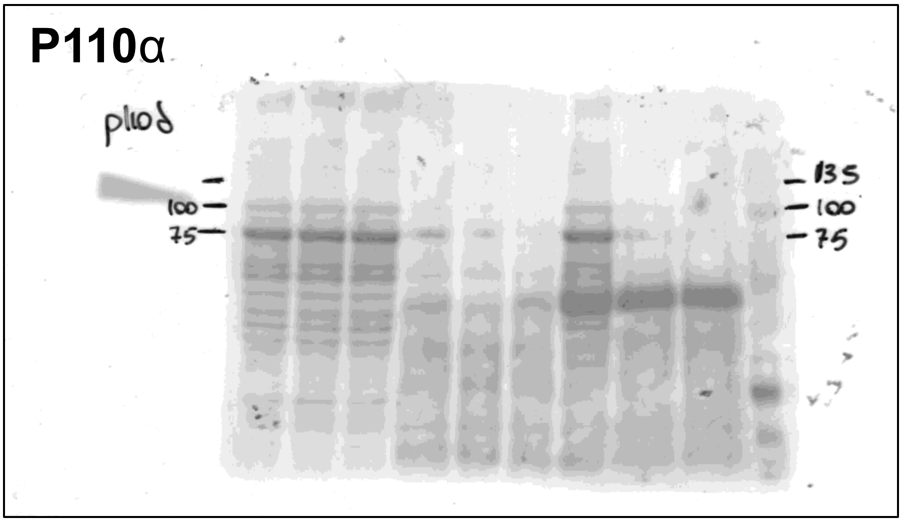

Figure 8C

Left panel

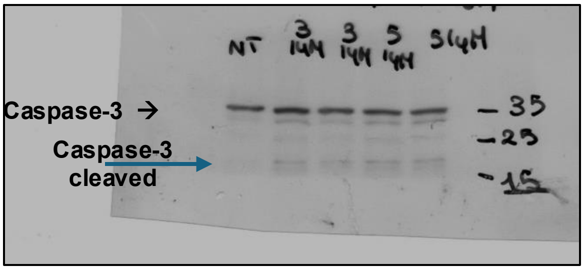

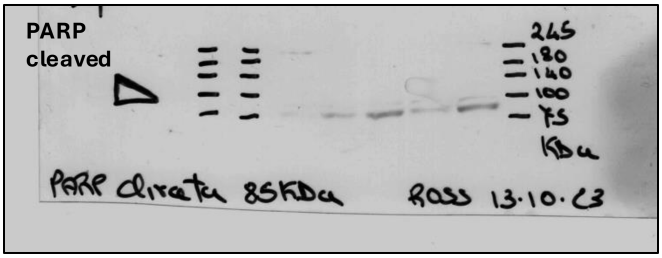

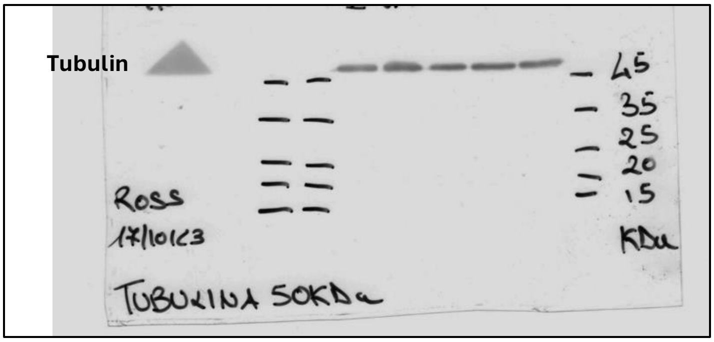

Figure 8C

Right panel

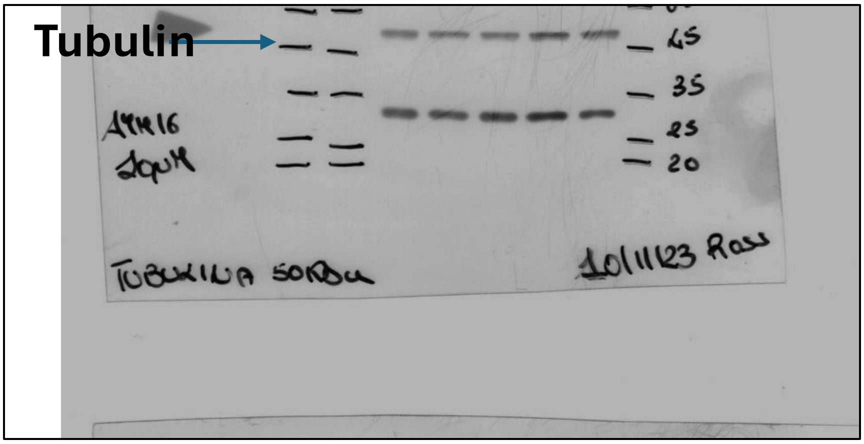

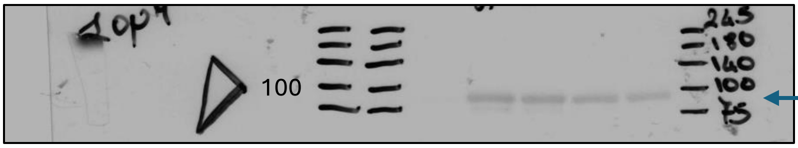

PARP cleaved

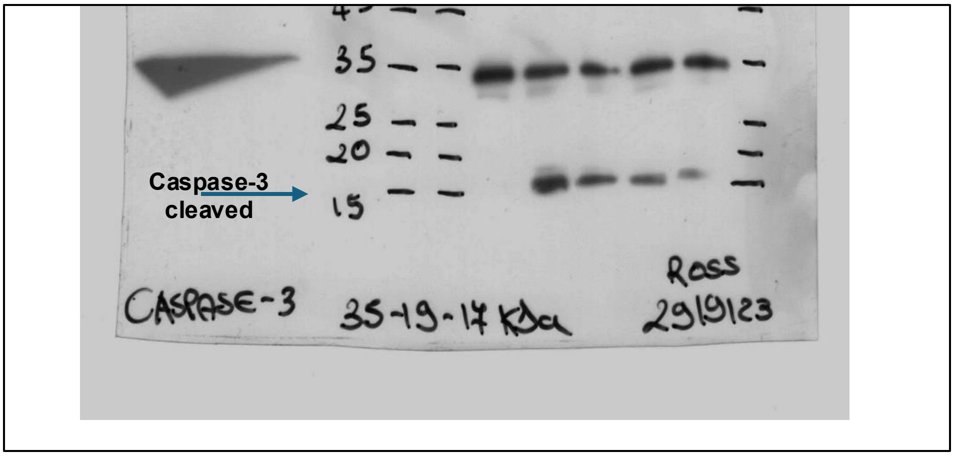

Figure 8D

Right panel

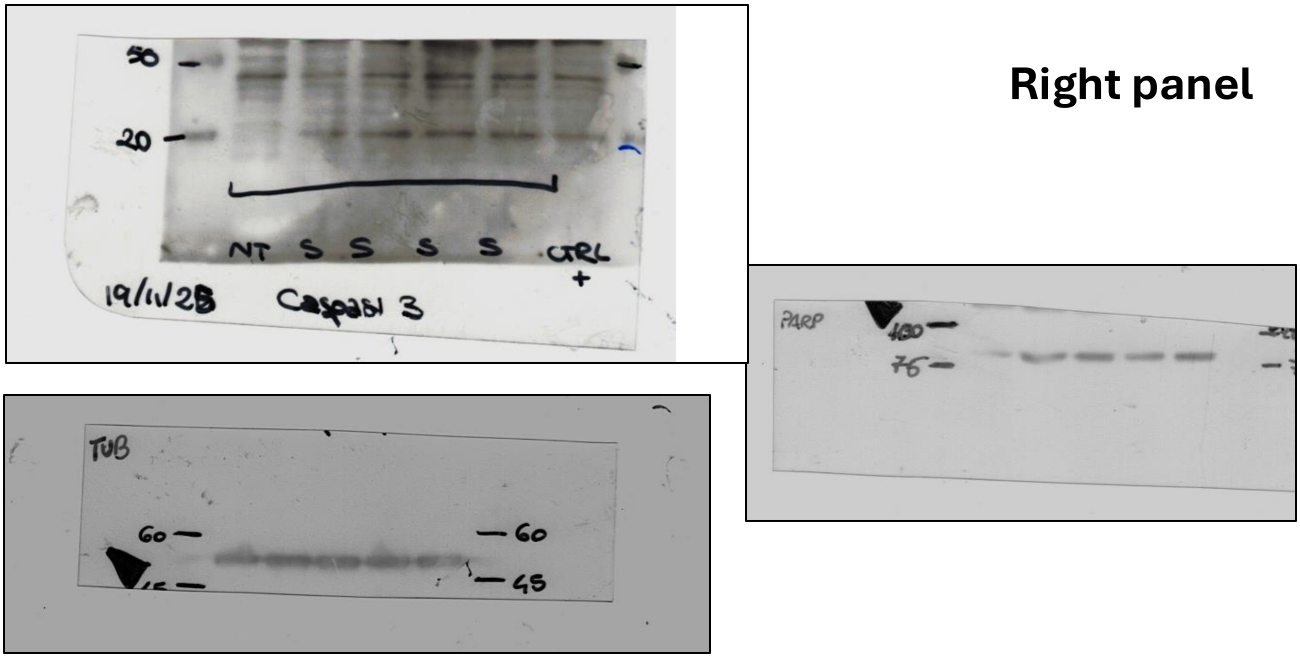

Figure 8D

Left panel

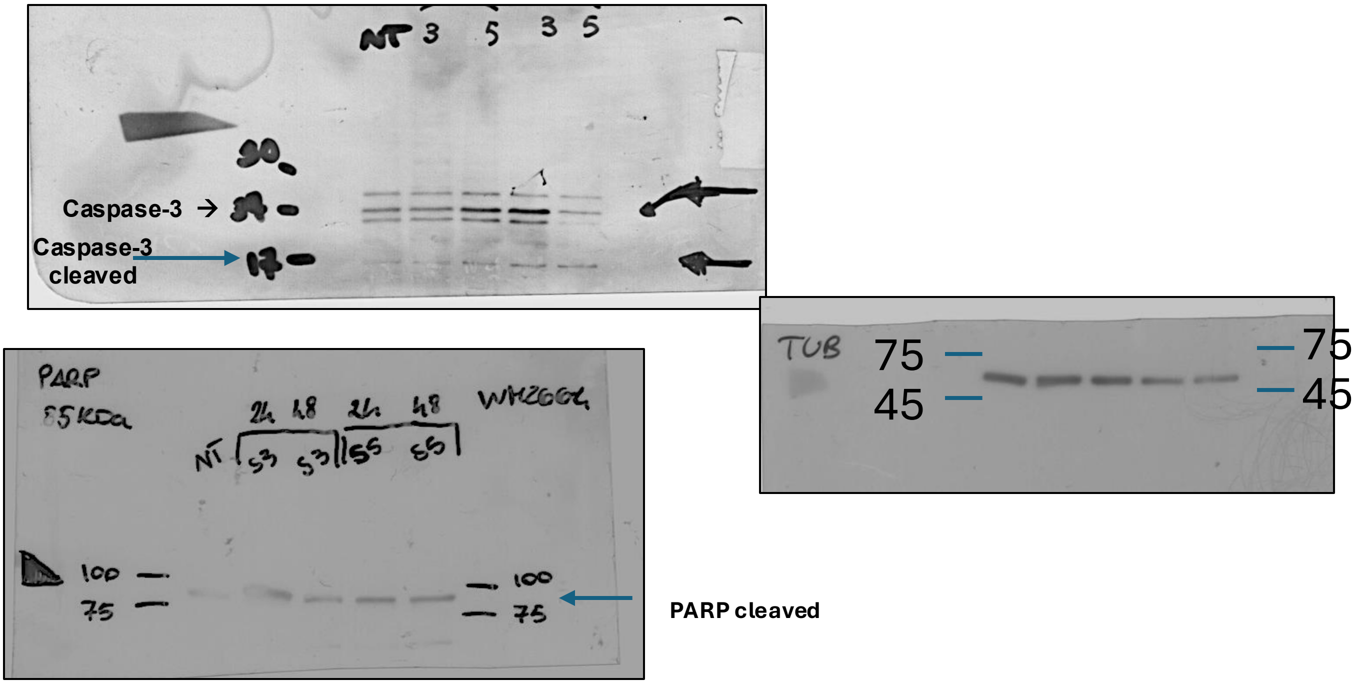

Figure 9B

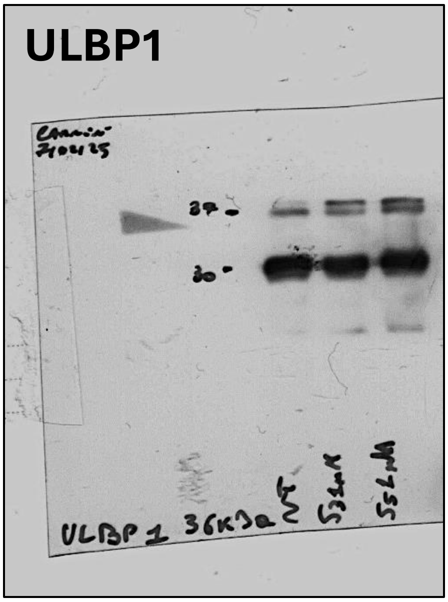

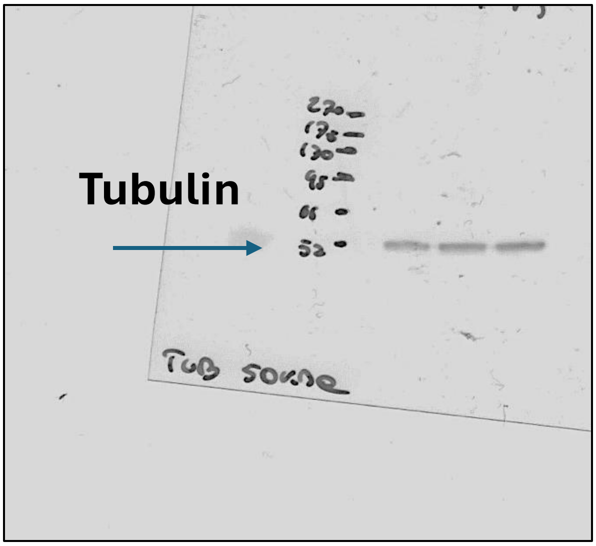

Figure 9F

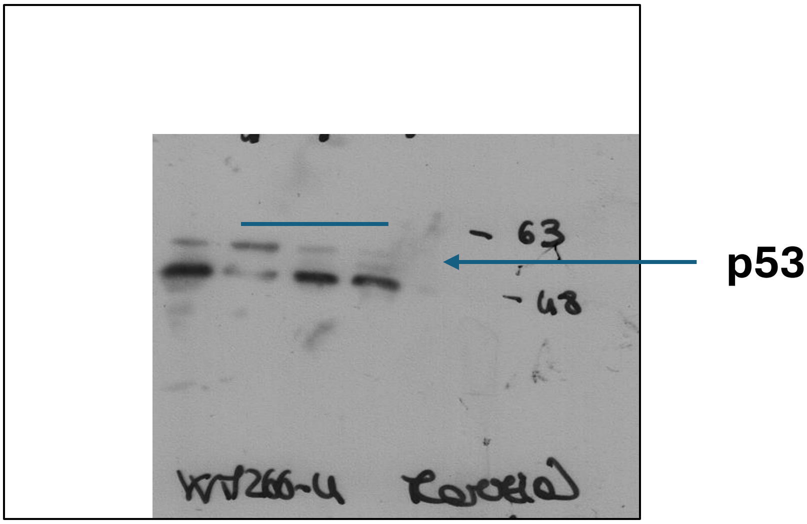

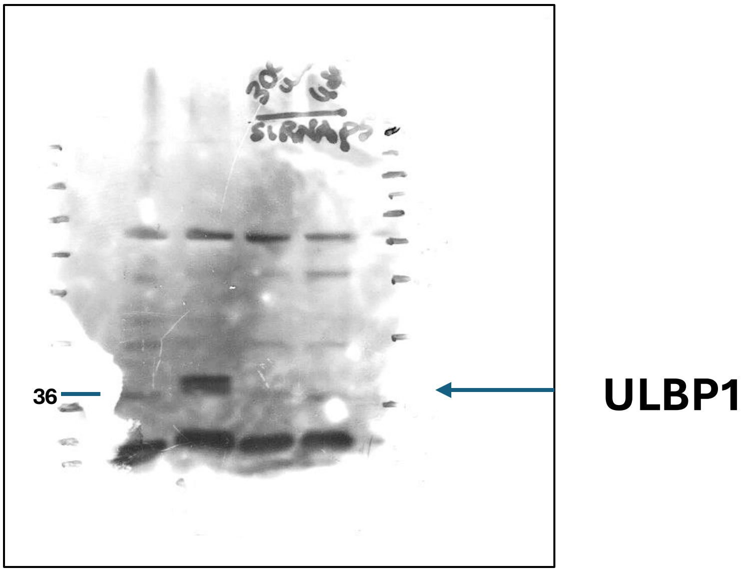

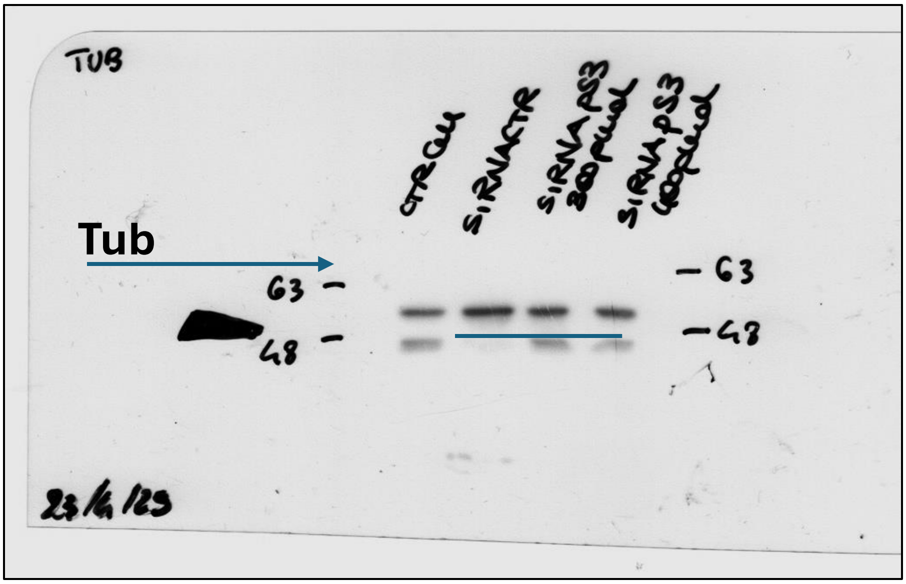

Figure 9 G

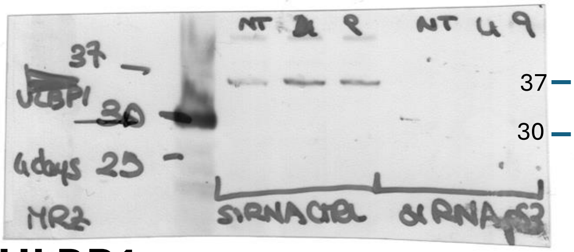

ULBP1

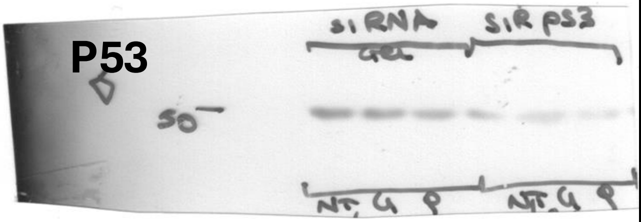

P53

Tubulin

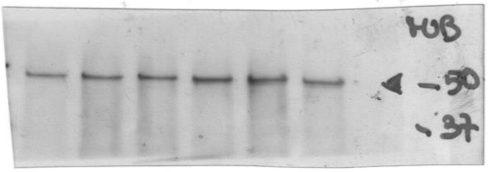

Figure 9H

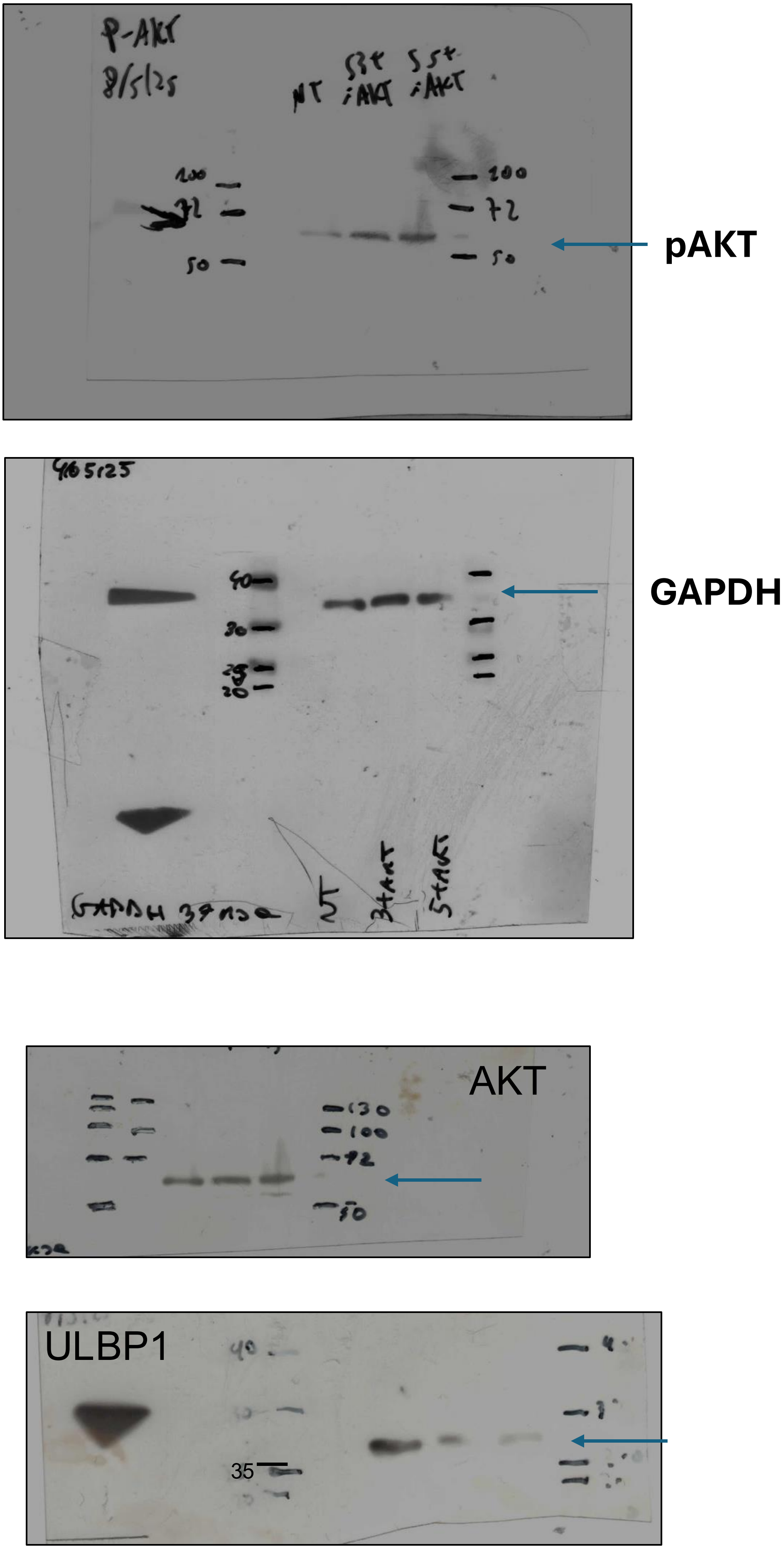

Supplemental Western Blots

Figure 1SA

TRPM8

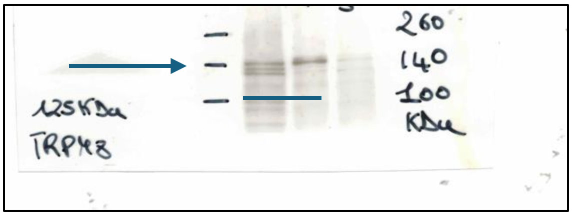

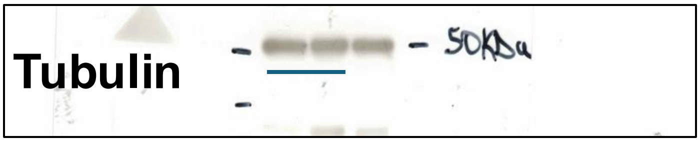

Figure 5SA

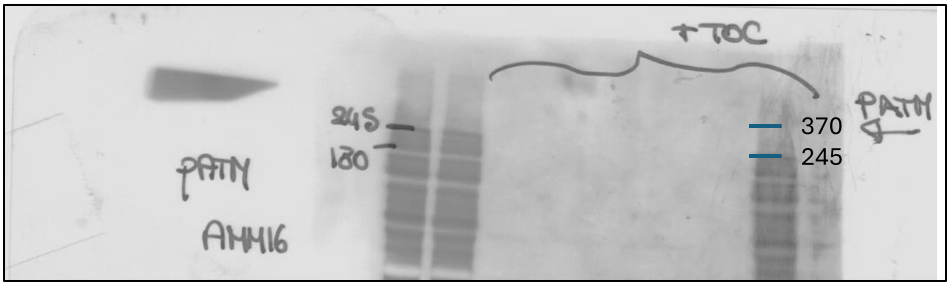
+TOC
245
180
pATM
370
245
PATM

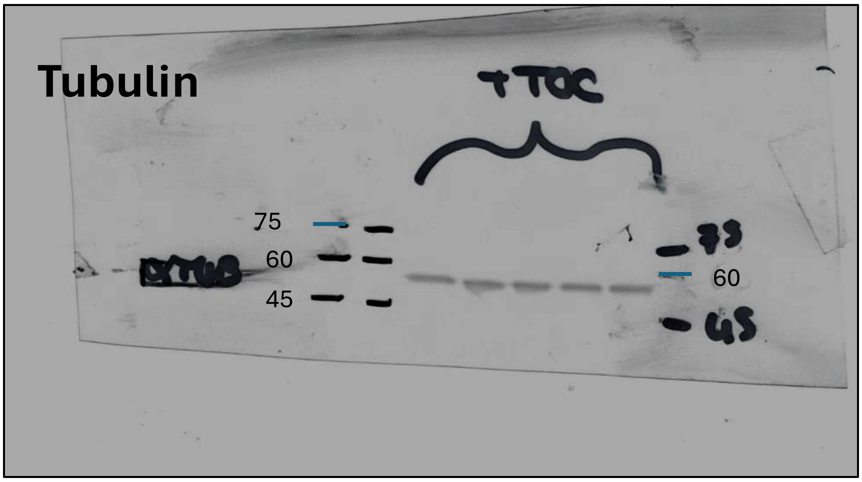
Tubulin
+TOC
75
60
45
75
60
45

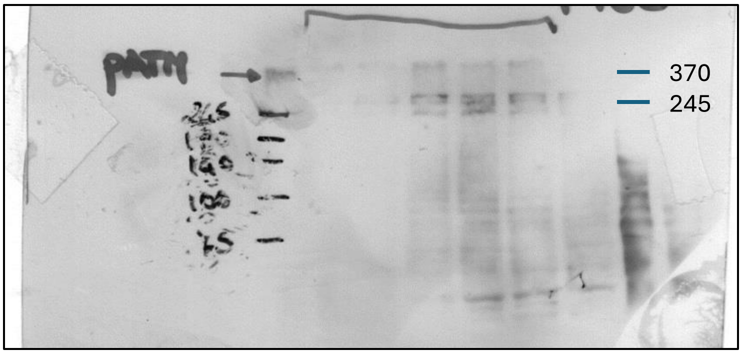
PATM
370
245

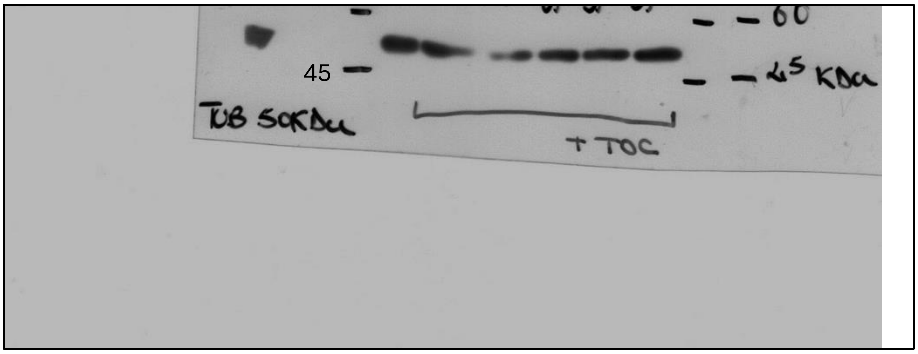
60
45
45 KDa
TUB 50KDa
+TOC

Figure 5SD

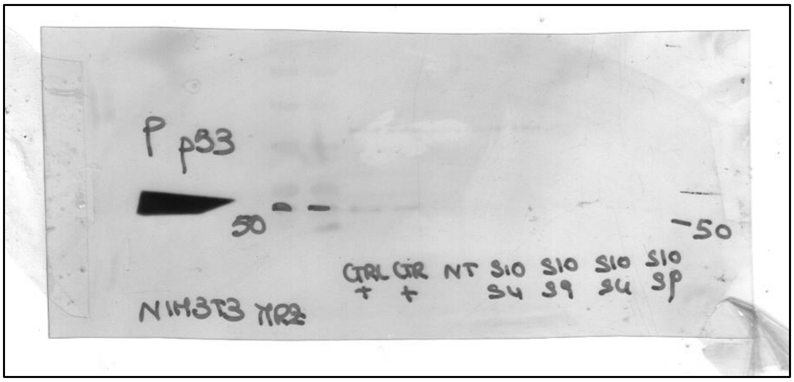
P p53
50
-50
CTRL CTR NT S10 S10 S10 S10
+ + S4 S9 S4 S9
NIH3T3

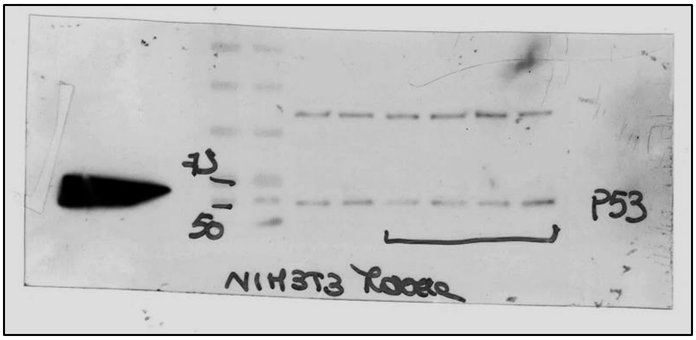
75
50
P53
NIH3T3

Figure 5SB

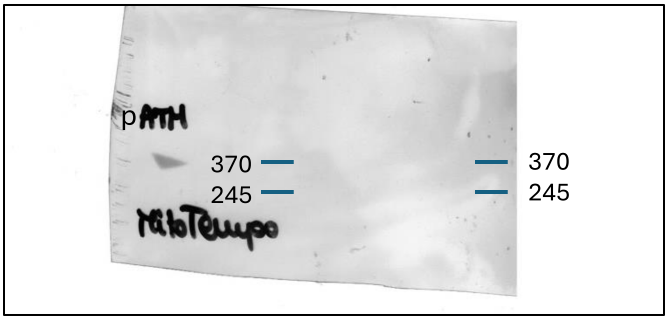

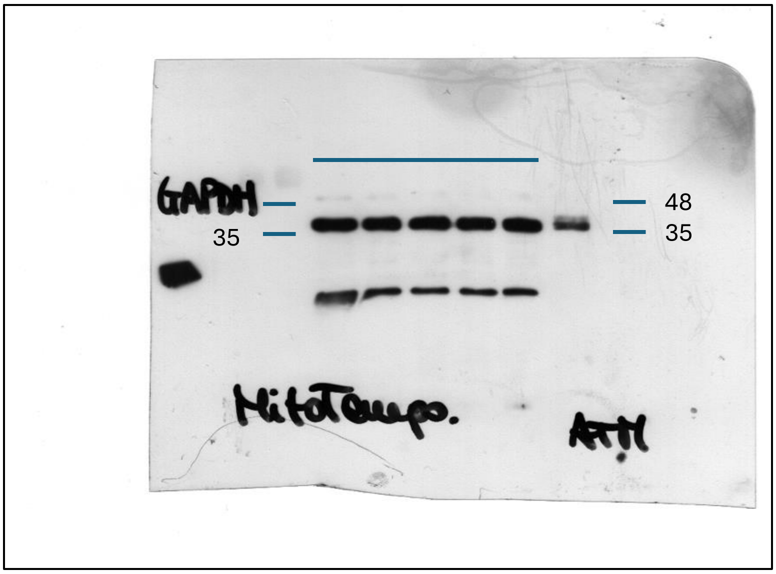

Figure 5SF

Left panel

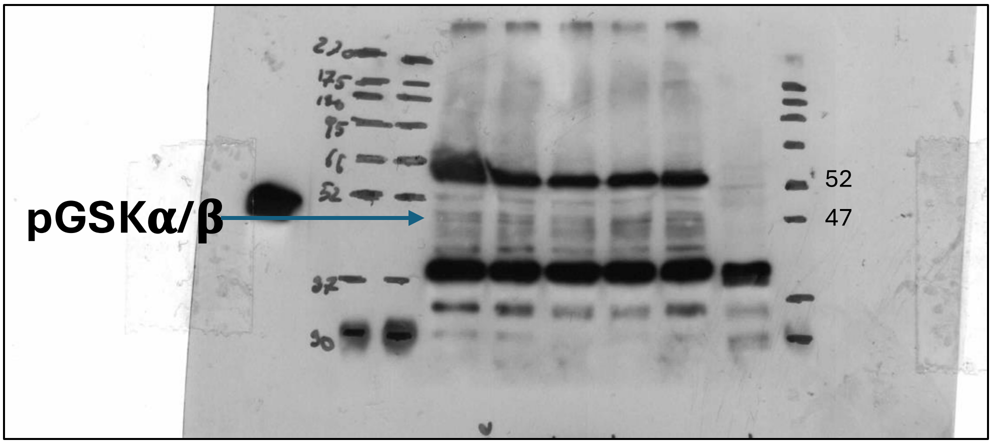

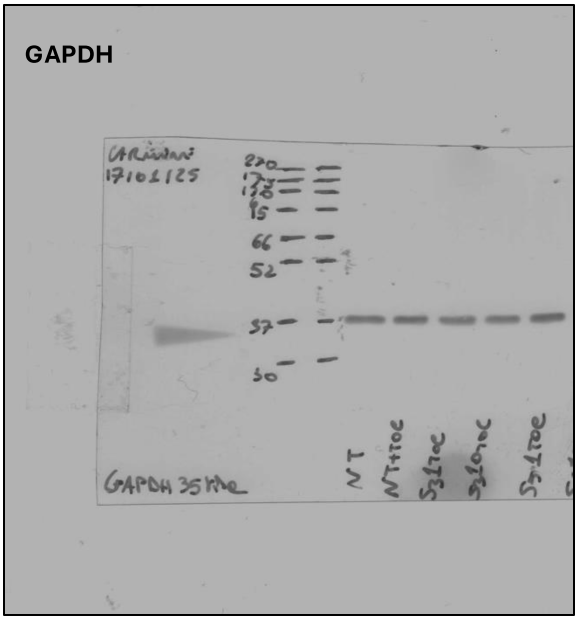

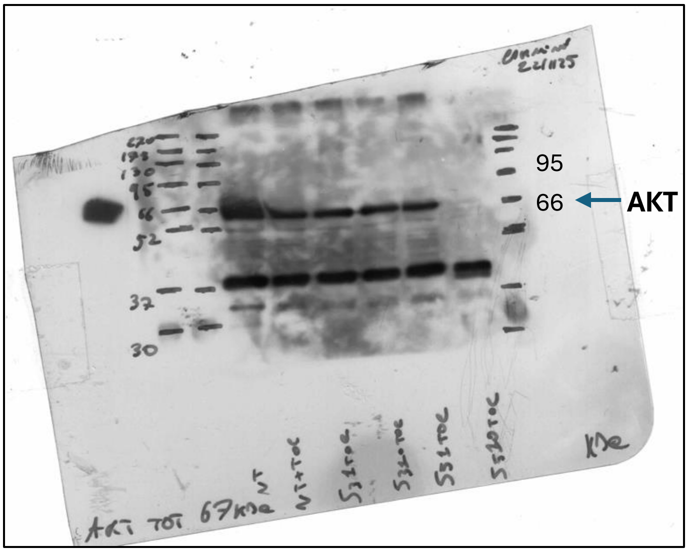

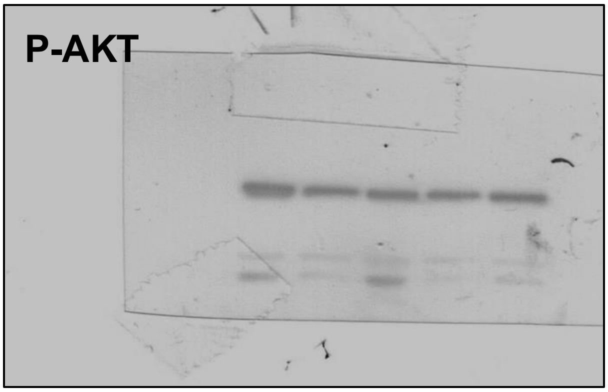

Figure 5SF

Right panel

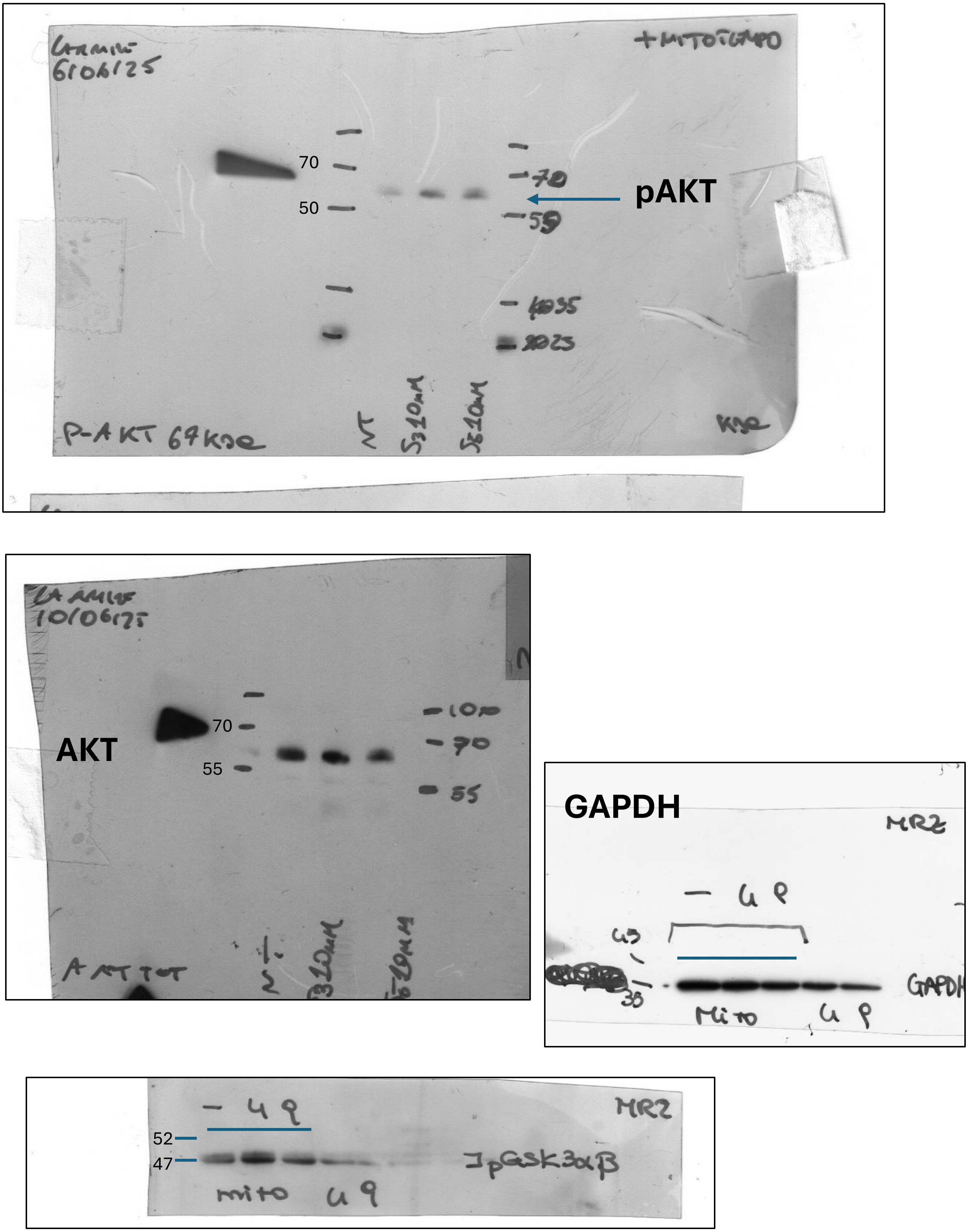

Figure 5SG

Left panel

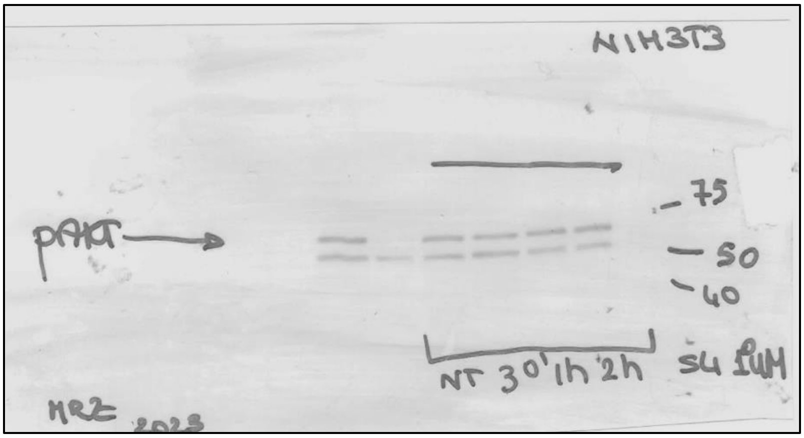

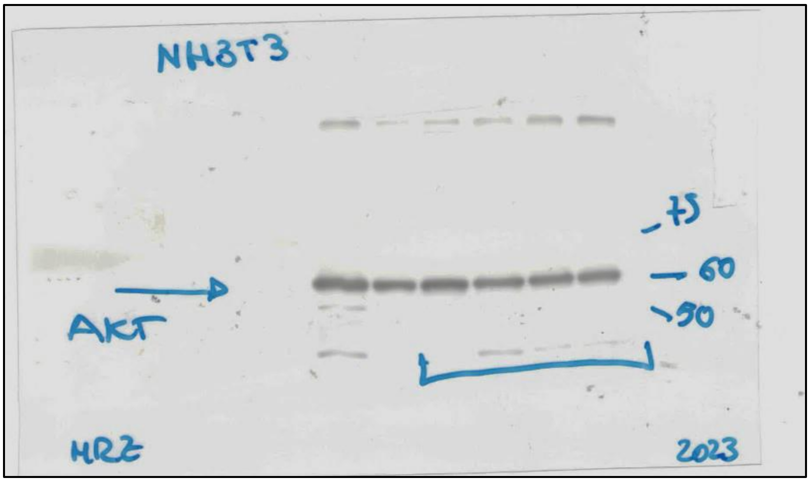

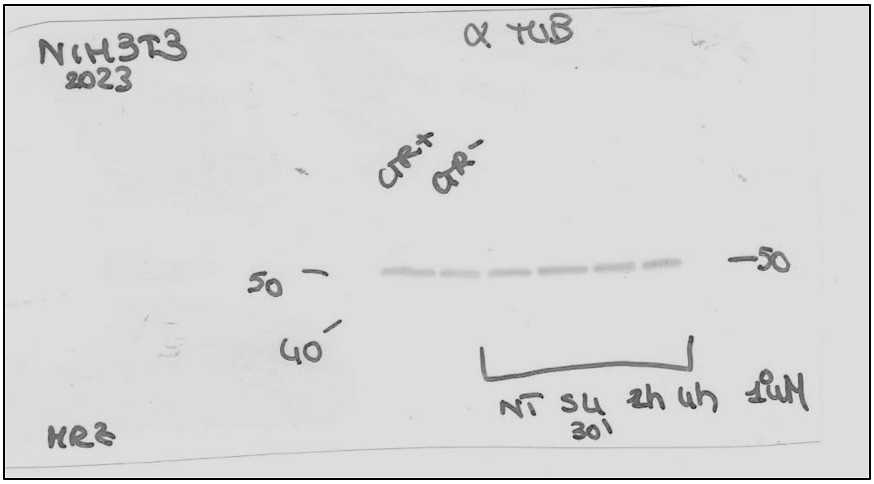

Figure 5SG

Right panel

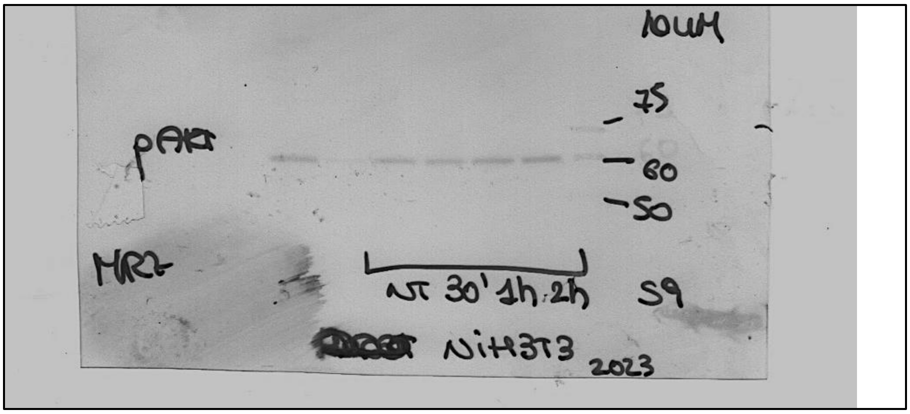

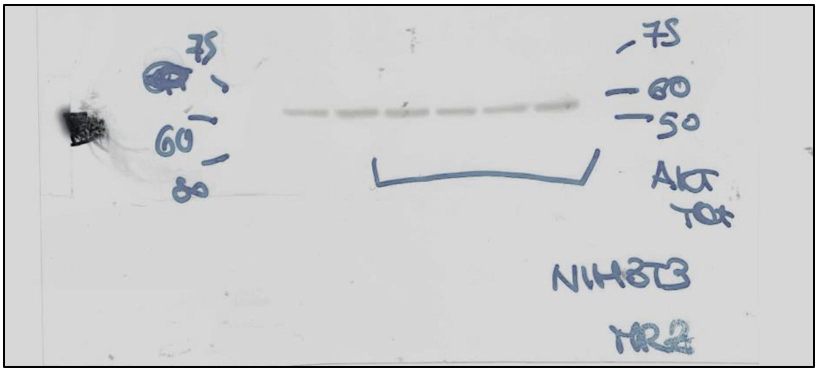

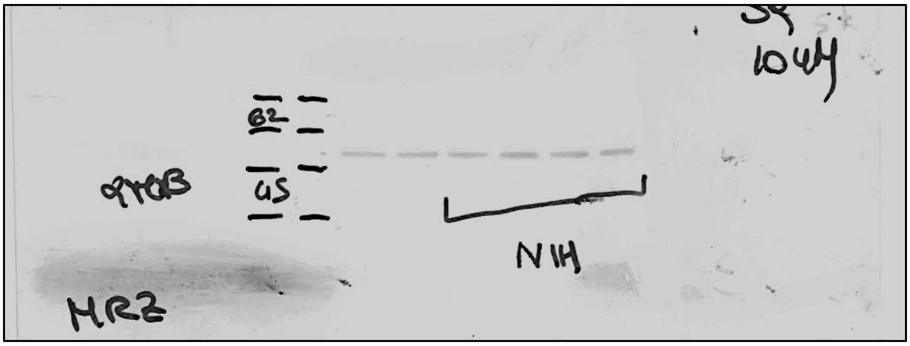

Figure 5SL

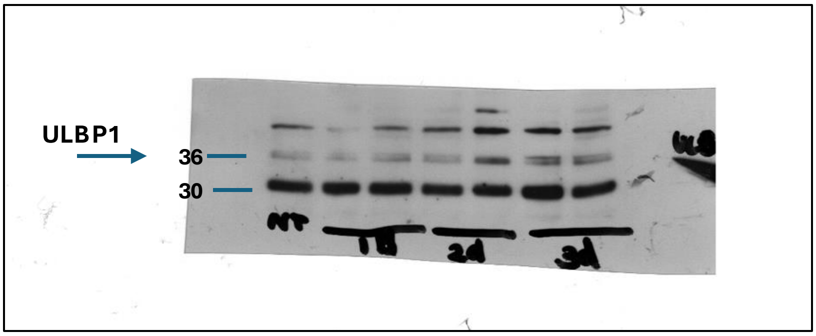

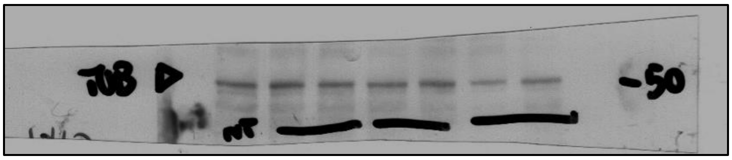

Figure 6SB

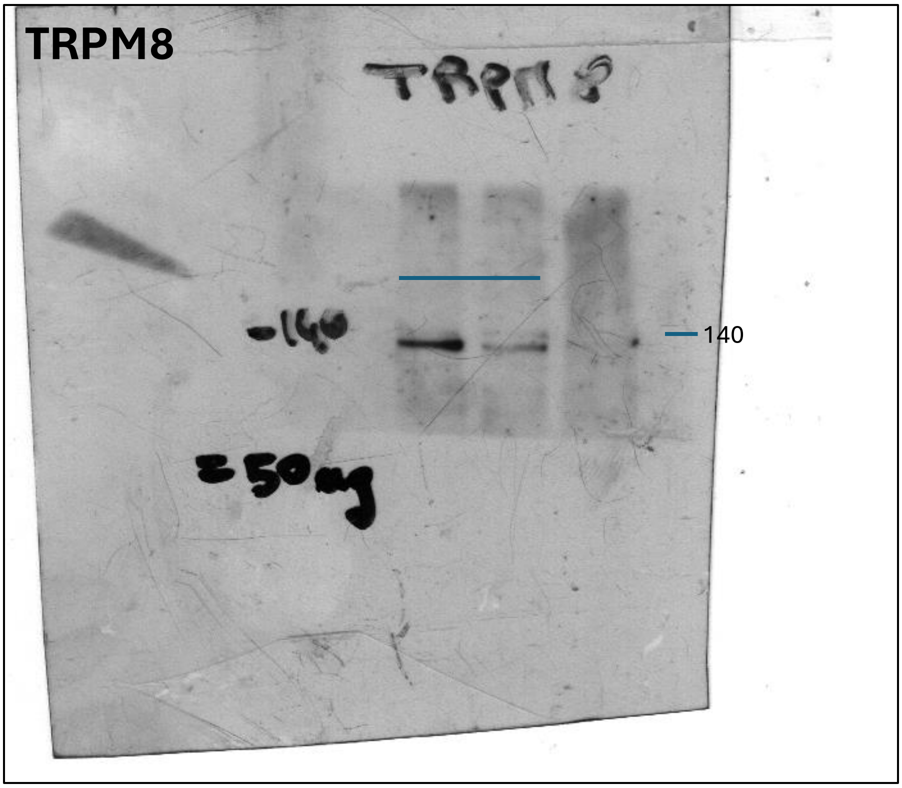

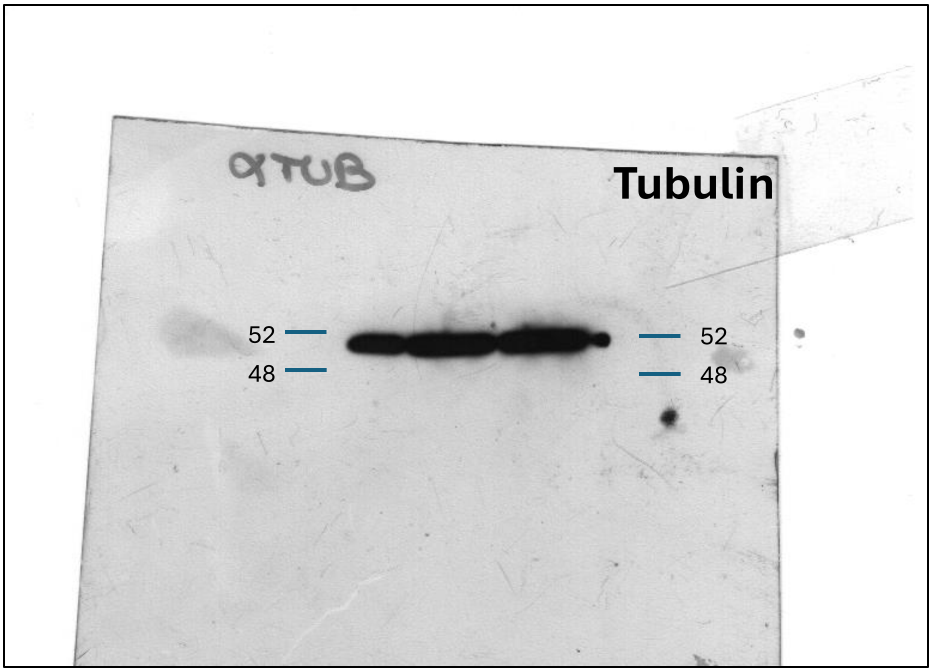

Figure 8SB

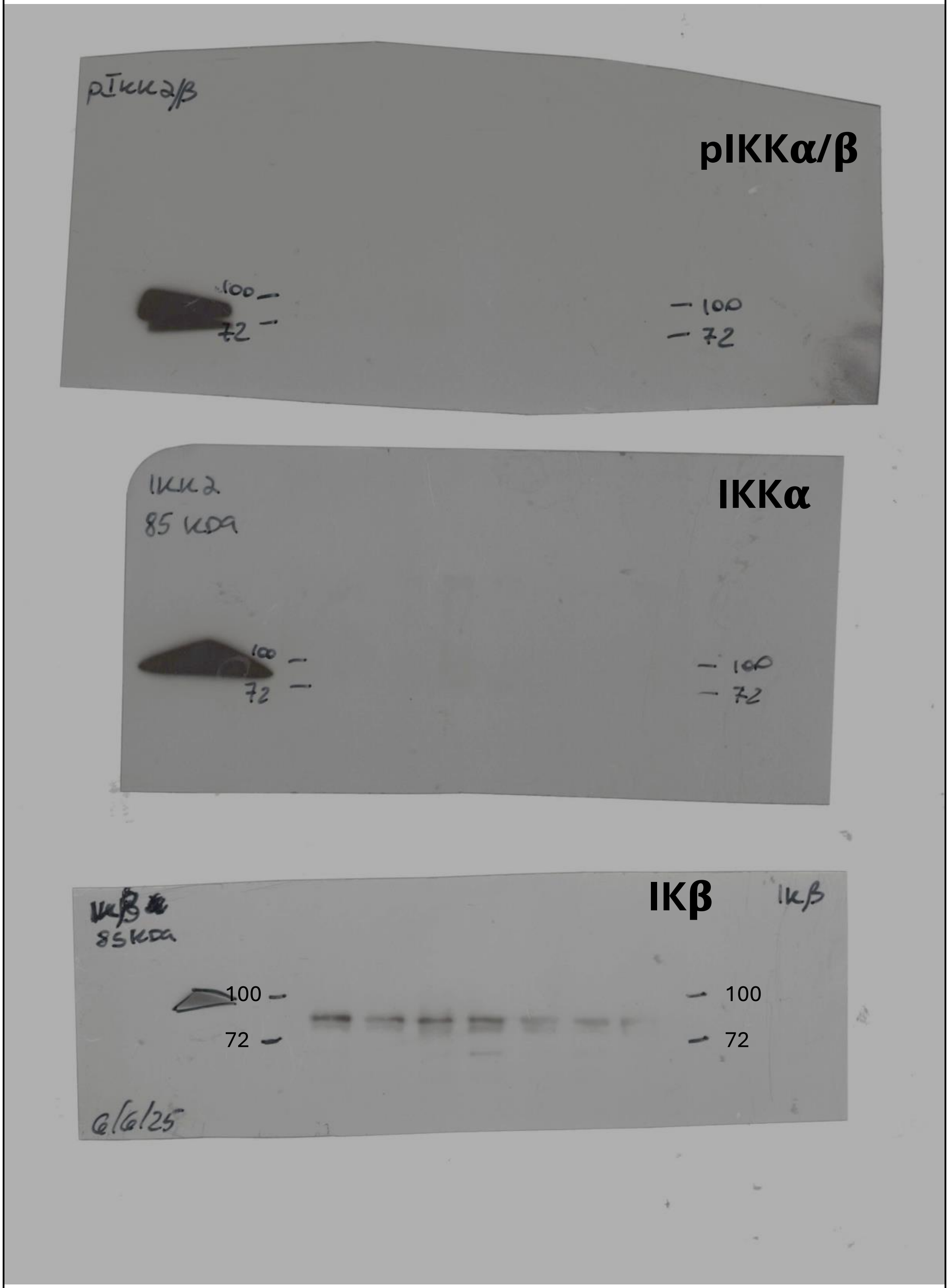
pIKKα/β
100
72
100
72
IKKα
IKK2
85 KDa
100
72
100
72
IKβ
IkB
85KDa
100
72
100
72
6/6/25

Figure 8SB

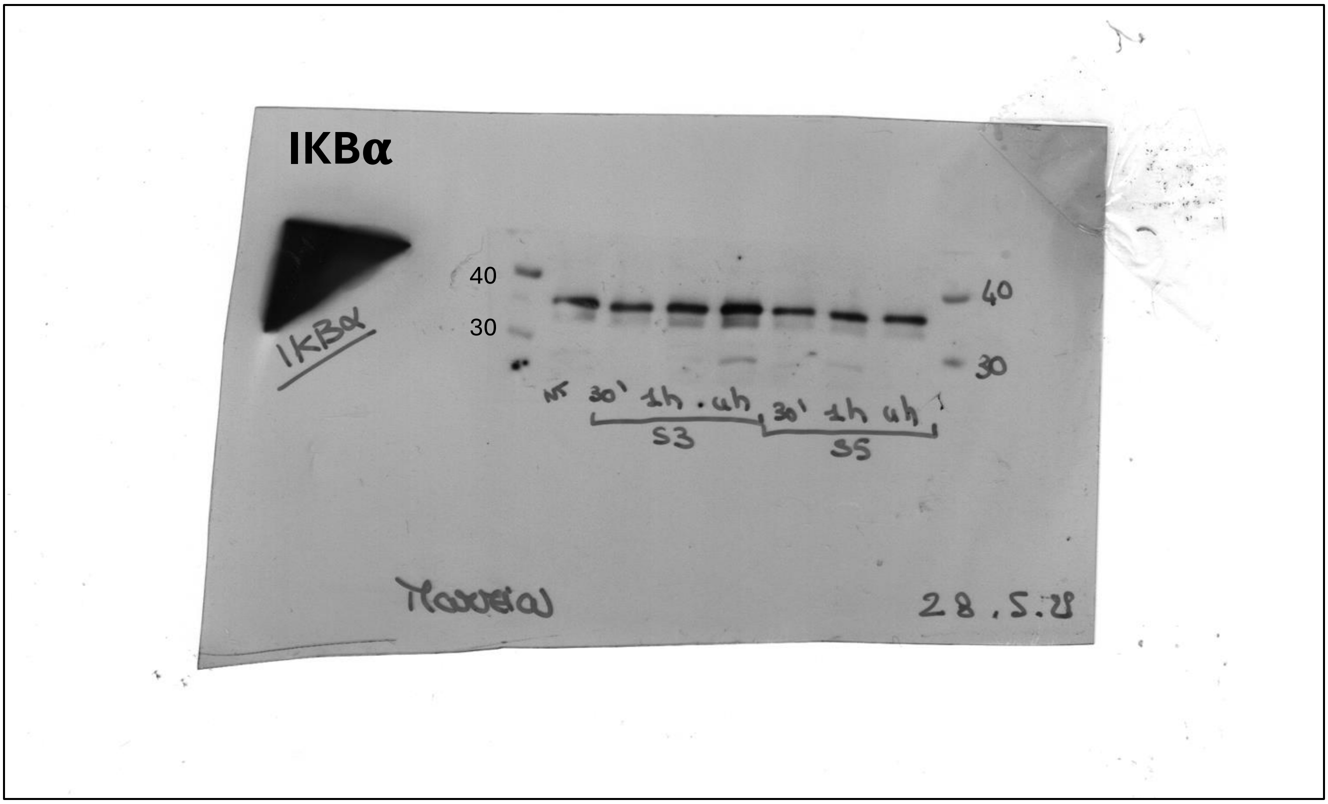
IKBα
40
30
IKBα
40
30
NT
30' 1h 4h
S3
30' 1h 4h
S5
28.5.25

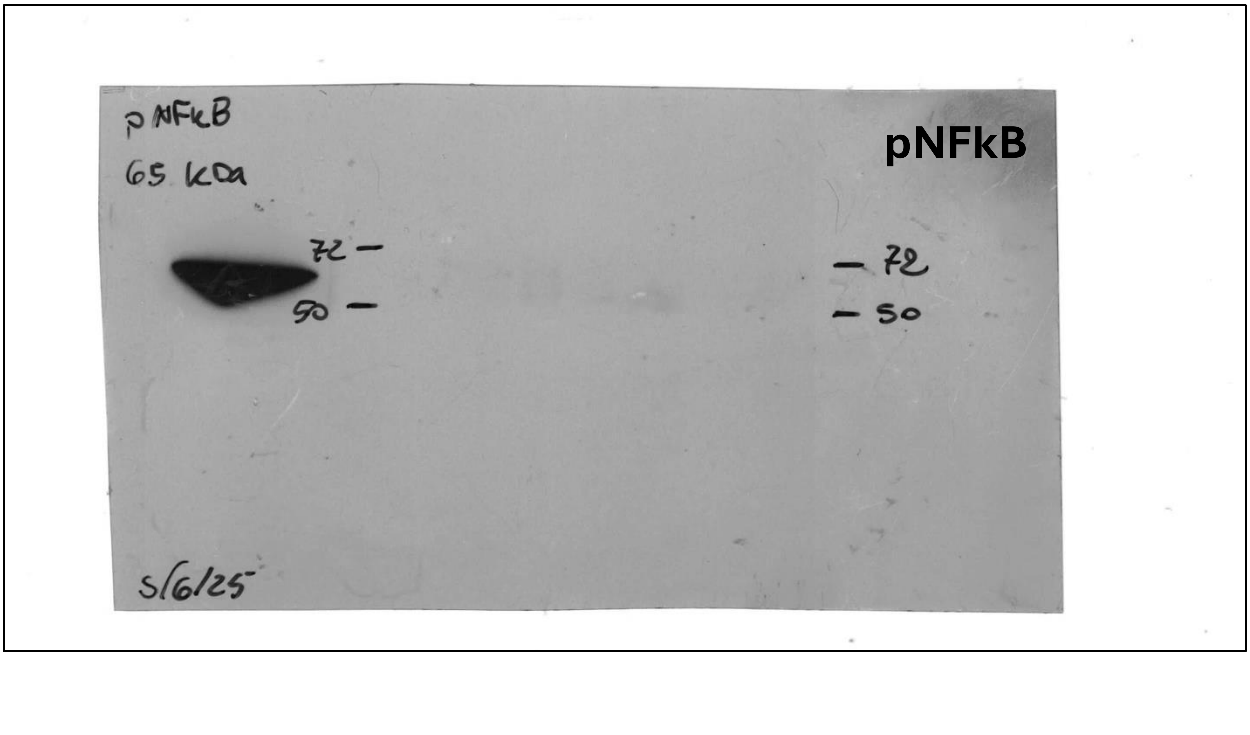
pNFkB
65 kDa
pNFkB
72
50
72
50
5/6/25

Figure 8SB

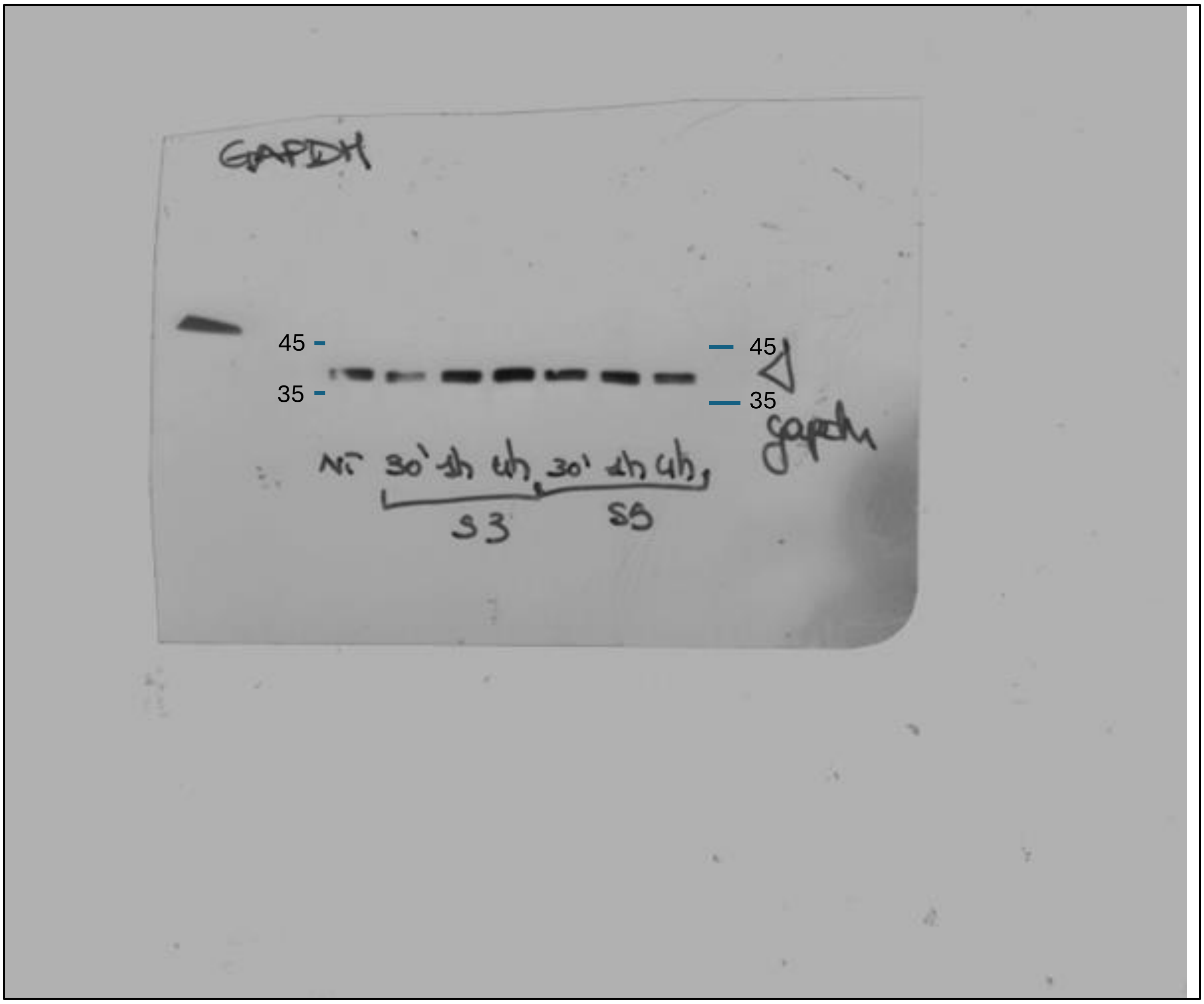

Supplement: Supplementary file 2 — Full and uncropped Western Blots [file 41419_2026_8469_MOESM2_ESM.pdf]
